# Synthesis and Evaluation of Pyridine-Based Antibacterial Agents that Inhibit ATP Synthase in *Acinetobacter baumannii*

**DOI:** 10.1021/acsomega.5c06380

**Published:** 2025-09-26

**Authors:** Angelina L. Dennison, Armaan Singh, Toni A. Marchlewski, Sierra N. Ghee, Kevin C. Gencel, Ava G. Hammock, Sabrina Liu, Shaylla Wilson, Alexander P. L. Williams, Katie T. Ward, Thomas E. Meigs, P. Ryan Steed, Amanda L. Wolfe

**Affiliations:** † Department of Chemistry and Biochemistry, 8620 University of North Carolina Asheville, One University Heights, Asheville, North Carolina 28804, United States; ‡ Department of Biology, University of North Carolina Asheville, One University Heights, Asheville, North Carolina 28804, United States

## Abstract

Multidrug resistant *Acinetobacter baumannii* (MDR AB) is a growing global health threat due to rising infection rates and lack of treatment options. Specifically, like other Gram-negative pathogens, MDR AB employs a suite of robust cellular resistance mechanisms, including reduced penetration of the outer membrane, increased efflux, target modification, and others, that greatly impede antibiotic activity even for antibiotics of last resort like colistin and tigecycline. Bacterial bioenergetics are an under-explored antibiotic target and can be selectively exploited, as demonstrated by the success of the antitubercular drug bedaquiline, which inhibits ATP synthase in *Mycobacterium tuberculosis*. While work has been done to expand the success of bedaquiline to Gram-negative pathogens like AB through quinoline derivation, modifications to the quinoline core have been minimal. Herein, we report the synthesis and evaluation of a library of trisubstituted pyridines for their ability to inhibit AB ATP synthase and act as antibacterial agents against both susceptible and MDR AB clinical isolates. From this work, four lead compounds were developed that are highly potent and selective AB ATP synthase inhibitors and act as antibiotics against MDR AB. Additionally, each of the lead compounds were found to act synergistically with colistin against AB in bacterial culture, which demonstrates the further potential of this class to be developed into potent antibiotics.

## Graphical abstract

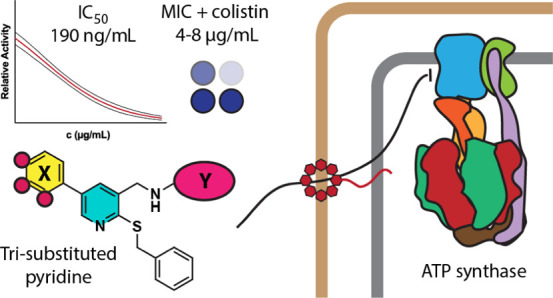

## Introduction

Antimicrobial resistance—a leading cause of death globally—was responsible for >4 million premature deaths in 2019, with projections suggesting this number could rise to over 10 million by 2050.^1,2^ The nosocomial ESKAPE pathogens (*Enterococcus faecium*, *Staphylococcus aureus*, *Klebsiella pneumonia*, *Acinetobacter baumannii*, *Pseudomonas aeruginosa*, and *Enterobacter* spp.) contribute significantly to these deaths. Multidrug resistant *A. baumannii* (MDR AB), an opportunistic Gram-negative pathogen, poses a serious challenge due to a multitude of resistance pathways, including a robust outer membrane (OM), active efflux pumps, and exiguous aperture porins. Moreover, AB is considered one of the most difficult-to-treat ESKAPE pathogens due to its resistance to safety net antibiotics (including colistin, tigecycline, and carbapenems), which demonstrates the dire need for development of novel antimicrobials.^3^

Recently, efforts in antibiotic discovery and development have shifted toward identifying new bacterial targets to overcome current resistance mechanisms in pathogens. One emerging target is ATP synthase, an essential enzyme in bacterial bioenergetics and all life (Figure 1A).^4–6^ F_1_F_o_ ATP synthase is a protein complex comprised of two rotary motors that catalyzes the final step in oxidative phosphorylation.^7^ In bacteria, the membrane-embedded F_o_ motor is composed of stator *ab*_2_ subunits adjacent to a homooligomeric ring of *c* subunits (*c*_10_ in AB).^8^ Rotation of the *c*-ring, driven by an electrochemical gradient of H^+^, is coupled to rotation of a central stalk within the cytoplasmic F_1_ motor (composed of α_3_β_3_γδε), which catalyzes phosphorylation of ADP to ATP. ATP synthase has proven to be a druggable antibiotic target through the success of the FDA-approved antitubercular drug bedaquiline (BDQ).^9^ BDQ inhibits ATP production in *Mycobacterium tuberculosis* (MT) by binding to the H^+^ binding site at the subunit *ac* interface in F_o_, halting ATP synthesis in the F_1_ complex and causing cell death.^10–12^ BDQ was the first antibiotic to target bacterial energy metabolism and the first drug approved for MDR MT in over four decades.^13^

**Figure 1:**
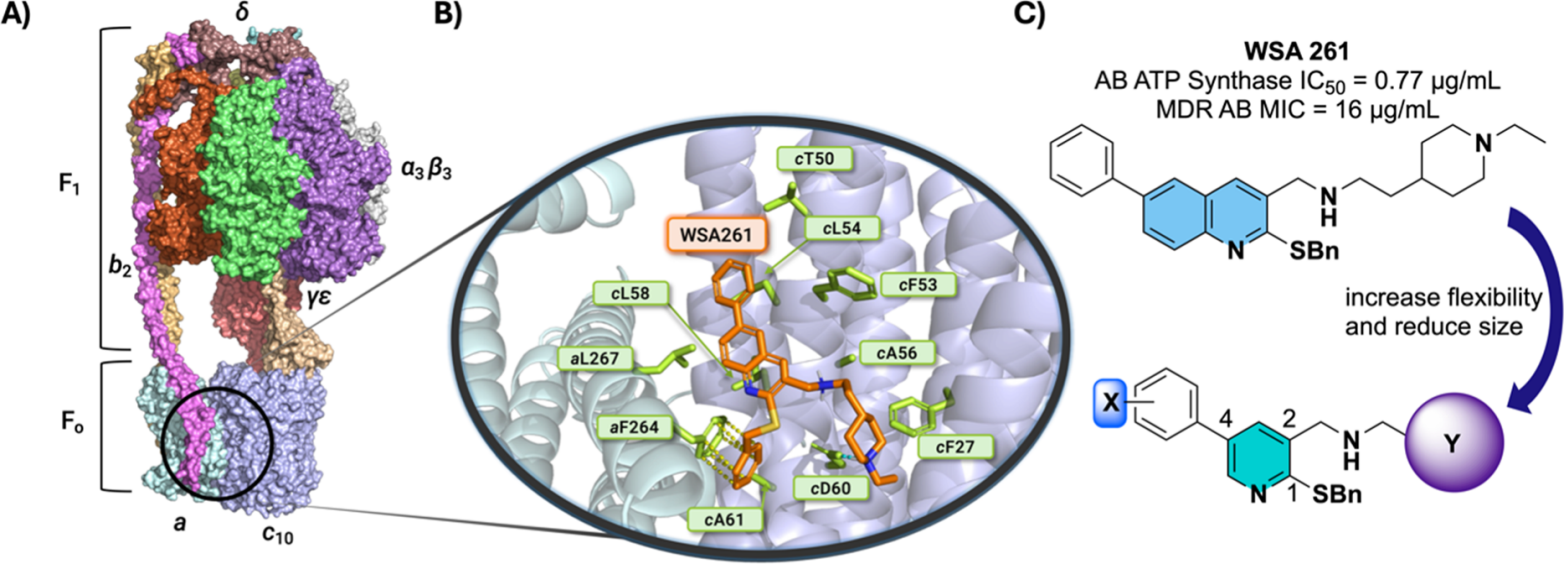
(A) *A. baumannii* ATP synthase (PDB 7P2Y).^22^ Detail shows the quinoline binding site at the *ac* interface of F_o_ with docked **WSA 261** (orange); yellow dashes indicate π-stacking interactions between the benzyl sulfide and *a*Phe264, and the blue dashes indicate salt-bridge formation between the tertiary amine and *c*Asp60. (B) Lead molecule (**WSA 261**) from the quinoline series of AB ATP synthase inhibitors. (C) Design of the pyridine series where positions X and Y are elaborated.

Due to the success of BDQ against MT, our group^14–17^ and others^18–21^ have begun to explore whether functionalized quinolines can inhibit ATP synthase in other bacterial pathogens via a similar binding interaction with the F_o_ c subunit and act as antibiotics against resistant strains. One challenge of targeting ATP synthase in Gram-negative pathogens, like AB specifically, is that the F_o_ complex of ATP synthase is embedded in the inner membrane. Therefore, the drugs must first penetrate the negatively charged OM and then embed in the hydrophobic inner membrane to inhibit the enzyme. Recently, we have developed and interrogated a series of functionalized quinolines that are able to inhibit ATP synthase in AB and penetrate the OM to act as antibiotics against MDR AB strains.^17^ Specifically, the quinoline core was substituted with a benzyl sulfide at the C1 position and a flexible and basic nitrogen-containing side chain at C2. Functionalization on the western portion of the quinoline increased enzyme inhibition and overall antibacterial activity against both susceptible and MDR AB clinical isolates. However, increasing steric bulk beyond a certain threshold decreased both enzymatic and bacterial activity significantly, which was attributed to overall larger steric bulk limiting the interactions with the c subunit and reducing membrane permeability. Quinoline **WSA 261** (Figure 1B) demonstrated the most potent antibacterial activity against both susceptible and MDR AB of the series.^17^ To explore the necessity of the quinoline core and to interrogate the limitations on molecular flexibility and size on AB ATP synthase inhibition more broadly, we have synthesized a library of 34 novel trisubstituted pyridine analogs (Figure 1C) and evaluated this library in enzymatic, antibacterial, and cytotoxicity assays to assess the viability of this series as potential antibacterial agents for treating AB infections.

## Results and Discussion

### Synthesis

While quinolines are commonly found in antibacterial molecules, highly functionalized quinolines, like BDQ, can be challenging to access synthetically and often rely on the use of toxic reagents like refluxing POCl_3_ to generate.^17^ Thus swapping the traditional quinoline core of prior ATP synthase inhibitors for a pyridine not only allows us to expand structure activity relationship (SAR) profile of bacterial ATP synthase inhibitors, it also provides a cheaper and more readily available starting material for synthetic elaboration. Using the same approach employed with the quinoline series of ATP synthase inhibitors,^17^ a pyridine library, functionalized at C1, C2, and C4, was synthesized via the 3-step sequence as shown in Schemes 1 and 2. Briefly, starting from chloropyridine **1** or **2**, nucleophilic aromatic substitution of the C1 chlorine provided benzyl sulfides **3** and **4**, respectively, in good yields. The aldehydes at the C2 position of **3** and **4** were then elaborated via reductive amination with basic amines (piperidines, pyrrolidine, and piperazine) that were found to be potent in the quinoline series to provide the first two series of compounds in the library where C4 is either an H (**WSA 264**, **265**, **267**, and **268**) or a Br (**WSA 272**, **301**, **293**, and **271**). The C4 brominated pyridines were then further elaborated via a Suzuki reaction with a variety of phenyl boronic acids to examine both the electronics and sterics at this position, which produced 26 compounds in fair to good yields (Scheme 2).

**Scheme 1:**
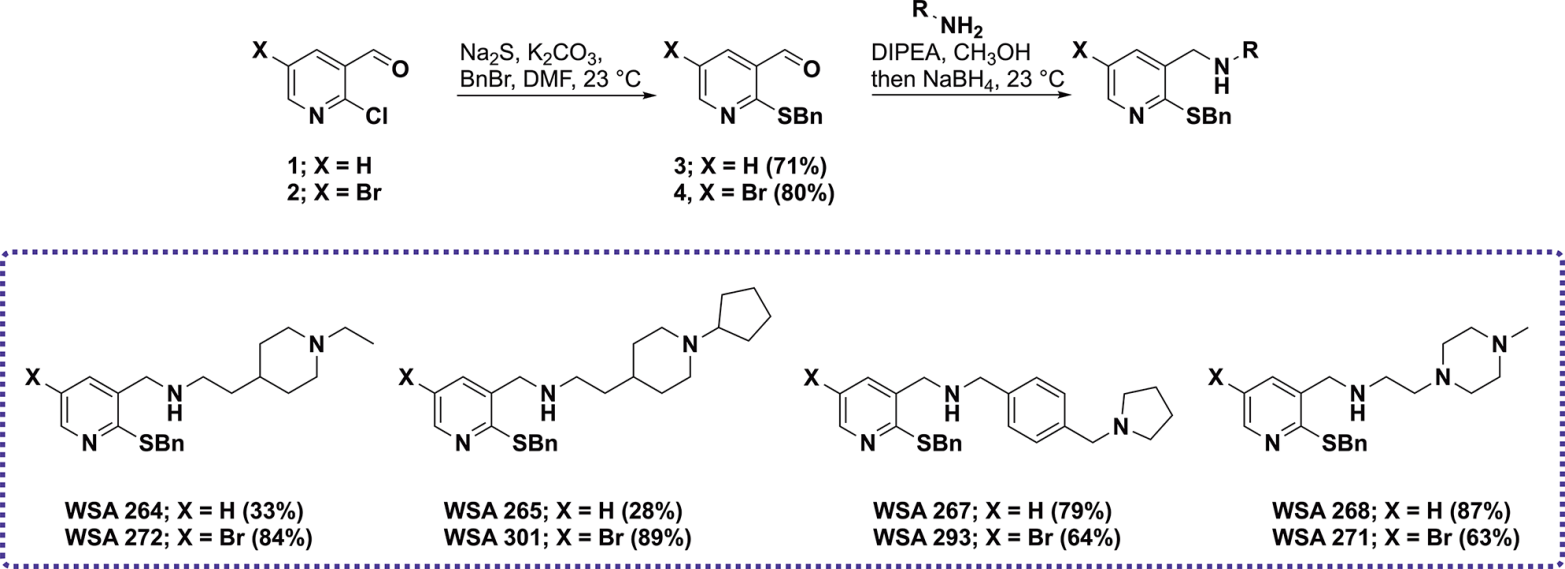
Synthesis of C4 = H/Br Pyridine Analogs

**Scheme 2:**
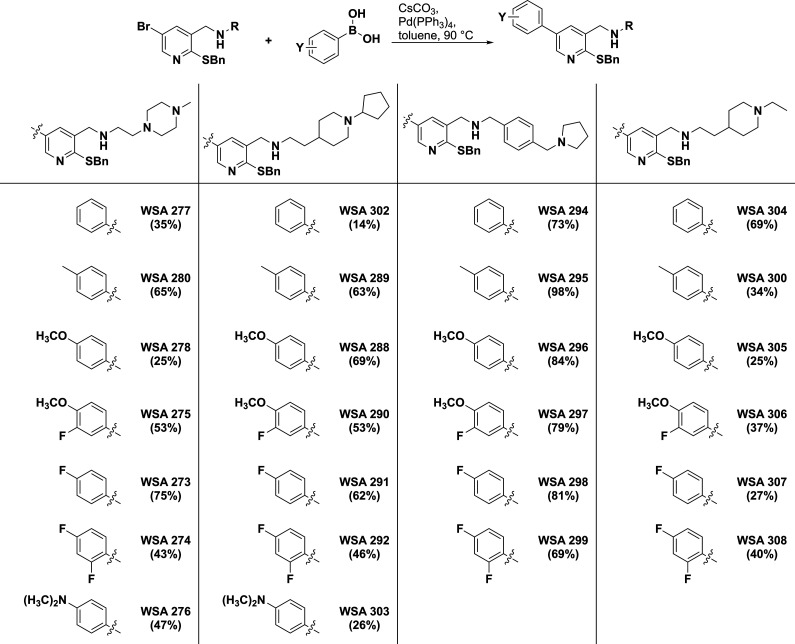
Synthesis of C4 Phenyl Pyridine Analogs via Suzuki Reaction

### ATP Synthase Inhibition

Once synthesized, the pyridine compounds were tested for their ability to inhibit ATP synthase activity using our previously reported luciferin/luciferase assay.^17^ Briefly, endogenous electron transport chains of inverted inner membrane vesicles were energized with NADH to generate a proton gradient and to drive ATP synthesis. ATP was measured via luminescence from the luciferase-catalyzed oxidation of d-luciferin. Luminescence activities across increasing inhibitor concentrations were corrected for gradient independent background sources of ATP by comparison with a control containing the protonophore CCCP (carbonyl cyanide 3-chlorophenylhydrazone). The data were then fit to a four-parameter logistic dose–response curve, from which IC_50_ values and Hill coefficients were determined using a nonlinear least-squares regression.

Assessing the SAR of ATP synthase inhibition, pyridines with H or Br at C4 exhibited the lowest inhibitory activity across all amine series (Table S1, Figure 2 and S1). Among compounds having C4 phenyl groups, the 4-methoxyphenyl, 3-fluoro-4-methoxyphenyl, and 4-methylphenyl analogs showed potent IC_50_ values across all amine types, while the 2,4-difluorophenyl, phenyl, and 4-fluorophenyl substituents generally demonstrated weaker inhibitory activity. Compounds **WSA 290**, **289**, **275**, **288**, and **305** showed the strongest inhibitory activity among the pyridines, with IC_50_ values between 190 and 270 ng/mL. These in vitro IC_50_ values are significantly lower than that of **WSA 261** (IC_50_ = 770 ng/mL), suggesting that the pyridine scaffold improves AB ATP synthase inhibitory activity. **WSA 290**, **289**, and **288** all belonged to the C2 cyclopentyl piperidine substituted series, while **WSA 275** belonged to the methyl piperazine series and **WSA 305** belonged to the ethyl piperidine series. **WSA 290** and **WSA 275**, the strongest performing compounds in their amine series, contained 3-fluoro-4-methoxyphenyl at C4; similarly, **WSA 297** (IC_50_ = 270 ng/mL) with the same 3-fluoro-methoxyphenyl C4 substituent was the strongest performing compound within the benzyl pyrrolidine series. **WSA 288** and **WSA 305** had methoxyphenyl C4 substituents, and **WSA 289** contained a methylphenyl group off C4. The presence of a methoxy group, in both ortho disubstituted and monosubstituted forms, on the C4 phenyl seems to confer stronger inhibition.

**Figure 2:**
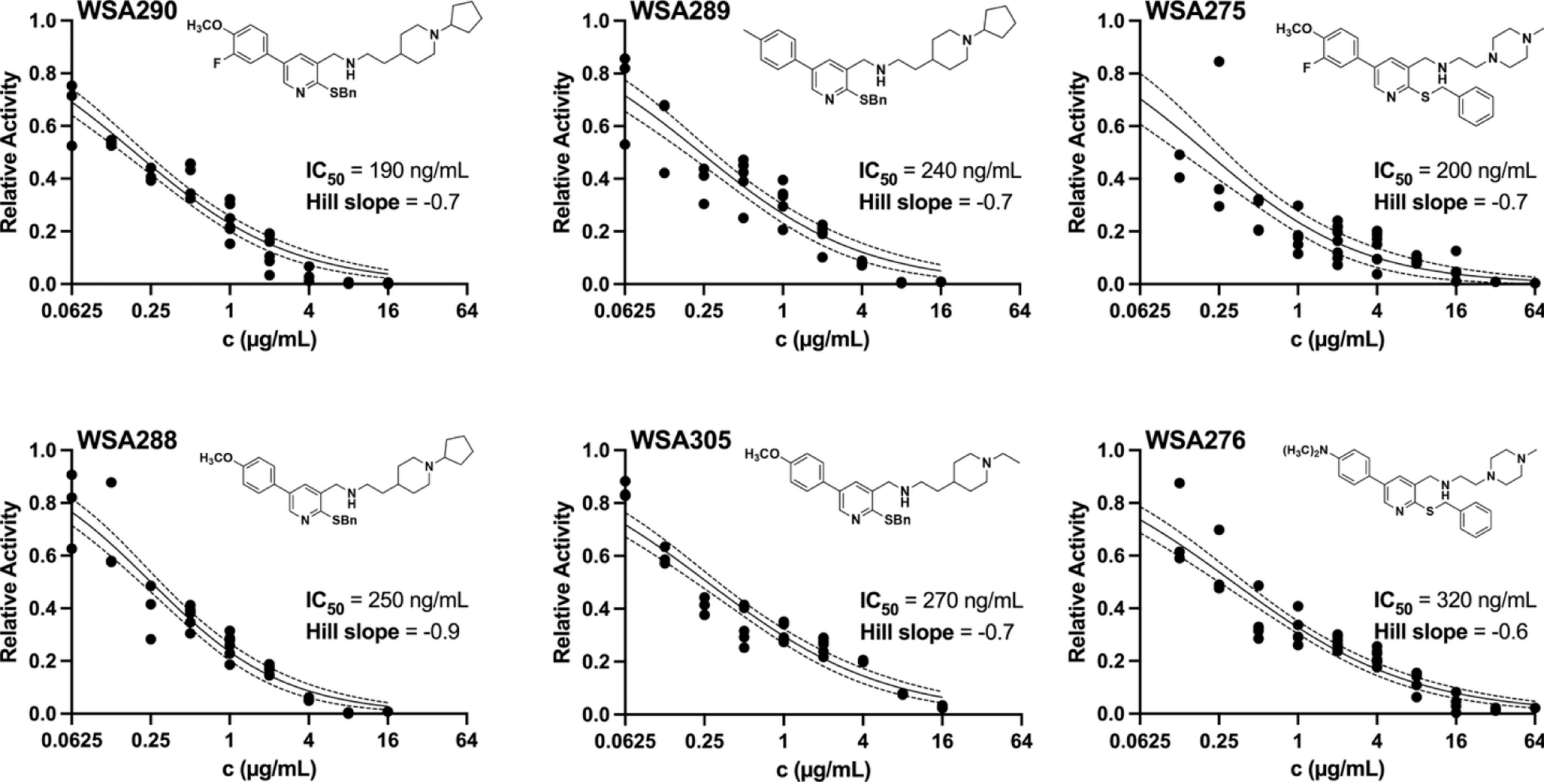
Potent inhibition of ATP synthesis activity. ATP synthesis activities of inverted membrane vesicles in the presence of 0–16 μg/mL inhibitor (log_2_ scale) are plotted relative to the DMSO control containing no inhibitor. Replicate measurements are shown as dots. The fitted dose response curves are shown as solid lines, and the dashed lines indicate the 95% confidence bounds of the fit.

In general, bulkier amine chains showed greater potency across this library of compounds, potentially allowing for greater van der Waals contact area with the *ac*_10_ binding pocket, enhancing both binding affinity and inhibitory activity. Notably, the Hill coefficients for most of the compounds were less than one, indicating more than one binding site for the inhibitor with varying affinity. If the pyridine compounds bind to the same site as the quinoline derivatives, then multiple binding sites are expected based on the binding of bedaquiline observed by cryo-EM.^10^

### Computational Docking

Computational docking was performed using GNINA and PLIP (see Methods). The protein (PDB ID: 7P2Y)^22^ was assigned protonation states using the H++ server,^23^ compounds were protonated at pH 7 using Open Babel,^24^ and both were prepared for docking using Autodock Tools. Docking was performed using GNINA with a fixed grid box centered around *c*Asp60. Poses from GNINA were then input into PLIP 2.3.0 to extract interaction level data.

The strong inhibitors **WSA 290**, **289**, **275**, **288**, and **305** all formed salt-bridges between their secondary or tertiary amines and cAsp60 (Table S2). The conformations adopted also allowed for hydrogen bonding with the backbones of *c*Phe53, *c*Gly57 (**WSA 290**), *c*Met64 (**WSA 275**), and *a*Leu257 (**WSA 305**). Additionally, *c*Ile65, *c*Leu71, *a*Ile242, *a*Leu245, *a*Ile246, and *a*Leu257 formed a hydrophobic binding pocket that consistently interacts with these compounds. The enriched binding pocket for high affinity compounds is shown in Figure 3A, where residue-ligand contacts were computed using PLIP on the chosen GNINA poses for each compound. This binding pocket overlaps substantially with that predicted for our previous quinoline scaffold (Figure 1A), supporting a conserved binding mode centered around the *c*Asp60-mediated salt-bridge and a hydrophobic cavity across both chemotypes.

**Figure 3:**
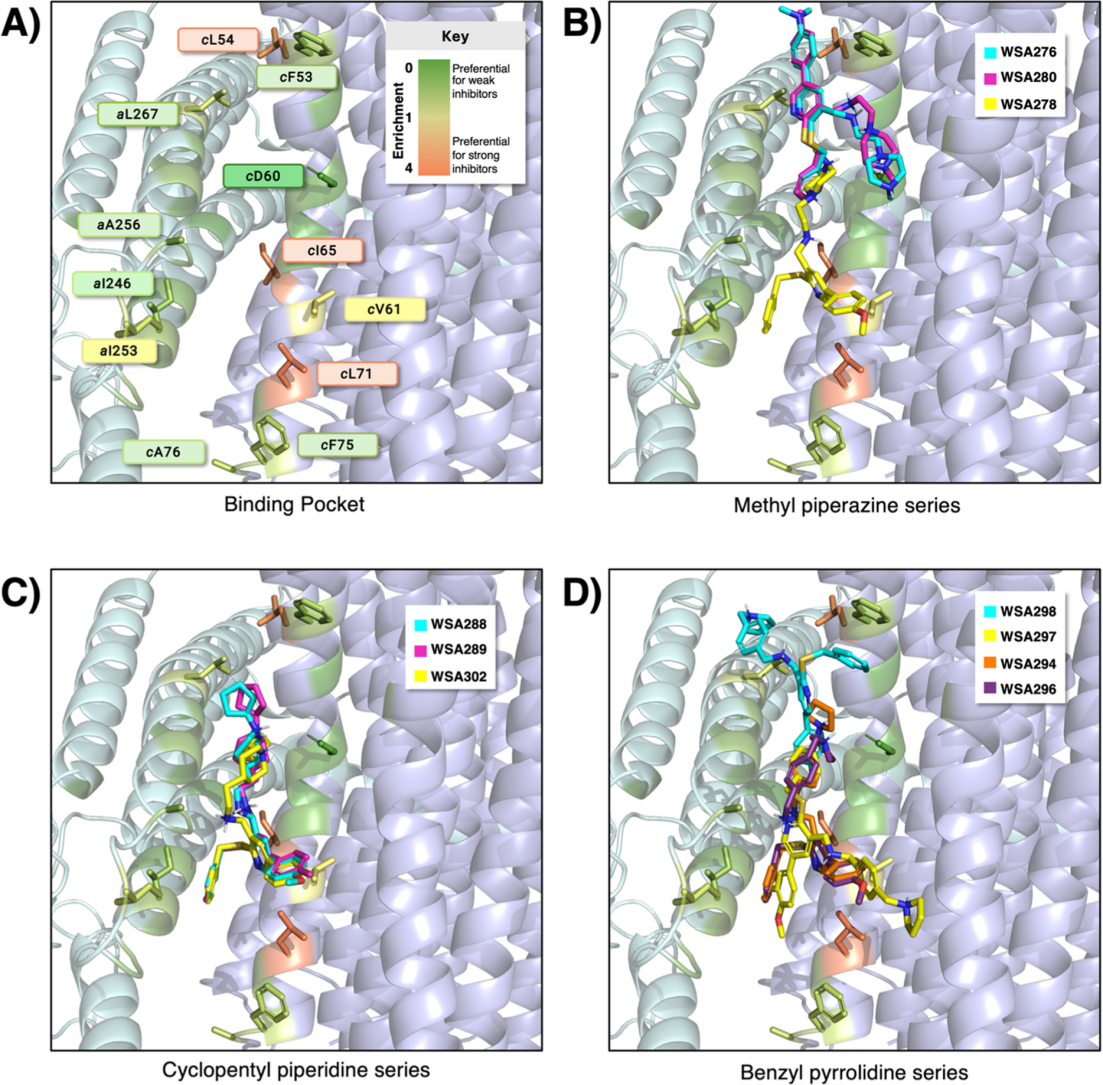
(A) The putative binding site in the *ac* interface of *A. baumannii* ATP synthase (PDB ID: 7P2Y) is shown at the interface of subunit *a* (left, pale cyan) and the *c*-ring (right, light blue). Residues in the binding pocket are colored by enrichment score, defined here as frequency of binding to strong inhibitors divided by frequency of binding to weak inhibitors, where strong inhibitors are those with IC_50_ < 500 ng/mL and weak inhibitors are those with IC_50_ > 5000 ng/mL. The residues shown as sticks are those with enrichment >1 (i.e., frequent interaction with strong inhibitors) and *c*Asp60 (a key salt-bridge forming residue). (B) Computed binding poses of compounds **WSA 276** (cyan), **WSA 278** (yellow), and **WSA 280** (magenta) in the methyl piperazine series with 4-dimethylamino, 4-methoxyphenyl, and 4-methylphenyl western fronts. (C) Computed binding poses of compounds **WSA 288** (cyan), **WSA 289** (magenta), and **WSA 302** (yellow) in the cyclopentyl piperidine series with 4-methoxyphenyl, 4-methylphenyl, and phenyl western fronts, respectively. (D) Computed binding poses of compounds **WSA 297** (yellow), **WSA 298** (cyan), **WSA294** (orange), and **WSA296** (purple) of the benzyl pyrrolidine series, with 2,4-fluoromethoxyphenyl, 4-fluorphenyl, 4-phenyl, and 4-methoxyphenyl C4 substituents respectively.

Notably, a docking overlay of high performing and low performing compounds within a particular amine series highlights some of the key differences between strong and weak binders. For instance, **WSA 278** (IC_50_ = 960 ng/mL), unlike **WSA 276** and **WSA 280** (with IC_50_ = 320 and 690 ng/mL), adopts a pose in which its C4 4-methoxyphenyl group is oriented pointing away from the binding the cavity (Figure 3B). In the cyclopentyl piperidine series, **WSA 288**, **WSA 289**, and **WSA 302** (IC_50_ = 250, 240, and 480 ng/mL, respectively) exhibit significant conformational overlap in the binding site (Figure 3C), consistent with their relatively strong inhibitory activity. Their C4 functional groups also point outward from the binding pocket, implying that this outward orientation does not solely account for the weaker potency of **WSA278**. In contrast, compounds **WSA298** and **WSA297** (IC_50_ = 650 and 420 ng/mL) from the benzyl pyrrolidine series do not form salt-bridges with *c*Asp60 in any of their nine GNINA conformations but instead engage in π-stacking interactions with *a*Trp261 and *c*Phe75. These ligands adopt alternative poses shaped by binding site complementarity and van der Waals interactions in the *ac* interface. However, **WSA294** and **WSA296** (IC_50_ = 590 and 440 ng/mL) of the same series, which do form a salt-bridge with *c*Asp60, share significant conformational overlap in the canonical binding pocket.

### Electron Transport Chain Inhibition

To ensure that the pyridine analogs were selectively inhibiting AB ATP synthase, each analog was also evaluated for inhibition of the AB electron transport chain (ETC) at 2 μg/mL using a standard protocol.^15–17^ Briefly, respiratory dehydrogenase complexes were energized with NADH to initiate redox-driven H^+^ pumping into the vesicle lumen, which was in turn detected by the quenching of 9-amino-6-chloro-2-methoxyacradine fluorescence. Most compounds were tested at concentrations of 2 μg/mL to establish whether ETC inhibition occurred in the same range as ATP synthase inhibition (Figure S2). While none of the compounds inhibited AB ETC by more than 50% at 2 μg/mL, the C2 piperazine with fluorine substituents on the phenyl of C6 (**WSA 273**, **WSA 274**, **WSA 275**) demonstrated the highest ETC inhibition of 40% at 2 μg/mL. However, this level of inhibition is well below that observed in the ATP synthase inhibition assay, which indicates that the compounds in this class are selective AB ATP synthase inhibitors.

### Antibacterial Activity

To assess whether the observed AB ATP synthase inhibition translates to antibacterial activity, the pyridine library was evaluated for antibacterial activity against both susceptible (ATCC 17978) and MDR (BAA 1605) AB clinical isolates using a standard broth microdilution assay. As shown in Table 1, which is organized by C2 amine side chain, variation at the C2 and C4 position impacted antibacterial activity against both AB clinical isolates. The most potent analogs of the entire series were **WSA 276**, **288**, **289**, **298**, and **300** with MICs of 16 μg/mL and 32 μg/mL against AB ATCC 17978 and MDR AB BAA 1605 respectively. While many of the pyridine derivatives exhibited strong AB ATP synthase inhibition in vitro, reduced antibiotic accumulation through poor OM penetration and rapid efflux can make even the most potent enzymatic inhibitors ineffective antibiotics against Gram-negative bacteria.^25–29^

**Table 1:**
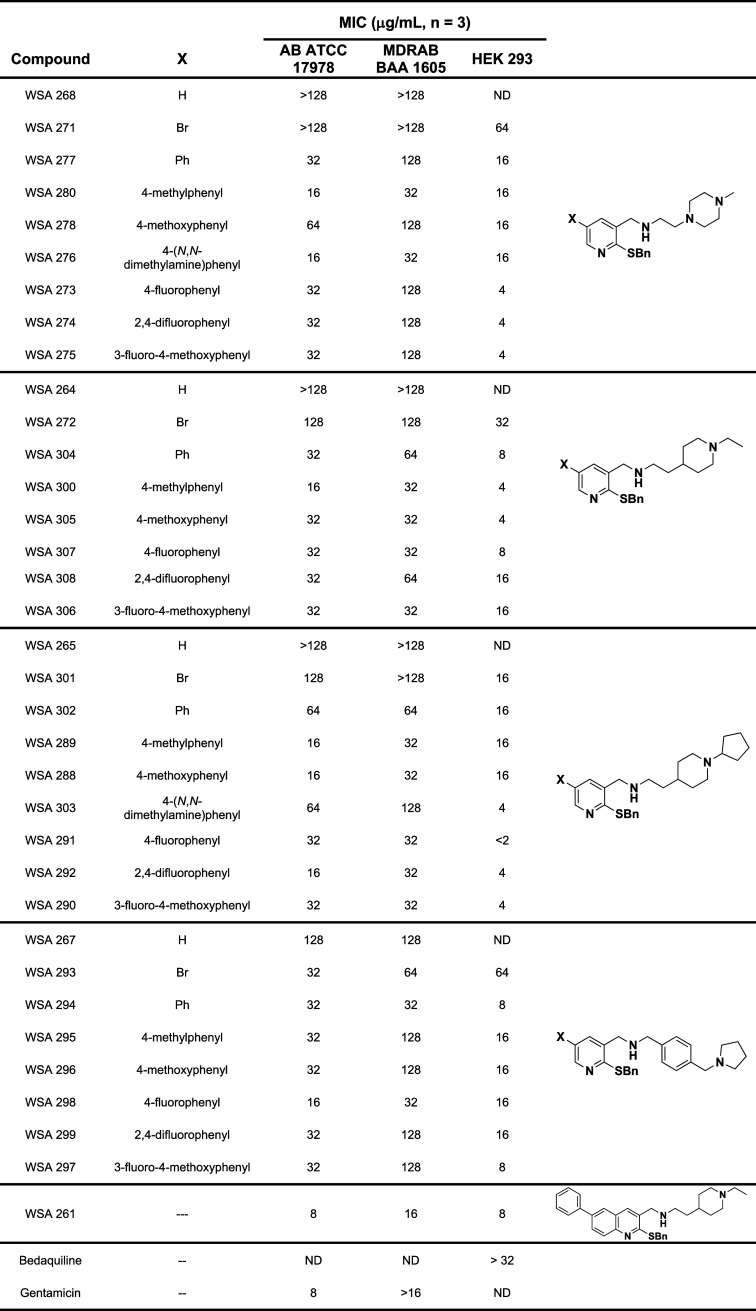
Antibacterial Activity against *A. baumannii* and Cytotoxicity against HEK 293 Cells of Pyridine Library Compared to Quinoline **WSA 261** ^a^

a MIC = minimum inhibitory concentration, defined as no visible growth and a >80% reduction in pathogen growth with compound compared to pathogen alone (DMSO only) as measured by OD 590 nm; ND = not determined.

As observed with ATP synthase inhibition, pyridine analogs where C4 is an H or Br (**WSA 268**, **271**, **264**, **272**, **265**, **301**, **and 267**) showed little to no antibacterial activity against either strain. Only the **WSA 293** with 4-(1-pyrrolidinylmethyl)­benzylamine at C2 and a Br at C4 was shown to have weak antibacterial activity against AB. Of the C2 amines, the cyclopentyl piperidine series (**WSA 302**, **289**, **288**, **303**, **291**, **292**, and **290**) was the most potent overall against both susceptible and MDR AB strains. The methyl piperidine series (**WSA 277**, **280**, **278**, **276**, **273**, **274**, and **275**) had the biggest difference in activity between susceptible and MDR AB strains with 5 of the 7 having a ≥4-fold loss in activity against MDR AB. Of the C4 benzene series, the 4-methylphenyl group elicited the best activity for all the C2 amines except for the 4-(1-pyrrolidinylmethyl)­benzylamine, which had the best activity with the 4-fluorophenyl substituent, indicating that overall molecular size affects antibacterial activity of this series similarly to the quinoline series.^17^ This trend is also demonstrated when comparing **WSA 276** and **WSA 303**, which both have a 4-(*N*,*N*-dimethylamine)­phenyl at C4. **WSA 276** has a smaller C2 amine, and thus this is the best of the series. Whereas **WSA 303** has the largest C2 amine, and this analog is one of the worst of the series against both strains of AB. Disubstitution of the C4 phenyl group (**WSA 274**, **275**, **308**, **306**, **292**, **290**, **299**, and **297**) had no significant or consistent impact on antibacterial activity compared to the corresponding monosubstituted C4 phenyl group analogs (**WSA 273**, **278**, **307**, **305**, **291**, **288**, **298**, and **296** respectively).

### Cytotoxicity against HEK 293

To further assess the pyridine library for their potential as antibiotics, each analog with a derivatized C4 position was assessed for mammalian cytotoxicity against Human Embryonic Kidney (HEK) 293 cells using a standard XTT ((2,3-bis-(2-methoxy-4-nitro-5-sulfophenyl)-2*H*-tetrazolium-5-carboxanilide)) cell viability assay protocol (Table 1) with BDQ as negative control (MIC > 32 μg/mL). Disappointingly and surprisingly, all of the pyridine analogs had MICs against the HEK 293 cells at or below their observed MICs against AB. The C2 cyclopentyl piperidine and ethyl piperidine series demonstrated the highest levels of cytotoxicity of the C2 amines, but there was no trend related to the C4 substitution other than that cytotoxicity paralleled antibacterial activity trends. Future studies will focus on the mechanism of cytotoxicity with the goal of improving selectivity for bacterial cells over mammalian cells.

### Potentiation with Colistin Sulfate

Despite many of the pyridine analogs having higher activity against AB ATP synthase in vitro compared to the original quinoline series,^17^ none were as potent as the lead quinoline analogs (like **WSA 261**, Figure 1) against either the susceptible or MDR clinical isolates of AB. Additionally, the high cytotoxicity observed against the HEK 293 cells demonstrated that lower dosing of the pyridines would be needed for these to be viable antibiotics. Together these observations indicated that perhaps the pyridines were not exhibiting their full antibiotic potential due to poor accumulation or other cellular resistance mechanisms. To probe this possibility and assess if antibacterial activity of the pyridines could be improved through codosing, **WSA 276**, **280**, **288**, **289**, and **298** were evaluated in a checkerboard assay with colistin sulfate. Colistin is a bactericidal, cationic cyclic lipopeptide from the polymyxin family that targets the OM of AB by electrostatically binding to the negatively charged lipid A component of lipopolysaccharides. Colistin’s hydrophobic fatty acid chain then inserts into the membrane increasing permeability, which ultimately leads to disruption of both outer and inner membranes, leakage of intracellular contents, and bacterial cell death. Because colistin increases outer membrane permeability, we hypothesized that it could improve activity of the pyridine analogs by increasing cellular accumulation, as has been observed with other antibiotics when coadministered with colistin.^30,31^ **WSA 276**, **280**, **288**, **289**, and **298** analogs were chosen because they were the most potent of the library against both the susceptible and MDR strains of AB and their MIC against the HEK 293 cells was equal to their susceptible AB MIC, which made them the most viable lead compounds. Quinoline lead **WSA 261** was evaluated for comparison because it also matched the two criteria. As seen in Table 2 and Figure S3, **WSA 288** and **289** were synergistic (fractional inhibitory concentration index, FICI ≤ 0.5) with colistin at 0.5 μg/mL which lowered their MIC against AB (ATCC 17978) by 4-fold to 4 μg/mL. Against the MDR AB (BAA 1605) synergy between **WSA 276**, **288**, and **298** and colistin at 0.25 μg/mL was observed. All other combinations, including colistin with quinoline **WSA 261**, showed additivity with colistin (FICI 0.5 < *x* < 1). These results suggest that the pyridines may be limited by OM permeability, which if overcome, would also improve their efficacy to toxicity profile.

**Table 2:** Potentiation of Pyridine Compounds by Colistin Sulfate

	antibacterial activity (MIC, μg/mL)					
	AB ATCC 17978			AB BAA 1605		
compound	alone	+colistin	fold change	alone	+colistin	fold change
**WSA 276**	16	8 (0.5 μg/mL Col)	2	32	8 (0.25 μg/mL Col)	4
**WSA 280**	16	8 (0.5 μg/mL Col)	2	32	16 (0.125 μg/mL Col)	2
**WSA 288**	16	4 (0.5 μg/mL Col)	4	32	8 (0.25 μg/mL Col)	4
**WSA 289**	16	4 (0.5 μg/mL Col)	4	32	16 (0.125 μg/mL Col)	2
**WSA 298**	16	8 (0.25 μg/mL Col)	2	32	8 (0.25 μg/mL Col)	4
**WSA 261**	8	4 (0.5 μg/mL Col)	2	16	8 (0.25 μg/mL Col)	2
colistin	2			1		

## Conclusions

As discussed, development of new antibiotics that overcome bacterial resistance in MDR AB is a critical need globally due to the rising rate of bacterial infections caused by this pathogen. Bacterial bioenergetics are underexplored drug targets that if properly and selectively inhibited could provide a robust area for antibiotic development, helping to slow the rate of resistance emergence. While the quinoline scaffolds derived from the antitubercular ATP synthase inhibitor BDQ have been the standard for inhibitor development of this type to date, limitations in molecular design and synthesis require new scaffolds to be explored.

Through biochemical and antibacterial evaluation of our trisubstituted pyridine library we have demonstrated that the traditional quinoline core is not required for ATP synthase inhibition in AB, opening a new area of drug development from a more readily available starting material. Specifically, we explored the SAR of AB ATP synthase inhibition and found that enzyme inhibition is driven by both molecular size and interaction with key residues in the proposed binding site of the *ac* interface of F_o_. However, despite the increase in enzyme inhibition compared to the traditional quinoline series, antibacterial activity was limited, and the most potent compounds were cytotoxic against mammalian cells indicating that there is room for improvement using strategic molecular design. Four pyridines, **WSA 276**, **WSA 288**, **WSA 289**, **WSA 298**, with variations at C2 and C4 but similar overall molecular size and flexibility demonstrated potent antibacterial activity against both susceptible and MDR AB clinical isolates due to selective inhibition of AB ATP synthase in ng/mL concentrations. Additionally, this set had the most favorable preliminary toxicity ratio when dosed in combination with colistin, making them the basis for future studies to improve activity and selectivity of the class.

## Methods

### Synthesis and Spectroscopic Data

#### General

Reagents and solvents were purchased reagent-grade and used without further purification. All reactions were performed in flame-dried glassware under an Ar or N_2_ atmosphere. Evaporation and concentration in vacuo was performed at 40–45 °C. TLC was conducted using precoated SiO_2_ 60 F254 glass plates from EMD with visualization by UV light (254 or 366 nm). NMR (^1^H or ^13^C) were recorded on an Varian INOVA-400 MHz spectrometer or a Bruker AVANCE-400 MHz spectrometer at 298 K. Residual solvent peaks were used as an internal reference (CDCl_3_ with 0.1% TMS). Coupling constants (*J*) (H,H) are given in Hz. Coupling patterns are designated as singlet (s), doublet (d), triplet (t), multiplet (m) or quintet (qu). IR spectra were recorded on a Shimadzu IRSpirit FT-IR spectrophotometer and measured neat. Low-resolution mass spectral data were acquired on a Shimadzu single quadrupole LCMS-2020. High-resolution mass spectral Samples were analyzed with a Q Exactive HF-X (ThermoFisher, Bremen, Germany) mass spectrometer. Samples were introduced via a heated electrospray source (HESI) at a flow rate of 10 μL/min. HESI source conditions were set as: nebulizer temperature 400 °C, sheath gas (nitrogen) 20 arb, auxiliary gas (nitrogen) 0 arb, sweep gas (nitrogen) 0 arb, capillary temperature 320 °C, RF voltage 45 V. The mass range was set to 100–1000 *m*/*z*. All measurements were recorded at a resolution setting of 120,000. Solutions were analyzed at 0.1 mg/mL or less based on responsiveness to the ESI mechanism. Xcalibur (ThermoFisher, Breman, Germany) was used to analyze the data. Molecular formula assignments were determined with Molecular Formula Calculator (v 1.3.0). All observed species were singly charged, as verified by unit *m*/*z* separation between mass spectral peaks corresponding to the ^12^C and ^13^C^12^C_c‑1_ isotope for each elemental composition.

Safety Statement. No chemical safety hazards were encountered during synthetic experiments of this research work.

#### General Procedure 1: Nucleophilic Aromatic Substitution

The chloropyridine carbaldehyde (**1** or **2**) (1 eq) and Na_2_S (1.6–2.2 equiv) were dissolved in *N*,*N*-dimethylformamide (0.2 M) and allowed to stir at 23 °C for 2 h. Then K_2_CO_3_ (1.5 equiv) and benzyl bromide (1.5 equiv) were added, and the solution stirred at 23 °C for an additional 2 h. The reaction was then diluted with DI H_2_O. The solution was then extracted with CH_2_CH_2_ (3×). The organic layers were then combined and concentrated under reduced pressure. Flash chromatography of the crude extracts (SiO_2_, 5 × 15 cm, 0–10% ethyl acetate/hexanes gradient elution) provided the desired product.

##### 2-(Benzylthio)­nicotinaldehyde (**3**)

2-Chloronicotinaldehyde (1.0 g, 7.1 mmol), sodium sulfide (1.3 g, 16 mmol), and benzyl bromide (1.26 mL, 10.6 mmol) were reacted using general procedure **1** to produce 2-(benzylthio)­nicotinaldehyde (**3**, 1.15 g, 71% yield) as a yellow solid. ^1^H NMR (400 MHz, CDCl_3_): δ 10.13 (s, 1H), 8.58 (d, *J* = 4.8 Hz, 1H), 7.92 (d, *J* = 7.6 Hz, 1H),, 7.40 (d, *J* = 7.3 Hz, 2H), 7.27 (t, *J* = 7.2 Hz, 2H), 7.21 (d, *J* = 7.2 Hz, 1H), 7.11 (dd, *J* = 7.6, 4.8 Hz, 1H), 4.50 (s, 2H). ^13^C NMR (CDCl_3_, 100 MHz): δ 190.05, 161.73, 153.04, 139.98, 137.50, 129.35, 128.53 (2C), 128.24 (2C), 127.24, 119.24, 34.12. HRMS (ESI): *m*/*z* [M + H]^+^ calcd for C_13_H_12_NOS, 230.0640; found, 230.0634.

##### 2-(Benzylthio)-5-bromonicotinaldehyde (**4**)

5-Bromo-2-chloronicotinaldehyde (1.0 g, 4.5 mmol), sodium sulfide (846 mg, 10.8 mmol), and benzyl bromide (0.80 mL, 6.75 mmol) were reacted using general procedure **1** to produce 2-(benzylthio)-5-bromonicotinaldehyde (**4**, 1.12 g, 80% yield) as a yellow solid. ^1^H NMR (400 MHz, CDCl_3_): δ 10.15 (s, 1H), 8.66 (d, *J* = 2.4 Hz, 1H), 8.09 (d, *J* = 2.4 Hz, 1H), 7.41 (d, *J* = 7.1 Hz, 2H), 7.27 (t, *J* = 10.3 Hz, 2H), 7.25 (m, *J* = 7.1 Hz, 1H), 4.49 (s, 2H). ^13^C NMR (CDCl_3_, 100 MHz): δ 188.55, 160.28, 153.88, 141.20, 137.03, 129.26, 129.09 (2C), 128.55 (2C), 127.38, 115.88, 34.34. HRMS (ESI): *m*/*z* [M + H]^+^ calcd for C_13_H_11_BrNOS, 307.9745; found, 307.9736.

#### General Procedure 2: Reductive Amination

The benzyl sulfide pyridine (**3** or **4**) (1 equiv) and amine (1.2 equiv) were dissolved in anhydrous methanol (0.09 M) under inert conditions. *N*,*N*-Diisopropylethylamine (3 equiv) was then added dropwise, and the reaction was allowed to stir at 23 °C for 24 h. NaBH_4_ (2 equiv) was then added. After 1 h, the reaction was diluted with DI H_2_O and extracted with dichloromethane (2×) or ethyl acetate (3×). The organic layers were then combined, dried over Na_2_SO_4_, and concentrated under reduced pressure. Flash chromatography of the crude extracts (SiO_2_, 3 × 10 cm, 0–100% CH_3_OH/CH_2_Cl_2_ gradient elution) provided the desired products.

##### WSA 264

2-(Benzylthio)­nicotinaldehyde (**3**, 200 mg, 0.92 mmol) and 2-(1-ethylpiperidin-4-yl)­ethan-1-amine (0.2 mL, 1.11 mmol) were reacted using general procedure **2** to compound **WSA 264** (137 mg, 33%) as a yellow semisolid. ^1^H NMR (400 MHz, CDCl_3_): δ 8.31 (d, *J* = 4.7 Hz, 1H), 7.45 (d, *J* = 7.4 Hz, 1H), 7.33 (d, *J* = 7.4 Hz, 2H), 7.23–7.15 (m, 3H), 6.94 (d, *J* = 2.5 Hz, 1H), 6.94 (d, *J* = 12.3 Hz, 1H), 4.42 (s, 2H), 3.66 (s, 2H), 2.89 (d, *J* = 11.5 Hz, 2H), 2.54 (t, *J* = 7.1 Hz, 2H), 2.36 (d, *J* = 7.2 Hz, 2H), 1.83 (t, *J* = 10.8 Hz, 2H), 1.59 (d, *J* = 10.7 Hz, 2H), 1.37 m, 2H), 1.27 (m,3H), 1.04 (t, *J* = 7.2 Hz, 3H). ^13^C NMR (CDCl_3_, 100 MHz): δ 156.27, 146.50, 137.09, 134.47, 131.99, 128.11 (2C), 127.43 (2C), 126.05, 118.41, 52.28, 51.53, 49.42 (2C), 45.77, 35.56, 33.20, 32.45 (2C), 30.87, 10.76. HRMS (ESI): *m*/*z* [M + H]^+^ calcd for C_22_H_32_N_3_S, 370.2317; found, 370.23077.

##### WSA 265

2-(Benzylthio)­nicotinaldehyde (**3**, 200 mg, 0.92 mmol) and 2-(1-cyclopentylpiperidin-4-yl)­ethan-1-amine (0.1 mL, 1.11 mmol) were reacted using general procedure **2** to compound **WSA 265** (108 mg, 28%) as a yellow semisolid. ^1^H NMR (400 MHz, CDCl_3_): δ 8.30 (d, *J* = 1.6 Hz, 1H), 8.29 (d, *J* = 1.6 Hz, 1H), 7.43 (dd, *J* = 7.6, 1.3 Hz, 2H), 7.32 (t, *J* = 7.2 Hz, 2H), 7.20 (t, *J* = 7.3 Hz, 1H), 6.91 (d, *J* = 7.4 Hz, 1H), 4.41 (s, 2H), 3.65 (s, 2H), 2.98 (d, *J* = 11.0 Hz, 2H), 2.52 (t, *J* = 7.0 Hz, 2H), 2.44 (m, 1H), 1.88 (t, *J* = 10.7 Hz, 2H), 1.79 (m, 2H), 1.61 (m, 4H), 1.59–1.35 (m, 9H). ^13^C NMR (CDCl_3_, 100 MHz): δ 156.26, 146.49, 137.09, 134.46, 131.97, 128.10 (2C), 127.42 (2C), 126.02,118.41, 66.76, 51.69, 49.40 (2C), 45.71, 35.46, 33.19, 32.25 (2C), 30.68 (2C), 29.14 (2C), 23.09. HRMS (ESI): *m*/*z* [M + H]^+^ calcd for C_25_H_36_N_3_S, 410.2630; found, 410.26202.

##### WSA 267

2-(Benzylthio)­nicotinaldehyde (**3**, 100 mg, 0.46 mmol) and (4-(pyrrolidin-1-ylmethyl)­phenyl)­methanamine (0.1 mL, 0.55 mmol) were reacted using general procedure **2** to compound **WSA 267** (147 mg, 79%) as a yellow semisolid. ^1^H NMR (400 MHz, CDCl_3_): δ 8.37 (dd, *J* = 6.2, 3.4 Hz, 1H), 7.56 (d, *J* = 7.36 Hz, 1H), 7.38 (t, *J* = 6.24 Hz, 3H), 7.32 (s, 1H), 7.29–7.25 (m, 4H), 7.22 (d, *J* = 7.2 Hz, 1H), 6.99 (t, *J* = 7.1 Hz, 1H), 4.49 (s, 1H), 3.76, (s, 2H) 3.75 (s, 2H) 3.74 (s, 1H), 2.70 (s, 4H), 1.85 (s, 4H). ^13^C NMR (CDCl_3_, 100 MHz): δ157.28, 147.56, 139.60, 138.17, 135.50, 135.15, 132.85, 129.55 (2C), 128.47 (2C), 128.41 (2C), 128.35 (2C), 127.08, 119.47, 59.63, 53.70 (2C), 52.96, 49.55, 34.23, 23.33 (2C). HRMS (ESI): *m*/*z* [M + H]^+^ calcd for C_25_H_30_N_3_S, 404.2160; found, 404.21552.

##### WSA 268

2-(Benzylthio)­nicotinaldehyde (**3**, 200 mg, 0.92 mmol) and 2-(4-methylpiperazin-1-yl)­ethan-1-amine (0.15 mL, 1.11 mmol) were reacted using general procedure **2** to compound **WSA 268** (286 mg, 87%) as a yellow semisolid. ^1^H NMR (400 MHz, CDCl_3_): δ 8.37 (dd, *J* = 4.8, 1.6 Hz, 1H), 7.53 (dd, *J* = 9.0, 5.9 Hz, 1H), 7.41 (dd, *J* = 8.0, 1.2 Hz, 2H), 7.28 (t, *J* = 8.4 Hz, 2H), 7.22 (t, *J* = 7.2 Hz, 1H), 6.99 (dd, *J* = 7.5, 4.9 Hz, 1H), 4.49 (s, 2H), 3.74 (s, 2H), 3.03 (s, 2H), 2.66 (t, *J* = 12.1 Hz, 2H), 2.47 (t, *J* = 12.1 Hz, 2H), 2.60–2.20 (m, 8H), 2.23 (s, 3H). ^13^C NMR (CDCl_3_, 100 MHz): δ 157.28, 147.54, 137.97, 135.56, 132.67, 129.12 (2C), 128.43 (2C), 127.06, 119.43, 57.35, 54.94 (2C), 52.50 (2C), 50.22 45.91, 45.67, 34.15. HRMS (ESI): *m*/*z* [M + H]^+^ calcd for C_20_H_29_N_4_S, 357.2113; found, 357.21024.

##### WSA 272

2-(Benzylthio)-5-bromonicotinaldehyde (**4**, 500 mg, 1.62 mmol) and 2-(1-ethylpiperidin-4-yl)­ethan-1-amine (0.36 mL, 1.94 mmol) were reacted using general procedure **2** to compound **WSA 272** (731 mg, 84%) as a pale yellow translucent semisolid. ^1^H NMR (400 MHz, CDCl_3_): δ 8.41 (d, *J* = 2.0 Hz, 1H), 7.69 (d, *J* = 1.8 Hz, 1H), 7.38 (d, *J* = 7.2 Hz, 2H), 7.29 (t, *J* = 7.3 Hz, 2H), 7.24 (t, *J* = 7.1 Hz, 1H), 4.44 (s, 2H), 3.68 (s, 2H), 3.07 (d, *J* = 10.8 Hz, 2H), 2.61 (t, *J* = 6.6 Hz, 2H), 2.55 (q, *J* = 7.3 Hz, 2H), 2.05 (t, *J* = 10.5 Hz, 2H), 1.70 (m, 2H), 1.44 (m, 5H), 1.17 (t, *J* = 7.2 Hz, 3H). ^13^C NMR (CDCl_3_, 100 MHz): δ 155.92, 148.07, 137.76, 137.51, 134.74, 129.12 (2C), 128.50 (2C), 127.21, 116.33, 52.96, 52.39, 49.72 (2C), 46.70, 36.29, 34.32, 33.03 (2C), 31.18, 11.22. HRMS (ESI): *m*/*z* [M + H]^+^ calcd for C_22_H_31_BrN_3_S, 448.1422; found, 448.14136.

##### WSA 271

2-(Benzylthio)-5-bromonicotinaldehyde (**4**, 1.0 g, 3.24 mmol) and 2-(4-methylpiperazin-1-yl)­ethan-1-amine (0.6 mL, 3.89 mmol) were reacted using general procedure **2** to compound **WSA 271** (882 mg, 63%) as a yellow semisolid . ^1^H NMR (400 MHz, CDCl_3_): δ 8.41 (d, *J* = 2 Hz, 1H) 7.71 (d, *J* = 2 Hz, 1H), 7.38 (d, *J* = 6.8 Hz, 2H), 7.28 (t, *J* = 7.2 Hz, 2H), 7.24 (m, *J* = 7.2 Hz, 1H), 4.44 (s, 2H), 3.69 (s, 2H), 2.66 (t, *J* = 6 Hz, 2H), 2.61–2.50 (m, 8H) 2.48 (t, *J* = 6 Hz, 2H), 2.25 (s, 3H). ^13^C NMR (CDCl_3_, 100 MHz): δ 155.86, 148.01, 137.66, 137.41, 134.62, 129.14 (2C), 128.49 (2C), 127.21, 116.36, 57.46, 55.03 (2C), 53.05 (2C), 49.56, 46.03, 45.78, 34.23. HRMS (ESI): *m*/*z* [M + H]^+^ calcd for C_20_H_28_BrN_4_S, 435.1218; found, 435.1210.

##### WSA 301

2-(Benzylthio)-5-bromonicotinaldehyde (**4**, 1.0 g, 3.24 mmol) and 2-(1-cyclopentylpiperidin-4-yl)­ethan-1-amine (0.83 mL, 3.89 mmol) were reacted using general procedure **2** to compound **WSA 301** (882 mg, 63%) as a yellow semisolid. ^1^H NMR (400 MHz, CDCl_3_): δ 8.40 (d, *J* = 2.0 Hz, 1H), 7.70 (d, *J* = 1.8 Hz, 1H), 7.38 (d, *J* = 7.2 Hz, 2H), 7.28 (t, *J* = 7.3 Hz, 2H), 7.23 (t, *J* = 7.1 Hz, 1H), 4.44 (s, 2H), 3.68 (s, 2H), 3.00 (d, *J* = 11.4 Hz, 2H), 2.60 (t, *J* = 7.1 Hz, 2H), 2.44 (t, *J* = 8.0 Hz, 1H), 1.88 (m, 4H), 1.66 (m, 4H), 1.53 (m, 2H), 1.40–1.29 (m, 8H). ^13^C NMR (CDCl_3_, 100 MHz): δ 155.83, 148.00, 137.77, 137.42, 134.88, 129.11 (2C), 128.48 (2C), 127.18, 116.36, 67.77, 52.85, 49.69 (2C), 46.94, 36.80, 34.30, 33.55 (2C), 32.27 (2C), 30.56 (2C), 24.22. HRMS (ESI): *m*/*z* [M + H]^+^ calcd for C_25_H_35_BrN_3_S, 488.1735; found, 488.17358.

##### WSA 293

2-(Benzylthio)-5-bromonicotinaldehyde (**4**, 1.0 g, 3.24 mmol) and (4-(pyrrolidin-1-ylmethyl)­phenyl)­methanamine (0.69 mL, 3.89 mmol) were reacted using general procedure **2** to compound **WSA 293** (998 mg, 64%) as a yellow semisolid . ^1^H NMR (400 MHz, CDCl_3_): δ 8.40 (d, *J* = 2.2 Hz, 1H), 7.73 (d, *J* = 2.1 Hz, 1H), 7.37 (d, *J* = 7.1 Hz, 2H), 7.32–7.22 (m, 7H), 4.44 (s, 2H), 3.75 (s, 2H), 3.71 (s, 2H), 3.66 (s, 2H), 2.58 (s, 4H), 1.81 (s, 4H). ^13^C NMR (CDCl_3_, 100 MHz): δ 155.89, 148.05, 138.76, 137.83, 137.51, 137.13, 134.73, 129.27 (2C), 129.13 (2C), 128.50 (2C), 128.16 (2C), 127.2, 116.36, 60.14, 54.01 (2C), 53.17, 48.92, 34.33, 23.42 (2C). HRMS (ESI): *m*/*z* [M + H]^+^ calcd for C_25_H_29_BrN_3_S, 482.1622; found, 482.12567.

#### General Procedure 3: Suzuki Coupling Reactions

To a flame dried reaction flask were sequentially added brominated pyridine intermediate (1 equiv), (ii) Cs_2_CO_3_ (2 equiv), (iii) boronic acid derivative (1.5 equiv), (iv) Pd­(PPh_3_)_4_ (0.2 equiv), as well as (v) toluene (0.1 M). The reaction solution was sparged with argon and then heated to 90 °C for 16 h. The solution was cooled to room temperature, filtered through a pad of Celite, and rinsed with DCM. Purification of the solution was carried out via flash chromatography of the crude extracts (SiO_2_, 3 × 10 cm, 0–100% CH_3_OH/CH_2_Cl_2_ gradient elution) providing the desired product.

##### WSA 277

**WSA 271** (100 mg, 0.23 mmol) and phenylboronic acid (41 mg, 0.34 mmol) were combined using general procedure **3** to produce **WSA 277** (52 mg, 35% yield) as a pale yellow semisolid. ^1^H NMR (400 MHz, CDCl_3_): δ 8.63 (d, *J* = 2.2 Hz, 1H), 7.78 (d, *J* = 2.1 Hz, 1H), 7.58 (d, *J* = 7.4 Hz, 2H), 7.45 (m, 4H), 7.38 (t, *J* = 7.3 Hz, 1H), 7.31 (t, *J* = 7.3 Hz, 2H), 7.25 (m, 1H), 4.54 (s, 2H), 3.82 (s, 2H), 2.71 (t, *J* = 6.0 Hz, 2H), 2.51 (t, *J* = 6.0 Hz, 2H), 2.50–2.20 (m, 8H), 2.25 (s, 3H). ^13^C NMR (CDCl_3_, 100 MHz): δ 156.17, 145.73, 138.02, 137.68, 134.09, 132.67, 132.62, 129.22 (2C), 129.06 (2C), 128.53 (2C), 127.78, 127.17, 126.89 (2C), 57.51, 55.05 (2C), 53.04 (2C), 50.37, 46.01, 45.89, 34.33. HRMS (ESI): *m*/*z* [M + H]^+^ calcd for C_26_H_33_N_4_S, 433.2426; found, 433.24179.

##### WSA 280

**WSA 271** (100 mg, 0.23 mmol) and 4-methylphenylboronic acid (46 mg, 0.34 mmol) were combined using general procedure **3** to produce **WSA 280** (66 mg, 65% yield) as a pale yellow solid. ^1^H NMR (400 MHz, CDCl_3_): δ 8.54 (d, *J* = 2.3 Hz, 1H), 7.68 (d, *J* = 2.2 Hz, 1H), 7.40 (d, *J* = 8.1 Hz, 2H), 7.35 (d, *J* = 7.1 Hz, 2H), 7.24–7.16 (m, 5H), 4.46 (s, 2H), 3.74 (s, 2H), 2.63 (t, *J* = 6.1 Hz, 2H), 2.43 (t, *J* = 5.9 Hz, 2H), 2.60–2.10 (m, 8H), 2.32 (s, 3H), 2.19 (s, 3H). ^13^C NMR (CDCl_3_, 100 MHz): δ 154.67, 144.59, 136.98, 136.61, 133.60, 132.99, 131.53, 131.35, 128.71 (2C), 128.13 (2C), 127.46 (2C), 126.10, 125.63 (2C), 56.26, 53.84 (2C), 51.71 (2C), 49.31, 44.76, 44.69, 33.35, 20.14. HRMS (ESI): *m*/*z* [M + H]^+^ calcd for C_27_H_35_N_4_S, 447.2582; found, 447.2572.

##### WSA 278

**WSA 271** (100 mg, 0.23 mmol) and (4-methoxyphenyl)­boronic acid (52 mg, 0.34 mmol) were combined using general procedure **3** to produce **WSA 278** (39 mg, 25% yield) as a pale yellow solid. ^1^H NMR (400 MHz, CDCl_3_): δ 8.59 (d, *J* = 2.3 Hz, 1H), 7.73 (d, *J* = 2.3 Hz, 1H), 7.52 (d, *J* = 8.7 Hz, 2H), 7.43 (d, *J* = 7.1 Hz, 2H), 7.31 (t, *J* = 7.3 Hz, 2H), 7.25 (d, *J* = 7.2 Hz, 1H), 7.00 (d, *J* = 8.7 Hz, 2H), 4.53 (s, 2H), 3.86 (s, 3H), 3.81 (s, 2H), 2.71 (t, *J* = 6.0 Hz, 2H), 2.51 (t, *J* = 6.0 Hz, 2H), 2.70–2.20 (m, 8H), 2.26 (s, 3H). ^13^C NMR (CDCl_3_, 100 MHz): δ 159.48, 155.33, 145.42, 138.07, 133.80, 132.57, 132.33, 130.06, 129.20 (2C), 128.52 (2C), 127.95 (2C), 127.15, 114.49 (2C), 57.46, 55.41, 55.01 (2C), 52.97 (2C), 50.42, 45.96, 45.85, 34.35. HRMS (ESI): *m*/*z* [M + H]^+^ calcd for C_27_H_35_N_4_OS, 463.2532; found, 463.25204.

##### WSA 273

**WSA 271** (50 mg, 0.11 mmol) and 4-fluorophenylboronic acid (24 mg, 0.17 mmol) were combined using general procedure **3** to produce **WSA 273** (38 mg, 75% yield) as a pale yellow solid. ^1^H NMR (400 MHz, CDCl_3_): δ^1^ 8.57 (d, *J* = 2.3 Hz, 1H), 7.74 (d, *J* = 2.2 Hz, 1H), 7.54 (d, *J* = 8.7 Hz, 1H), 7.53 (d, *J* = 8.7 Hz, 1H), 7.43 (d, *J* = 7.2 Hz, 2H), 7.31 (t, *J* = 7.0 Hz, 2H), 7.27 (m, 1H), 7.15 (t, *J* = 8.7 Hz, 2H), 4.54 (s, 2H), 3.82 (s, 2H), 2.72 (t, *J* = 11.9 Hz, 2H), 2.52 (t, *J* = 11.9 Hz, 2H), 2.60–2.20 (m, 8H), 2.27 (s, 3H). ^13^C NMR (CDCl_3_, 100 MHz): δ 162.68 (d, *J*_CF_ = 247.3 Hz), 156.18, 145.57, 137.97, 134.00, 133.77, 133.74, 132.15 (d, *J*_CF_ = 85.4 Hz, 2C), 129.19 (2C), 128.54 (2C), 128.46, 127.20, 116.01 (d, *J*_CF_ = 21.7 Hz, 2C), 57.36, 54.94 (2C), 52.86 (2C), 50.29, 45.89, 45.84, 34.29. HRMS (ESI): *m*/*z* [M + H]^+^ calcd for C_26_H_32_FN_4_S, 451.2332; found, 451.23279.

##### WSA 274

**WSA 271** (100 mg, 0.23 mmol) and 2,4-difluorophenylboronic acid (54 mg, 0.34 mmol) were combined using general procedure **3** to produce **WSA 274** (46 mg, 43% yield) as a pale yellow solid. . ^1^H NMR (400 MHz, CDCl_3_): δ 8.43 (d, *J* = 3.4 Hz, 1H), 7.63 (s, 1H), 7.35 (d, *J* = 7.1 Hz, 2H), 7.31 (m, 1H), 7.22–7.10 (m, 3H), 6.87 (m, 2H), 4.46 (s, 2H), 3.73 (s, 2H), 2.63 (t, *J* = 6.0 Hz, 2H), 2.42 (t, *J* = 6.0 Hz, 2H), 2.56–2.20 (m, 8H), 2.17 (s, 3H). ^13^C NMR (CDCl_3_, 100 MHz): δ 161.50 (dd, *J*_CF_ = 248.4, 11.85 Hz), 158.82 (dd, *J*_CF_ = 249.2, 11.7 Hz, 1C), 155.76, 145.91 (d, *J*_CF_ = 3.5 Hz), 136.84, 134.48 (d, *J*_CF_ = 2.8 Hz), 131.40, 130.01 (dd, *J*_CF_ = 9.4, 4.8 Hz), 128.15 (2C), 127.47 (2C), 126.13, 125.59, 120.79 (dd, *J*_CF_ = 17.9, 9.9 Hz), 110.88 (q, *J* = 8.3, 3.7 Hz), 103.57 (t, *J* = 25.8 Hz), 56.41, 53.96 (2C), 51.94 (2C), 49.10, 44.92, 44.76, 33.17. HRMS (ESI): *m*/*z* [M + H]^+^ calcd for C_26_H_31_F_2_N_4_S, 469.2237; found, 469.22275.

##### WSA 275

**WSA 271** (118 mg, 0.27 mmol) and 3-fluoro-4-methoxyphenylboronic acid (68 mg, 0.40 mmol) were combined using general procedure **3** to produce **WSA 275** (69 mg, 53% yield) as an off white solid. ^1^H NMR (400 MHz, CDCl_3_): δ 8.49 (d, *J* = 2.3 Hz, 1H), 7.64 (d, *J* = 2.3 Hz, 1H), 7.35 (d, *J* = 7.1 Hz, 2H), 7.27–7.14 (m, 5H), 6.96 (t, *J* = 8.8 Hz, 1H), 4.45 (s, 2H), 3.85 (s, 3H), 3.73 (s, 2H), 2.64 (t, *J* = 12.0 Hz, 2H), 2.44 (t, *J* = 12.0 Hz, 2H), 2.65–2.14 (m, 8H), 2.19 (s, 3H). ^13^C NMR (CDCl_3_, 100 MHz): δ 154.98, 151.58 (d, *J*_CF_ = 246.1 Hz), 146.34 (d, *J*_CF_ = 10.7 Hz), 144.23, 136.92, 132.54, 131.61, 130.17 (d, *J*_CF_ = 1.4 Hz), 129.66 (d, *J*_CF_ = 6.6 Hz), 128.13 (2C), 127.47 (2C), 126.12, 121.45 (d, *J*_CF_ = 3.4 Hz), 113.43 (d, *J*_CF_ = 19.0 Hz), 112.71 (d, *J* = 2.1 Hz), 56.37, 55.27, 53.93 (2C), 51.88 (2C), 49.23, 44.87, 44.82, 33.23. HRMS (ESI): *m*/*z* [M + H]^+^ calcd for C_27_H_34_FN_4_OS, 481.2437; found, 481.24362.

##### WSA 276

**WSA 271** (100 mg, 0.23 mmol) and 4-(dimethylamino)­phenylboronic acid (54 mg, 0.34 mmol) were combined using general procedure **3** to produce **WSA 276** (52 mg, 47% yield) as a pale yellow semisolid. ^1^H NMR (400 MHz, CDCl_3_): δ 8.52 (d, *J* = 2.2 Hz, 1H), 7.64 (d, *J* = 2.1 Hz, 1H), 7.41 (d, *J* = 8.8 Hz, 2H), 7.35 (d, *J* = 7.2 Hz, 2H), 7.22 (t, *J* = 7.3 Hz, 2H), 7.16 (t, *J* = 7.2 Hz, 1H), 6.72 (d, *J* = 8.8 Hz, 2H), 4.45 (s, 2H), 3.72 (s, 2H), 2.92 (s, 6H), 2.62 (t, *J* = 6.0 Hz, 2H), 2.42 (t, *J* = 6.0 Hz, 2H), 2.46–2.13 (m, 8H), 2.17 (s, 3H). ^13^C NMR (CDCl_3_, 100 MHz): δ 153.26, 149.13, 144.03, 137.09, 132.30, 131.69, 131.47, 128.13 (2C), 127.43 (2C), 126.39 (2C), 126.03, 124.16, 111.75 (2C), 56.46, 54.00 (2C), 51.99 (2C), 49.48, 44.97, 44.77, 39.46 (2C), 33.35. HRMS (ESI): *m*/*z* [M + H]^+^ calcd for C_28_H_38_N_5_S, 476.2848; found, 476.28409.

##### WSA 302

**WSA 301** (100 mg, 0.20 mmol) and phenylboronic acid (37 mg, 0.30 mmol) were combined using general procedure **3** to produce **WSA 302** (14 mg, 13% yield) as a pale yellow solid. ^1^H NMR (400 MHz, CDCl_3_): δ 8.62 (d, *J* = 1.8 Hz, 1H), 7.77 (s, 1H), 7.58 (d, *J* = 7.4 Hz, 2H), 7.45 (m, 4H), 7.38 (t, *J* = 7.2 Hz, 1H), 7.30 (t, *J* = 7.3 Hz, 2H), 7.24 (m, 1H), 4.54 (s, 2H), 3.80 (s, 2H), 3.00 (d, *J* = 11.1 Hz, 2H), 2.64 (t, *J* = 7.1 Hz, 2H), 2.44 (t, *J* = 8.5, 1H), 1.88 (m, 4H), 1.66 (m, 4H), 1.53–1.32 (m, 10H). ^13^C NMR (CDCl_3_, 100 MHz): δ 156.10, 145.68, 138.12, 137.70, 133.99, 132.92, 132.62, 129.17 (2C), 129.04 (2C), 128.50 (2C), 127.75, 127.12, 126.85 (2C), 67.79, 52.84, 50.43 (2C), 46.96, 36.77, 34.39, 33.54 (2C), 32.21 (2C), 30.50 (2C), 24.20. HRMS (ESI): *m*/*z* [M + H]^+^ calcd for C_31_H_40_N_3_S, 486.2943; found, 486.29349.

##### WSA 288

**WSA 301** (75 mg, 0.15 mmol) and (4-methoxyphenyl)­boronic acid (35 mg, 0.23 mmol) were combined using general procedure **3** to produce **WSA 288** (54 mg, 69% yield) as a opaque solid. ^1^H NMR (400 MHz, CDCl_3_): δ 8.59 (s, 1H), 7.71 (s, 1H), 7.51 (d, *J* = 8.5 Hz, 2H), 7.42 (d, *J* = 7.3 Hz, 2H), 7.29 (t, *J* = 7.4 Hz, 2H), 7.23 (t, *J* = 7.1 Hz, 1H), 6.99 (d, *J* = 8.5 Hz, 2H), 4.53 (s, 2H), 3.84 (s, 3H), 3.78 (s, 2H), 3.06 (d, *J* = 10.2 Hz, 2H), 2.63 (t, *J* = 6.6 Hz, 2H), 2.52 (s, 1H), 1.95 (m, 2H), 1.86 (m, 2H), 1.68 (m, 4H), 1.52–1.25 (m, 9H). ^13^C NMR (CDCl_3_, 100 MHz): δ 159.52, 155.29, 145.35, 138.20, 133.67, 132.85, 132.31, 130.08, 129.15 (2C), 128.48 (2C), 127.91 (2C), 127.09, 114.51 (2C), 67.83, 55.38, 52.72, 50.49 (2C), 46.82, 36.54, 34.41, 33.27 (2C), 31.71 (2C), 30.15 (2C), 24.12. HRMS (ESI): *m*/*z* [M + H]^+^ calcd for C_32_H_42_N_3_OS, 516.3049; found, 516.30414.

##### WSA 289

**WSA 301** (75 mg, 0.15 mmol) and 4-methylphenylboronic acid (31 mg, 0.23 mmol) were combined using general procedure **3** to produce **WSA 289** (48 mg, 63% yield) as a opaque solid. ^1^H NMR (400 MHz, CDCl_3_): δ 8.61 (d, *J* = 1.7 Hz, 1H), 7.74 (d, *J* = 1.3 Hz, 1H), 7.47 (d, *J* = 7.9 Hz, 2H), 7.42 (d, *J* = 7.3 Hz, 2H), 7.27 (m, 6H), 4.53 (s, 2H), 3.79 (s, 2H), 3.04 (d, *J* = 10.7 Hz, 2H), 2.63 (t, *J* = 6.6 Hz, 2H), 2.49 (s, 1H), 2.39 (s, 3H), 2.00–1.82 (m, 4H), 1.68 (m, 5H), 1.511–1.30 (m, 9H). ^13^C NMR (CDCl_3_, 100 MHz): δ 155.71, 145.55, 138.18, 137.63, 134.74, 133.86, 132.85, 132.56, 129.77, 129.17 (2C), 128.49 (2C), 128.44 (2C), 127.10, 126.67 (2C), 67.82, 52.75, 50.48 (2C), 46.86, 36.60, 34.40, 33.33 (2C), 31.83 (2C), 30.24 (2C), 24.15, 21.17. HRMS (ESI): *m*/*z* [M + H]^+^ calcd for C_32_H_42_N_3_S, 500.3099; found, 500.30881.

##### WSA 290

**WSA 301** (75 mg, 0.15 mmol) and 3-fluoro-4-methoxyphenylboronic acid (34 mg, 0.23 mmol) were combined using general procedure **3** to produce **WSA 290** (43 mg, 53% yield) as a opaque solid. ^1^H NMR (400 MHz, CDCl_3_): δ 8.56 (s, 1H), 7.70 (s, 1H), 7.42 (d, *J* = 7.3 Hz, 2H), 7.32–7.21 (m, 5H), 7.04 (t, *J* = 8.6 Hz, 1H), 4.52 (s, 2H), 3.93 (s, 3H), 3.78 (s, 2H), 3.10 (d, *J* = 9.6 Hz, 2H), 2.64 (m, 2H), 2.58 (s, 1H), 2.01 (m, 2H), 1.87 (m, 2H), 1.71–1.43 (m, 14H). ^13^C NMR (CDCl_3_, 100 MHz): δ 156.05, 152.68 (d, *J*_CF_ = 246.3 Hz), 147.43 (d, *J*_CF_ = 10.6 Hz), 145.23, 138.10, 133.48, 132.94, 131.21, 130.76 (d, *J*_CF_ = 6.5 Hz), 129.15 (2C), 128.49 (2C), 127.12, 122.48 (d, *J*_CF_ = 3.3 Hz), 114.45 (d, *J*_CF_ = 19.1 Hz), 113.89 (d, *J*_CF_ = 2.0 Hz), 67.84, 56.36, 52.65, 50.36 (2C), 46.80, 36.43, 34.37, 33.13 (2C), 31.43 (2C), 29.96 (2C), 24.08. HRMS (ESI): *m*/*z* [M + H]^+^ calcd for C_32_H_41_FN_3_OS, 534.2954; found, 534.29443.

##### WSA 291

**WSA 301** (75 mg, 0.15 mmol) and 4-fluorophenylboronic acid (32 mg, 0.23 mmol) were combined using general procedure **3** to produce **WSA 291** (48 mg, 62% yield) as a pale yellow solid. ^1^H NMR (400 MHz, CDCl_3_): δ 8.57 (d, *J* = 2.0 Hz, 1H), 7.71 (d, *J* = 1.8 Hz, 1H), 7.53 (d, *J* = 13.9 Hz, 2H), 7.53 (d, *J* = 3.2 Hz, 2H), 7.42 (d, *J* = 7.2 Hz, 2H), 7.31–7.23 (m, 3H), 7.24 (d, *J* = 7.2 Hz, 1H), 7.14 (t, *J* = 8.6 Hz, 2H), 4.53 (s, 2H), 3.79 (s, 2H), 3.08 (d, *J* = 10.3 Hz, 2H), 2.64 (t, *J* = 6.8 Hz, 2H), 2.54 (s, 1H), 1.97 (s, 2H), 1.87 (s, 2H), 1.70–1.40 (m, 14H). ^13^C NMR (CDCl_3_, 100 MHz): δ 162.69 (d, *J*_CF_ = 247.3 Hz), 156.18, 145.49, 138.09, 133.83, 133.79, 132.94, 131.69 (2C), 129.16 (2C), 128.50 (2C), 128.43, 127.13, 116.00 (d, *J*_CF_ = 21.7 Hz, 2C), 67.84, 52.71, 50.39 (2C), 46.87, 36.52, 34.37, 33.25 (2C), 31.66 (2C), 30.12 (2C), 24.12. HRMS (ESI): *m*/*z* [M + H]^+^ calcd for C_31_H_39_FN_3_S, 504.2849; found, 504.28461.

##### WSA 292

**WSA 301** (75 mg, 0.15 mmol) and 2,4-difluorophenylboronic acid (34 mg, 0.23 mmol) were combined using general procedure **3** to produce **WSA 292** (37 mg, 46% yield) as a pale yellow solid. ^1^H NMR (400 MHz, CDCl_3_): δ 8.51 (s, 1H), 7.69 (s, 1H), 7.43 (d, *J* = 7.3 Hz, 2H), 7.39 (q, *J* = 5.0 Hz, 2H), 7.30 (t, *J* = 7.3 Hz, 1H), 7.25 (m, 3H), 6.95 (m, 2H), 4.53 (s, 2H), 3.79 (s, 2H), 3.12 (d, *J* = 10.1 Hz, 2H), 2.64 (m, 3H), 2.03 (s, 2H), 1.88 (m, 2H), 1.70 (m, 4H), 1.58–1.35 (m, 10H). ^13^C NMR (CDCl_3_, 100 MHz): δ 162.60 (dd, *J*_CF_ = 248.3, 11.5 Hz), 159.89 (dd, *J*_CF_ = 249.3, 12.0 Hz), 156.95, 147.00 (d, *J*_CF_ = 3.4 Hz), 138.02, 135.64, 132.51, 131.04 (q, *J*_CF_ = 4.7 Hz), 129.20 (2C), 128.50 (2C), 127.15, 126.67, 121.77 (d, *J*_CF_ = 17.7 Hz), 111.98 (dd, *J*_CF_ = 21.0, 3.6 Hz), 104.63 (t, *J*_CF_ = 25.9 Hz), 67.97, 52.34, 50.34 (2C), 46.36, 35.69, 34.34, 32.29, 29.88 (2C), 29.70 (2C), 28.90 (2C), 23.83. HRMS (ESI): *m*/*z* [M + H]^+^ calcd for C_31_H_38_F_2_N_3_S, 522.2755; found, 522.27478.

##### WSA 303

**WSA 301** (100 mg, 0.20 mmol) and 4-(dimethylamino)­phenylboronic acid (50 mg, 0.30 mmol) were combined using general procedure **3** to produce **WSA 303** (28 mg, 26% yield) as a light yellow solid. ^1^H NMR (400 MHz, CDCl_3_): δ 8.60 (d, *J* = 1.9 Hz, 1H), 7.70 (d, *J* = 1.7 Hz, 1H), 7.49 (d, *J* = 8.7 Hz, 2H), 7.42 (d, *J* = 7.3 Hz, 2H), 7.30 (t, *J* = 7.4 Hz, 2H), 7.24 (d, *J* = 7.3 Hz, 1H), 6.81 (d, *J* = 8.7 Hz, 2H), 4.52 (s, 2H), 3.78 (s, 2H), 3.00 (m, 8H), 2.63 (m, 2H), 2.48 (s, 1H), 1.86 (m, 4H), 1.66 (m, 4H), 1.53 (q, *J* = 5.0 Hz, 1H), 1.44–1.36 (m, 10H). ^13^C NMR (CDCl_3_, 100 MHz): δ 154.27, 150.23, 145.08, 138.29, 133.31, 132.79 (2C), 129.15 (2C), 128.47 (2C), 127.43 (2C), 127.06, 125.28, 112.83 (2C), 67.81, 52.76, 50.61 (2C), 46.80, 40.49 (2C), 36.63, 34.50, 33.38 (2C), 31.92 (2C), 30.29 (2C), 24.16. HRMS (ESI): *m*/*z* [M + H]^+^ calcd for C_33_H_45_N_4_S, 529.3365; found, 529.33673.

##### WSA 294

**WSA 293** (75 mg, 0.16 mmol) and phenylboronic acid (28 mg, 0.23 mmol) were combined using general procedure **3** to produce **WSA 294** (54 mg, 73% yield) as a clear semisolid. ^1^H NMR (400 MHz, CDCl_3_): δ 8.62 (d, *J* = 1.5 Hz, 1H), 7.79 (s, 1H), 7.57 (d, *J* = 7.4 Hz, 2H), 7.43 (m, 4H), 7.35 (d, *J* = 8.6 Hz, 2H), 7.31–7.29 (m, 5H), 7.26 (d, *J* = 11.4 Hz, 1H), 4.54 (s, 2H), 3.82 (s, 2H), 3.79 (s, 2H), 3.69 (s, 2H), 2.62 (s, 4H), 1.82 (s, 4H). ^13^C NMR (CDCl_3_, 100 MHz): δ 156.15, 145.71, 139.26, 138.18, 137.68, 136.28, 134.04, 132.76, 132.59, 129.38 (2C), 129.17 (2C), 129.07 (2C), 128.50 (2C), 128.28 (2C), 127.77, 127.12, 126.85 (2C), 59.96, 53.88, 53.15 (2C), 49.64, 34.40, 23.39 (2C). HRMS (ESI): *m*/*z* [M + H]^+^ calcd for C_31_H_34_N_3_S, 480.2473; found, 480.24698.

##### WSA 295

**WSA 293** (75 mg, 0.16 mmol) and 4-methylphenylboronic acid (31 mg, 0.23 mmol) were combined using general procedure **3** to produce **WSA 295** (75 mg, 98% yield) as a clear semisolid. ^1^H NMR (400 MHz, CDCl_3_): δ 8.61 (d, *J* = 1.7 Hz, 1H), 7.76 (d, *J* = 1.4 Hz, 1H), 7.46 (d, *J* = 7.6 Hz, 2H), 7.44 (d, *J* = 7.6 Hz, 2H), 7.33–7.19 (m, 9H), 4.53 (s, 2H), 3.81 (s, 2H), 3.78 (s, 2H), 3.67 (s, 2H), 2.59 (s, 4H), 2.39 (s, 3H), 1.81 (s, 4H). ^13^C NMR (CDCl_3_, 100 MHz): δ 155.74, 145.59, 139.15, 138.23, 137.65, 136.64, 134.75, 133.90, 132.73, 132.55, 129.80 (2C), 129.31 (2C), 129.18 (2C), 128.50 (2C), 128.25 (2C), 127.11, 126.69 (2C), 60.06, 53.95 (2C), 53.15, 49.68, 34.41, 23.41 (2C), 21.20. HRMS (ESI): *m*/*z* [M + H]^+^ calcd for C_32_H_36_N_3_S, 494.2630; found, 494.2627.

##### WSA 296

**WSA 293** (75 mg, 0.16 mmol) and 4-methoxyphenylboronic acid (35 mg, 0.23 mmol) were combined using general procedure **3** to produce **WSA 296** (66 mg, 84% yield) as a clear semisolid. ^1^H NMR (400 MHz, CDCl_3_): δ 8.58 (d, *J* = 1.8 Hz, 1H), 7.74 (s, 1H), 7.50 (d, *J* = 8.6 Hz, 2H), 7.41 (d, *J* = 7.3 Hz, 2H), 7.30–7.23 (m, 6H), 7.22 (m, 1H), 6.98 (d, *J* = 8.6 Hz, 2H), 4.52 (s, 2H), 3.84 (s, 3H), 3.81 (s, 2H), 3.78 (s, 2H), 3.69 (s, 2H), 2.61 (s, 4H), 1.82 (s, 4H). ^13^C NMR (CDCl_3_, 100 MHz): δ 159.53, 155.30, 145.37, 139.25, 138.26, 136.36, 133.69, 132.73, 132.29, 130.09 (2C), 129.34 (2C), 129.16 (2C), 128.49 (2C), 128.27 (2C), 127.91, 127.09, 114.55 (2C), 59.98, 55.40, 53.90 (2C), 53.14, 49.69, 34.42, 23.40 (2C). HRMS (ESI): *m*/*z* [M + H]^+^ calcd for C_32_H_36_N_3_OS, 510.2579; found, 510.25714.

##### WSA 297

**WSA 293** (75 mg, 0.16 mmol) and 3-fluoro-4-methoxyphenylboronic acid (39 mg, 0.23 mmol) were combined using general procedure **3** to produce **WSA 297** (64 mg, 79% yield) as a clear semisolid. ^1^H NMR (400 MHz, CDCl_3_): δ 8.55 (d, *J* = 1.9 Hz, 1H), 7.71 (d, *J* = 1.7 Hz, 1H), 7.41 (d, *J* = 7.3 Hz, 2H), 7.34–7.20 (m, 9H), (t, *J* = 8.7 Hz, 1H), 4.52 (s, 2H), 3.92 (s, 3H), 3.81 (s, 2H), 3.79 (s, 2H), 3.69 (s, 2H), 2.61 (s, 4H), 1.82 (s, 4H). ^13^C NMR (CDCl_3_, 100 MHz): δ 156.04, 152.71 (d, *J*_CF_ = 246.3 Hz), 147.44 (d, *J*_CF_ = 10.7 Hz), 145.24, 139.15, 138.15, 136.53, 133.51, 132.83, 131.19, 130.79 (d, *J*_CF_ = 6.6 Hz), 129.34 (2C), 129.15 (2C), 128.49 (2C), 128.25 (2C), 127.12, 122.48 (d, *J*_CF_ = 3.5 Hz), 114.46 (d, *J*_CF_ = 19.2 Hz), 113.93 (d, *J* = 2.0 Hz), 60.00, 56.38, 53.91 (2C), 53.18, 49.55, 34.37, 23.40 (2C). HRMS (ESI): *m*/*z* [M + H]^+^ calcd for C_32_H_35_FN_3_OS, 528.2485; found, 528.24847.

##### WSA 298

**WSA 293** (75 mg, 0.16 mmol) and 4-fluorophenylboronic acid (32 mg, 0.23 mmol) were combined using general procedure **3** to produce **WSA 298** (62 mg, 81% yield) as a clear semisolid. ^1^H NMR (400 MHz, CDCl_3_): δ 8.56 (d, *J* = 2.0 Hz, 1H), 7.74 (d, *J* = 1.7 Hz, 1H), 7.51 (d, *J* = 13.9 Hz, 2H), 7.51 (d, *J* = 3.2 Hz, 2H), 7.42–7.20 (m, 7H), 7.13 (t, *J* = 8.6 Hz, 2H), 4.52 (s, 2H), 3.81 (s, 2H), 3.79 (s, 2H), 3.68 (s, 2H), 2.61 (s, 4H), 1.82 (s, 4H). ^13^C NMR (CDCl_3_, 100 MHz): δ 162.70 (d, *J*_CF_ = 247.2 Hz), 156.18, 145.50, 139.16, 138.15, 136.44, 133.83, 133.80, 132.82, 131.66 (2C), 129.36 (2C), 129.16 (2C), 128.51 (2C), 128.44, 128.26 (2C), 127.14, 116.01 (d, *J*_CF_ = 21.5 Hz, 2C), 59.99, 53.92 (2C), 53.20, 49.56, 34.37, 23.39 (2C). HRMS (ESI): *m*/*z* [M + H]^+^ calcd for C_31_H_33_FN_3_S, 498.2379; found, 498.23721.

##### WSA 299

**WSA 293** (75 mg, 0.16 mmol) and 2,4-difluorophenylboronic acid (36 mg, 0.23 mmol) were combined using general procedure **3** to produce **WSA 299** (49 mg, 62% yield) as a clear semisolid. ^1^H NMR (400 MHz, CDCl_3_): δ 8.51 (s, 1H), 7.73 (s, 1H), 7.42 (d, *J* = 7.2 Hz, 2H), 7.39–7.21 (m, 8H), 6.95 (m, 2H), 4.53 (s, 2H), 3.81 (s, 2H), 3.79 (s, 2H), 3.73 (s, 2H), 2.67 (s, 4H), 1.85 (s, 4H). ^13^C NMR (CDCl_3_, 100 MHz): δ 162.60 (dd, *J*_CF_ = 249.0, 12.0 Hz), 159.93 (dd, *J*_CF_ = 249.0, 11.0 Hz, 156.82, 146.95 (d, *J*_CF_ = 3.4 Hz), 139.40, 138.05, 135.74, 135.50 (d, *J*_CF_ = 2.8 Hz), 132.53, 131.05 (dd, *J*_CF_ = 4.8 Hz), 129.45 (2C), 129.17 (2C), 128.50 (2C), 128.32 (2C), 127.14, 126.65, 121.87 (q, *J*_CF_ = 5.9 Hz), 112.05 (d, *J*_CF_ = 3.7 Hz), 111.84 (d, *J*_CF_ = 3.8 Hz), 104.64 (t, *J*_CF_ = 25.8 Hz), 59.82, 53.81 (2C), 53.06, 49.40, 34.30, 23.36 (2C). HRMS (ESI): *m*/*z* [M + H]^+^ calcd for C_31_H_32_F_2_N_3_S, 516.2285; found, 516.22791.

##### WSA 304

**WSA 272** (60 mg, 0.13 mmol) and phenylboronic acid (25 mg, 0.2 mmol) were combined using general procedure **3** to produce **WSA 304** (62 mg, 69% yield) as a clear semisolid. ^1^H NMR (400 MHz, CDCl_3_): δ 8.63 (d, *J* = 2.1 Hz, 1H), 7.76 (d, *J* = 2.0 Hz, 1H), 7.58 (d, *J* = 7.4 Hz, 2H), 7.44 (m, 4H), 7.38 (t, *J* = 7.1 Hz, 1H), 7.29 (t, *J* = 7.6 Hz, 2H), 7.23 (m, 1H), 4.54 (s, 2H), 3.80 (s, 2H), 2.99 (d, *J* = 11.3 Hz, 2H), 2.64 (t, *J* = 7.0 Hz, 2H), 2.46 (q, *J* = 7.2 Hz, 2H), 1.94 (t, *J* = 10.7 Hz, 2H), 1.68 (d, *J* = 10.2 Hz, 2H), 1.47–1.30 (m, 5H), 1.13 (t, *J* = 7.2 Hz, 3H). ^13^C NMR (CDCl_3_, 100 MHz): δ 156.14, 145.71, 138.13, 137.65, 134.06, 132.85, 132.62, 129.17 (2C), 129.05 (2C), 128.50 (2C), 127.77, 127.13, 126.48 (2C), 53.18, 52.50, 50.45 (2C), 46.82, 36.49, 34.40, 33.31 (2C), 31.64, 11.56. HRMS (ESI): *m*/*z* [M + H]^+^ calcd for C_28_H_36_N_3_S, 446.2630; found, 446.26227.

##### WSA 300

**WSA 272** (75 mg, 0.17 mmol) and 4-methylphenylboronic acid (34 mg, 0.25 mmol) were combined using general procedure **3** to produce **WSA 300** (25 mg, 34% yield) as a pale yellow solid. ^1^H NMR (400 MHz, CDCl_3_): δ 8.61 (s, 1H), 7.74 (s, 1H), 7.48 (d, *J* = 7.6 Hz, 2H), 7.42 (d, *J* = 7.1 Hz, 2H), 7.27 (m, 5H), 4.53 (s, 2H), 3.79 (s, 2H), 2.93 (d, *J* = 10.5 Hz, 2H), 2.64 (t, *J* = 6.8 Hz, 2H), 2.40 (m, 5H), 1.87 (t, *J* = 10.5 Hz, 2H), 1.66 (d, *J* = 11.2 Hz, 2H), 1.45 (d, *J* = 6.1 Hz, 2H), 1.32 (m, 3H), 1.09 (t, *J* = 6.9 Hz, 3H). ^13^C NMR (CDCl_3_, 100 MHz): δ 155.70, 145.56, 138.17, 137.65, 134.75, 133.87, 132.86, 132.58, 129.76 (2C), 129.16 (2C), 128.49 (2C), 127.11, 126.68 (2C), 53.40, 52.62, 50.49 (2C), 46.93, 36.73, 34.40, 33.59 (2C), 32.13, 21.16, 11.95. HRMS (ESI): *m*/*z* [M + H]^+^ calcd for C_29_H_38_N_3_S, 460.2786; found, 460.27847.

##### WSA 305

**WSA 272** (60 mg, 0.13 mmol) and 4-methoxyphenylboronic acid (30.5 mg, 0.2 mmol) were combined using general procedure **3** to produce **WSA 305** (23 mg, 25% yield) as a clear semisolid. ^1^H NMR (400 MHz, CDCl_3_): δ 8.59 (d, *J* = 2.1 Hz, 1H), 7.72 (d, *J* = 2.0 Hz, 1H), 7.51 (d, *J* = 8.7 Hz, 2H), 7.42 (d, *J* = 7.2 Hz, 2H), 7.30 (t, *J* = 7.4 Hz, 2H), 7.24 (d, *J* = 7.3 Hz, 1H), 6.99 (d, *J* = 8.6 Hz, 2H), 4.53 (s, 2H), 3.85 (s, 3H), 3.79 (s, 2H), 2.92 (d, *J* = 11.3 Hz, 2H), 2.64 (t, *J* = 7.2 Hz, 2H), 2.39 (q, *J* = 7.2 Hz, 2H), 1.85 (t, *J* = 11.0 Hz, 2H), 1.66 (d, *J* = 11.7 Hz, 2H), 1.45 (d, *J* = 6.9 Hz, 2H), 1.29 (m, 3H), 1.08 (t, *J* = 7.2 Hz, 3H). ^13^C NMR (CDCl_3_, 100 MHz): δ 159.51, 155.26, 145.35, 138.36, 133.67, 132.87, 132.33, 130.11, 129.15 (2C), 128.49 (2C), 127.92 (2C), 127.10, 114.51 (2C), 55.39, 53.44, 52.63, 50.50 (2C), 46.96, 36.77, 34.41, 33.64 (2C), 32.22, 12.01. HRMS (ESI): *m*/*z* [M + H]^+^ calcd for C_29_H_38_N_3_OS, 476.2736; found, 476.27271.

##### WSA 306

**WSA 272** (60 mg, 0.13 mmol) and 3-fluoro-4-methoxyphenylboronic acid (34 mg, 0.2 mmol) were combined using general procedure 3 to produce **WSA 306** (37 mg, 37% yield) as a clear semisolid. ^1^H NMR (400 MHz, CDCl_3_): δ 8.56 (d, *J* = 2.0 Hz, 1H), 7.70 (d, *J* = 1.8 Hz, 1H), 7.42 (d, *J* = 7.2 Hz, 2H), 7.30 (m, 4H), 7.24 (t, *J* = 7.2 Hz, 1H), 7.04 (t, *J* = 8.7 Hz, 1H), 4.53 (s, 2H), 3.93 (s, 3H), 3.79 (s, 2H), 2.97 (d, *J* = 11.1 Hz, 2H), 2.65 (t, *J* = 7.0 Hz, 2H), 2.44 (q, *J* = 7.1 Hz, 2H), 1.91 (t, *J* = 10.6 Hz, 2H), 1.68 (d, *J* = 10.9 Hz, 2H), 1.46–1.30 (m, 5H), 1.11 (t, *J* = 7.2 Hz, 3H). ^13^C NMR (CDCl_3_, 100 MHz): δ 156.02, 152.70 (d, *J*_CF_ = 246.3 Hz), 147.44 (d, *J* = 10.7 Hz), 145.24, 138.10, 133.48, 132.97, 131.22 (d, *J*_CF_ = 1.4 Hz), 130.80 (d, *J*_CF_ = 6.6 Hz), 129.15 (2C), 128.50 (2C), 127.13, 122.47 (d, *J*_CF_ = 3.5 Hz), 114.48 (d, *J*_CF_ = 19.0 Hz), 113.90 (d, *J*_CF_ = 2.1 Hz), 56.37, 53.31, 52.58, 50.37 (2C), 46.94, 36.64, 34.36, 33.48 (2C), 31.93, 11.78. HRMS (ESI): *m*/*z* [M + H]^+^ calcd for C_29_H_37_FN_3_OS, 494.2641; found, 494.26373.

##### WSA 307

**WSA 272** (60 mg, 0.13 mmol) and 4-fluorophenylboronic acid (28.1 mg, 0.2 mmol) were combined using general procedure **3** to produce **WSA 307** (25 mg, 27% yield) as a clear semisolid. ^1^H NMR (400 MHz, CDCl_3_): δ 8.57 (d, *J* = 1.8 Hz, 1H), 7.72 (s, 1H), 7.53 (q, *J* = 4.6 Hz, 2H), 7.43 (d, *J* = 7.2 Hz, 2H), 7.31 (t, *J* = 7.3 Hz, 2H), 7.24 (s, 1H), 7.15 (t, *J* = 8.6 Hz, 2H), 4.53 (s, 2H), 3.80 (s, 2H), 2.97 (d, *J* = 10.6 Hz, 2H), 2.65 (t, *J* = 7.0 Hz, 2H), 2.45 (d, *J* = 6.9 Hz, 2H), 1.92 (s, 2H), 1.68 (d, *J* = 10.2 Hz, 2H), 1.47 (m, 2H), 1.37 (m, 3H), 1.12 (t, *J* = 7.1 Hz, 3H). ^13^C NMR (CDCl_3_, 100 MHz): δ 162.70 (d, *J*_CF_ = 247.1 Hz), 156.15, 145.49, 138.08, 133.83, 133.80, 132.94, 131.71 (2C), 129.15 (2C), 128.50 (2C), 128.43, 127.14, 116.00 (d, *J*_CF_ = 21.6 Hz, 2C), 53.38, 52.60, 50.37 (2C), 46.99, 36.71, 34.36, 33.59 (2C), 32.10, 11.91. HRMS (ESI): *m*/*z* [M + H]^+^ calcd for C_28_H_35_FN_3_S, 464.2536; found, 464.25311.

##### WSA 308

**WSA 272** (58 mg, 0.13 mmol) and 2,4-difluorophenylboronic acid (30 mg, 0.19 mmol) were combined using general procedure **3** to produce **WSA 308** (25 mg, 40% yield) as an orange semisolid. ^1^H NMR (400 MHz, CDCl_3_): δ 8.51 (s, 1H), 7.70 (s, 1H), 7.41 (m, 3H), 7.31 (t, *J* = 7.2 Hz, 2H), 7.24 (t, *J* = 7.5 Hz, 1H), 6.95 (m, 2H), 4.53 (s, 2H), 3.79 (s, 2H), 2.92 (d, *J* = 10.6 Hz, 2H), 2.64 (t, *J* = 7.0 Hz, 2H), 2.37 (q, *J* = 6.8 Hz, 2H), 1.83 (t, *J* = 10.7 Hz, 2H), 1.66 (d, *J* = 11.6 Hz, 2H), 1.45 (q, *J* = 6.4 Hz, 2H), 1.29 (m, 3H), 1.08 (t, *J* = 7.2 Hz, 3H). ^13^C NMR (CDCl_3_, 100 MHz): δ 162.58 (dd, *J*_CF_ = 249.0, 12.0 Hz), 159.92 (dd, *J*_CF_ = 249.0, 11.0 Hz), 156.77, 146.92 (d, *J*_CF_ = 3.5 Hz), 137.99, 135.47 (d, *J*_CF_ = 2.9 Hz), 132.68, 131.03 (q, *J*_CF_ = 4.8 Hz), 129.18 (2C), 128.51 (2C), 127.16, 126.67 (d, *J*_CF_ = 1.3 Hz), 121.88 (q, *J*_CF_ = 6.0 Hz), 111.92 (d, *J* = 21 Hz), 104.63 (t, *J*_CF_ = 25.9 Hz), 53.42, 52.62, 50.23 (2C), 46.93, 36.75, 34.29, 33.64 (2C), 32.18, 12.00. HRMS (ESI): *m*/*z* [M + H]^+^ calcd for C_28_H_34_F_2_N_3_S, 482.2442; found, 482.24374.

### Biological Evaluation

#### General Sterilization Procedure

The following are general steps, unless otherwise noted. All steps were completed with aseptic techniques. All media and glassware were sterilized via autoclave at 121 °C for 60 min. All agitation occurred at 160 rpm in a temperature-controlled console shaker (Excella E25) at 37 °C. Full strength tryptic soy broth (TSB) was made by dissolving 30 g BD Bacto TSB powder in 1 L deionized water. All bacterial strains were purchased from ATCC (*A. baumannii* ATCC 17978 and ATCC 1605, MDR).

#### Antimicrobial Susceptibility Assay Procedure

Susceptibility testing was performed in biological triplicate, using the broth microdilution method as outlined by the Clinical and Laboratory Standards Institute. Briefly, minimum inhibitory concentrations (MIC) determinations were carried out in 96-well microtiter plates with 2-fold serial dilutions of the compounds from 0 μg/mL to 128 μg/mL (final assay concentrations, *n* = 3) in DMSO. To each well 1 μL of compound in DMSO, 89 μL of tryptic soy broth (TSB), and 10 μL of bacterial inoculum, grown from frozen stock in 10 mL of TSB for 12–16 h, were added. After incubation for 12–16 h at 37 °C, absorbance at 590 nm was read on a Biotek Synergy HTX Multimode plate reader. Data was processed by background subtracting the media absorbance and then normalizing the data to full bacterial growth with only vehicle. MIC is defined as the lowest concentration of antibiotic that achieves ≥80% growth inhibition, which corresponds to no visible growth.

### Adjuvant Assay Procedure with Colistin Sulfate

#### Checkerboard Assay

Synthesized compounds in DMSO (2–128 μg/mL, row G-A) were combined with colistin sulfate in ultra purified water (0.125–32 μg/mL, column 2–10) in a 96-well microtiter plate checkerboard assay against ATCC 17978 and BAA-1605 (MDR). Briefly, liquid cultures of bacteria were grown for 14–16 h by inoculating a single bacterial culture into 10 mL TSB. To each well 88 μL of TSB, 10 μL of bacteria culture, and 1 from each master plate was added (see layout below). Plates were covered and incubated at 37 °C for 12–18 h. Optical density (OD_590_) was measured on a Biotek Synergy HTX-Multimode plate reader, and data was processed by background subtracting the media absorbance (H7–H12) and then normalizing the data to full bacteria growth with only vehicle (H1–H6). MIC is defined as the lowest concentration of antibiotic or antibiotic/adjuvant combination that achieves ≥80% growth inhibition (no visual growth).

#### Preparation of Inverted Inner Membrane Vesicles

*A. baumannii* (ATCC 17978) cells were grown in LB medium at 37 °C with shaking at 150 rpm and harvested by centrifugation in the late exponential phase of growth. Cells were resuspended in TMG buffer (50 mM Tris-HCl, pH 7.5, 5 mM MgCl_2_, 10% v/v glycerol) with 1 mM phenylmethanesulfonyl fluoride, 1 mM dithiothreitol, and a small amount of DNase and lysed by two passes through an Avestin B15 homogenizer at 19,000 psi. Lysate was cleared by centrifugation at 9000*g* and inverted membrane vesicles were collected from the supernatant by centrifugation at 169,000*g*. To reduce background ATP synthesis activity in AB vesicles, it was important to wash the membranes by resuspending in TMG buffer and centrifuging again at 169,000*g*. Washed pellets were resuspended in TMG buffer, and aliquots were stored at −80 °C. Once thawed, aliquots were not refrozen. Protein concentration in membrane vesicles was determined using a modified Lowry assay.^32^

#### Determination of ATP Synthesis Activity

In vitro ATP synthesis activity of inverted inner membrane vesicles was measured as previously described.^17^ A reaction solution was prepared containing 5 mM tricine-KOH, pH 8.0, 50 mM KCl, 2.5 mM MgCl_2_, 0.1 mM adenosine diphosphate, 3.75 mM potassium phosphate, and 2.5 mM NADH and distributed into a 96-well plate. NADH-driven ATP synthesis was initiated by addition of inverted vesicles to 50 μg/mL and allowed to proceed for 10 min. The reaction was stopped by transferring an aliquot into 1% trichloroacetic acid. The stopped reaction was diluted 100-fold with deionized water and a sample was transferred to luciferase solution containing 25 mM tricine-NaOH, pH 7.8, 5 mM MgSO_4_, 0.1 mM EDTA, 0.1 mM NaN_3_, 150 μg/mL luciferin, and 7.5 μg/mL luciferase. Luminescence was measured in an opaque white 96-well plate using a BioTek H1 multimode plate reader. Each replicate set included a positive control containing DMSO with no compound and a negative control containing carbonyl cyanide 3-chlorophenylhydrazone (CCCP). Luminescence values were corrected for background by subtracting the CCCP control and normalized to the luminescence of the DMSO control. Normalized activity was fit using a variable-slope dose response curve. Nonlinear regression, including prediction of 95% confidence bands, and pairwise comparisons of fits using the F-test method were completed using GraphPad Prism10.

$$Relative\;activity=\frac{1}{1+{\left(\frac{c}{IC_{50}}\right)}^{Hillslope}}$$

#### Determination of ETC Activity

NADH-driven proton pumping activity in inverted membrane vesicles was measured as previously described.^17^ Briefly, vesicles were diluted to 0.5 mg/mL in HMK buffer (50 mM HEPES, 2 mM MgCl_2_, 300 mM KCl, pH 7.5) with 0.3 μg/mL 9-amino-6-chloro-2-methoxyacridine (ACMA) and distributed into wells of a black 96-well plate containing various concentrations of inhibitors dissolved in DMSO. ACMA fluorescence (λ_ex_ = 415 nm, λ_em_ = 485 nm) was monitored using a BioTek H1 multimode plate reader. Proton pumping was initiated by addition of NADH to 0.8 mM and terminated by addition of nigericin to 0.5 μg/mL. Activity was defined as the percent quenching of fluorescence, where fluorescence values are normalized to the maximum fluorescence following the addition of nigericin.

### Computational Docking

The cryo-EM structure of AB ATP Synthase (PDB ID: 7P2Y)^22^ was initially prepared in PyMOL (Schrödinger). The F_1_ (α, β, γ, δ and ε) and F_o_ b_2_ subunits were manually deleted, leaving a clean structure of the AB F_0_-ac_10_ complex. This complex was then submitted to the H++ 4.0 server (http://biophysics.cs.vt.edu/H++)^23,33^ to obtain a complete structure accounting for membrane-specific electrostatics (salinity = 0.15, internal dielectric = 4, external dielectric = 80, pH = 7). The resulting protein with accurate protonation states was then processed with a custom BioPython script to reassign chain identifiers and restore residue numbering. A docking gridbox, centered on *c*Asp60, *a*Trp261, and *a*Phe264, was defined using MGLTools 1.5.7 (ccsb.scripps.edu/mgltools/).

Ligands were converted from ChemDraw CDXMLs to MOLs via the ChemDraw API. These were subsequently processed with the AllChem Module for Merck Molecular Force Field (MMFF) energy minimization from RDKit (http://www.rdkit.org) and the pH command (pH = 7) in Open Babel (http://www.openbabel.org),^24^ and then converted to PDB format. These ligands were further processed in PDBQT format using the prepare_ligand4.py script from MGLTools with default parameters.

Computational docking was performed in batch using GNINA 1.3.^34^ Each GNINA output conformation was saved individually with the receptor as a PDB using the PyMOL API. For interaction level analysis, these PDBs were passed through the Protein Ligand Interaction Profiler (PLIP)^35^ with default conditions. Docking results were converted into 2D images using the ProLIF library. Final results were analyzed visually in PyMOL, and quantitatively using NumPy, pandas, and Matplotlib in Python. Pose selection prioritized those with favorable binding energy and specific interactions: salt-bridge formation with *c*Asp60, π-Stacking with *a*Phe264, *c*Phe53, and/or *a*Trp261. When these criteria were not met, poses with the greatest number of hydrogen bonds were selected.

### Cytotoxicity Assay

Human Embryonic Kidney cells (HEK 293) were cultured in Dulbecco’s modified Eagle medium (DMEM) supplemented with 10% fetal bovine serum (FBS) and maintained in 10 cm tissue culture dishes. For viability assays, cells were rinsed with phosphate-buffered saline (PBS), trypsinized, and seeded at a density of approximately 2 × 10^4^ cells per well in 96-well flat bottom plates, with a final volume of 100 μL per well. Plates were incubated overnight at 37 °C in a humidified atmosphere containing 5% CO_2_. After the incubation period, the culture medium was removed, and 1 μL of various concentrations of inhibitors dissolved in DMSO (ranging from 2 to 128 μg/mL) were added to 99 μL of fresh medium per well. Plates were incubated for 24 h. Cell viability was assessed using the XTT assay (Biotum). After treatment, 50 μL of XTT solution was added to each well. Absorbance was measured at 490 nm with a reference wavelength of 650 nm using a BioTek H1 multimode plate reader. Initial plate readings were taken at 5 and 24 h intervals, with the 24 h incubation yielding the most consistent results.

## Supplementary Material



## Notes

Summary of ATP synthesis and ETC inhibition (Figures S1, S2 and Table S1), docking of WSA compounds to AB ATP synthase (Table S2), checkerboard assay data with colistin (Figure S3), ^1^H and ^13^C NMR spectra for compounds (PDF)

## Abbreviations

AB: *Acinetobacter baumannii*
ACMA: 9-amino-6-chloro-2-methoxyacridine
ATP: adenosine triphosphate
BDQ: bedaquiline
Bn: benzyl
DCM: dichloromethane
CCCP: carbonyl cyanide 3-chlorophenylhydrazone
DMSO: dimethyl sulfoxide
EDTA: ethylenediaminetetraacetic acid
ETC: electron transport chain
HEK: human embryonic kidney
HEPES: *N*-2-hydroxyethylpiperazine-*N*′-2-ethanesulfonic acid
IC_50_: inhibitory concentration of 50%
MDR: multidrug resistant
MIC: minimum inhibitory concentration
MT: *Mycobacterium tuberculosis*
NADH: nicotinamide adenine dinucleotide (reduced)
OM: outer membrane
SAR: structure activity relationship
TSB: tryptic soy broth
FICI: fractional inhibitory concentration index
