# Supplementary material for: Synthesis and Evaluation of Pyridine-Based Antibacterial Agents that Inhibit ATP Synthase in Acinetobacter baumannii
Source: ACS Omega. 2025 Sep 26;10(39):45823–39. doi: 10.1021/acsomega.5c06380 (PMC12509006; doi:10.1021/acsomega.5c06380)

Synthesis and evaluation of pyridine-based antibacterial agents that inhibit ATP synthase in *Acinetobacter baumannii*

Angelina L. Dennison,[1,‡] Armaan Singh, [1,‡] Toni A. Marchlewski,[1] Sierra N Ghee,[1] Ava G. Hammock,[1] Kevin C. Gencel,[1] Sabrina Liu,[1] Shaylla Wilson,[1] Alexander P. L. Williams,[1] Katie T. Ward,[1] Thomas E. Meigs,[2] P. Ryan Steed, [1*] Amanda L. Wolfe[1*]

[1]Department of Chemistry and Biochemistry, University of North Carolina Asheville, One University Heights, Asheville, North Carolina, 28804, United States
[2]Department of Biology, University of North Carolina Asheville, One University Heights, Asheville, North Carolina, 28804, United States

Supplementary Information

Table of Contents

Figure S1. AB ATP synthase inhibition data and fits S2

Table S1. AB ATP synthase inhibition IC_{50} values and Hill coefficients S5

Figure S2. Electron transport chain inhibition S7

Figure S3. Colistin and inhibitor checkerboard assays S8

Table S2. Computational docking of WSA compounds with AB ATP synthase S9

Figures S4-S9. ^{1}H and ^{13}CNMR Spectra S26

Figure S1. ATP synthesis inhibition by all pyridine compounds in order of WSA compound number. ATP synthesis activities in inverted inner membrane vesicles prepared from AB cells were measured in the presence of 0.0625-64 μg/mL, corrected for background ATP, and normalized to activity with DMSO alone (see methods). For each plot, dots are normalized activities at each concentration (n≥3) on a log_2 scale, solid lines are fitted dose response curves, and dashed lines show the 95% confidence range of the fit. IC_{50} and Hill coefficient values for the fits are reported in Table S1.

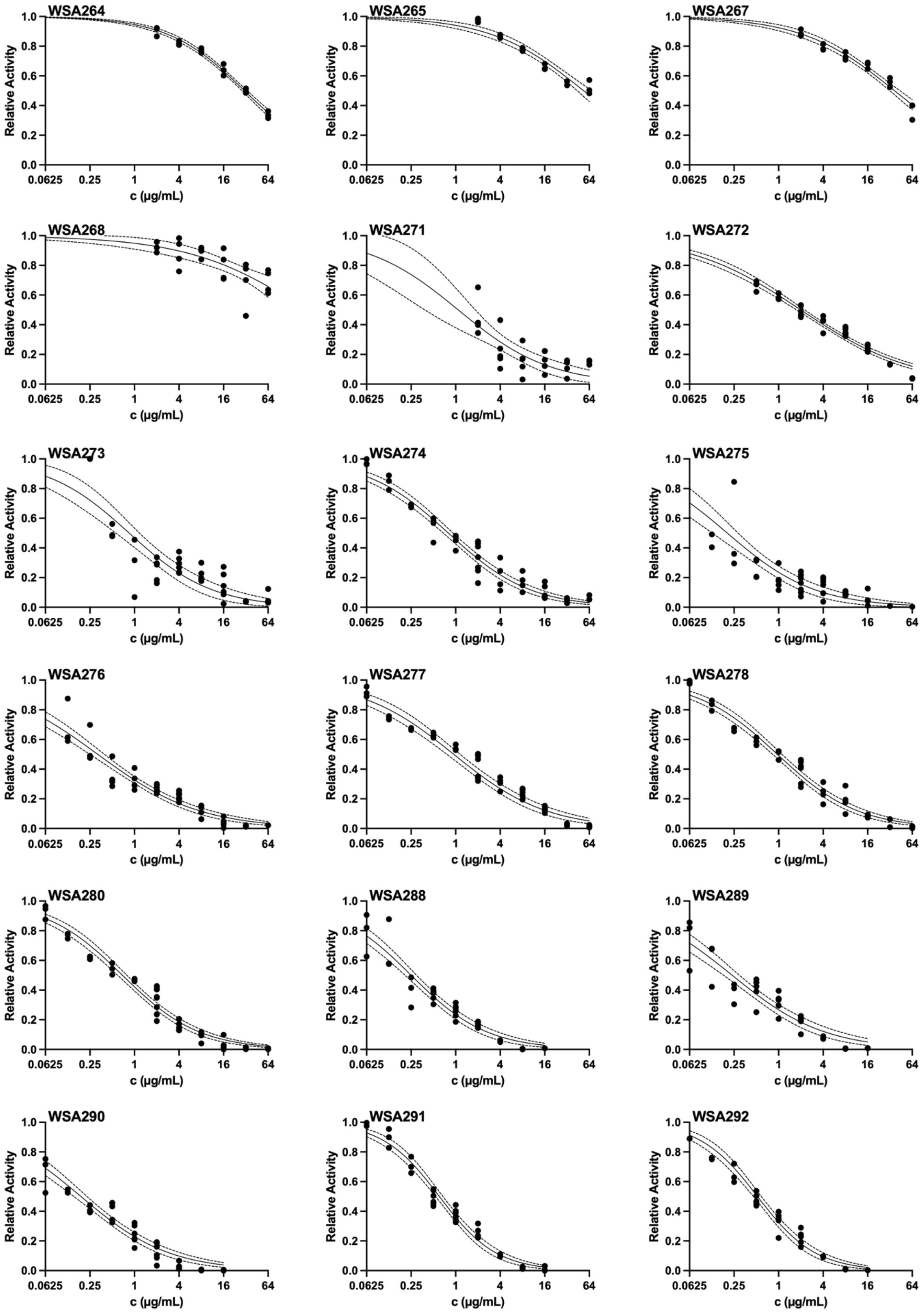
WSA264
WSA265
WSA267
WSA268
WSA271
WSA272
WSA273
WSA274
WSA275
WSA276
WSA277
WSA278
WSA280
WSA288
WSA289
WSA290
WSA291
WSA292
Relative Activity
1.0
0.8
0.6
0.4
0.2
0.0
0.0625
0.25
1
4
16
64
c (µg/mL)

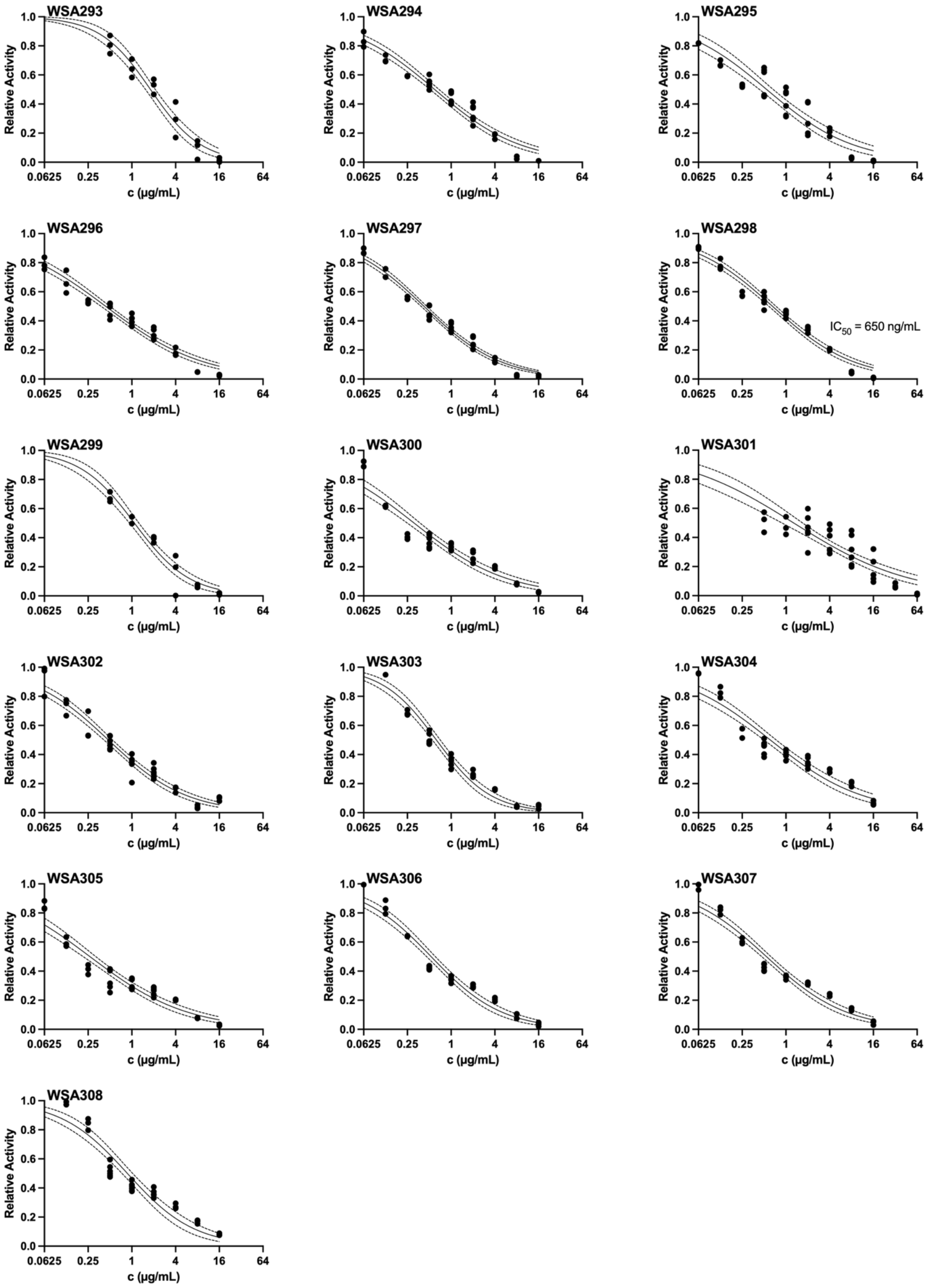
WSA293
WSA294
WSA295
WSA296
WSA297
WSA298
IC_{50} = 650 ng/mL
WSA299
WSA300
WSA301
WSA302
WSA303
WSA304
WSA305
WSA306
WSA307
WSA308
Relative Activity
c (µg/mL)
0.0625
0.25
1
4
16
64
0.0
0.2
0.4
0.6
0.8
1.0

Table S1. AB ATP synthase inhibition IC_{50} values and Hill coefficients

Compound	X	AB ATP Synthase Inhibition	
		IC_{50} (μg/mL)	Hill coefficient
**Y = Methyl piperizine**			
WSA 268	H	>64	-0.5
WSA 271	Br	1.0	-0.7
WSA 277	Ph	0.94	-0.7
WSA 280	4-methylphenyl	0.69	-0.8
WSA 278	4-methoxyphenyl	0.96	-0.8
WSA 276	4-(N,N-dimethylamine)phenyl	0.32	-0.6
WSA 273	4-fluorophenyl	0.85	-0.8
WSA 274	2,4-difluorophenyl	0.80	-0.8
WSA 275	3-fluoro-4-methoxyphenyl	0.24	-0.7
**Y = Ethyl piperidine**			
WSA 264	H	31	-0.8
WSA 272	Br	2.0	-0.6
WSA 304	Ph	0.61	-0.7
WSA 300	4-methylphenyl	0.31	-0.7
WSA 305	4-methoxyphenyl	0.27	-0.6
WSA 307	4-fluorophenyl	0.54	-0.8
WSA 308	2,4-difluorophenyl	0.87	-0.9
WSA 306	3-fluoro-4-methoxyphenyl	0.53	-0.9
**Y = Cyclopentyl piperidine**			
WSA 265	H	54	-0.7
WSA 301	Br	1.3	-0.5
WSA 302	Ph	0.48	-0.8
WSA 289	4-methylphenyl	0.24	-0.7
WSA 288	4-methoxyphenyl	0.25	-0.9
WSA 303	4-(N,N-dimethylamine)phenyl	0.63	-1.2

WSA 291	4-fluorophenyl	0.60	-1.1
WSA 292	2,4-difluorophenyl	0.52	-1.1
WSA 290	3-fluoro-4-methoxyphenyl	0.19	-0.7
**Y = Benzyl pyrolidine**			
WSA 267	H	37	-0.7
WSA 293	Br	1.8	-1.2
WSA 294	Ph	0.59	-0.7
WSA 295	4-methylphenyl	0.54	-0.7
WSA 296	4-methoxyphenyl	0.44	-0.6
WSA 298	4-fluorophenyl	0.65	-0.8
WSA 299	2,4-difluorophenyl	1.1	-1.1
WSA 297	3-fluoro-4-methoxyphenyl	0.42	-0.8

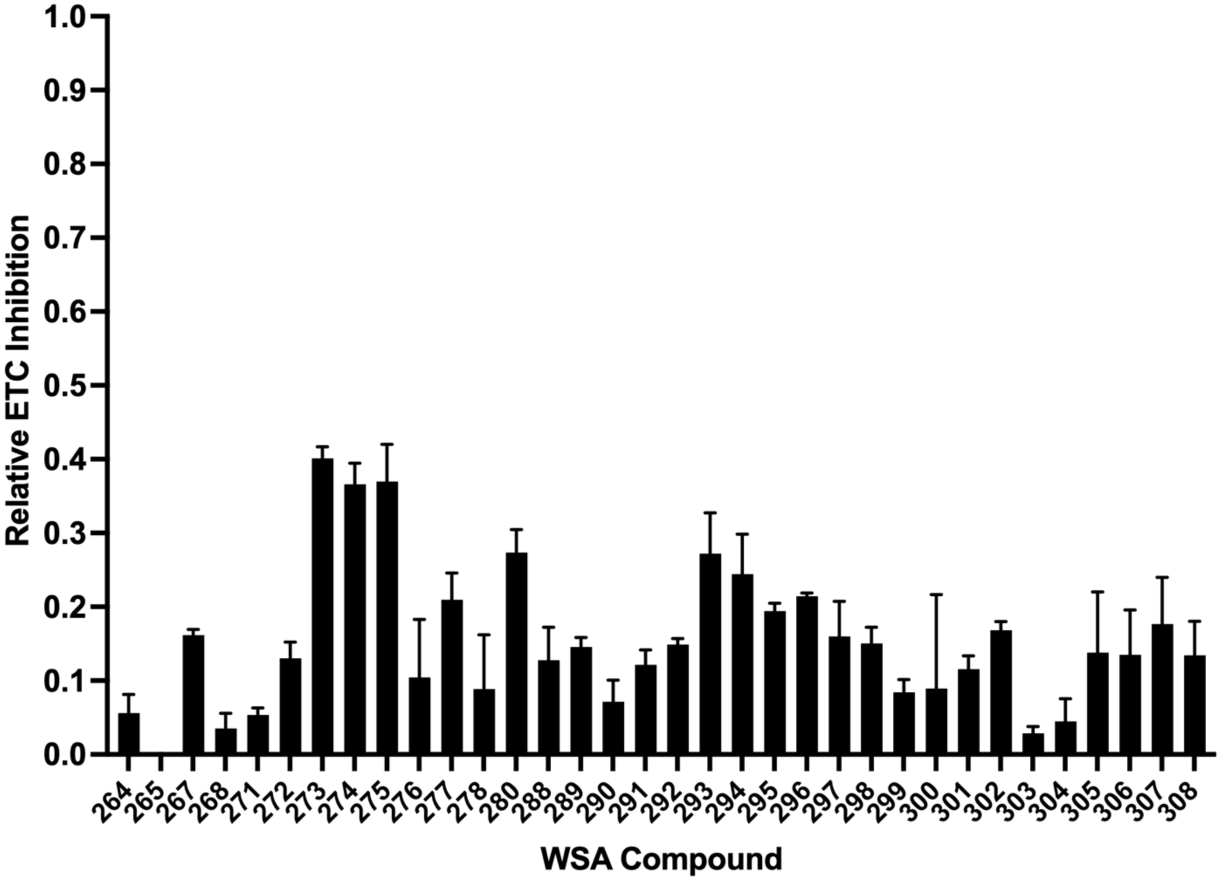

Figure S2. Inhibition of the AB electron transport chain (ETC) by compounds at 2 μg/mL. ETC H^+-pumping activity in inverted inner membrane vesicles prepared from AB cells was determined by the quenching of ACMA dye upon initiation with NADH. Addition of nigericin returned fluorescence to baseline, and relative fluorescence quenching is defined as the minimum fluorescence over the maximum fluorescence after addition of nigericin. Relative ETC inhibition (bars) is the fraction of fluorescence quenching decreased by the presence of 2 μg/mL compound.

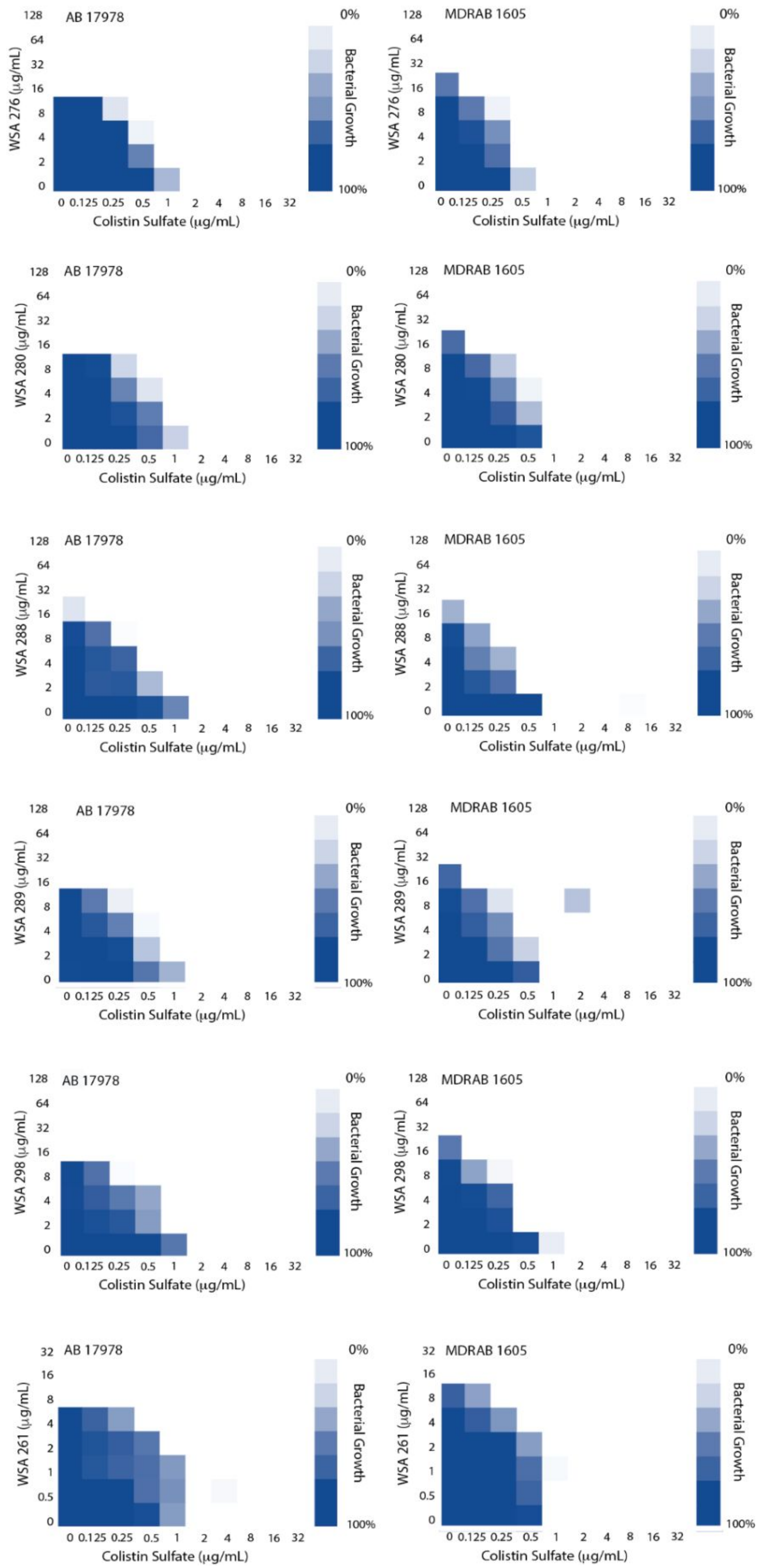
AB 17978
WSA 276 (μg/mL)
MDRAB 1605
WSA 276 (μg/mL)
AB 17978
WSA 280 (μg/mL)
MDRAB 1605
WSA 280 (μg/mL)
AB 17978
WSA 288 (μg/mL)
MDRAB 1605
WSA 288 (μg/mL)
AB 17978
WSA 289 (μg/mL)
MDRAB 1605
WSA 289 (μg/mL)
AB 17978
WSA 298 (μg/mL)
MDRAB 1605
WSA 298 (μg/mL)
AB 17978
WSA 261 (μg/mL)
MDRAB 1605
WSA 261 (μg/mL)
128 64 32 16 8 4 2 0
32 16 8 4 2 1 0.5 0
0 0.125 0.25 0.5 1 2 4 8 16 32
Colistin Sulfate (μg/mL)
0%
Bacterial Growth
100%

Figure S3. Colistin (0.125 μg/mL to 32 μg/mL) and inhibitor (2 μg/mL to 128 μg/mL) checkerboard synergy assay.

Table S2: Computational Docking of WSA compounds with AB ATP synthase

Compound	2d ProLIF Interaction Map	Binding Energy (kcal/mol)
**WSA 264**	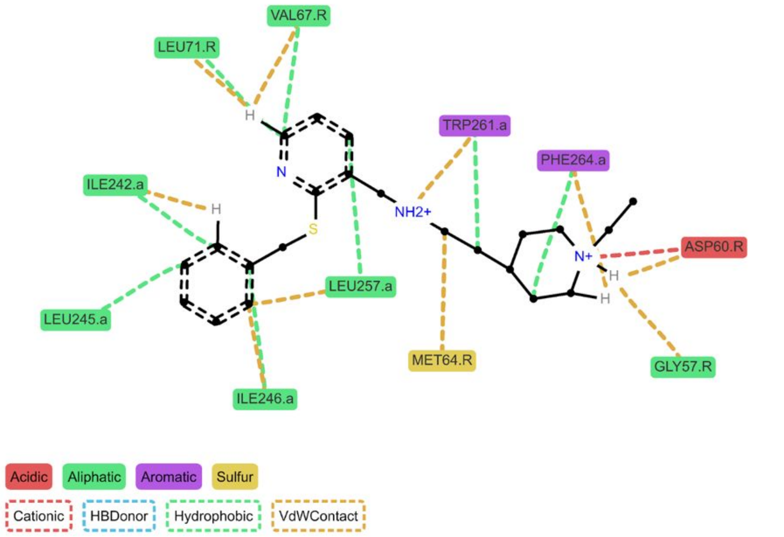	-7.41
**WSA 265**	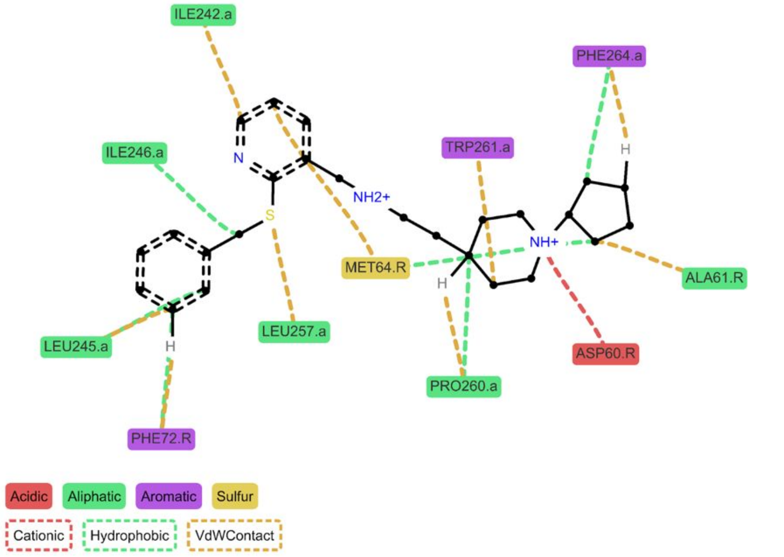	-7.74

**WSA 267**	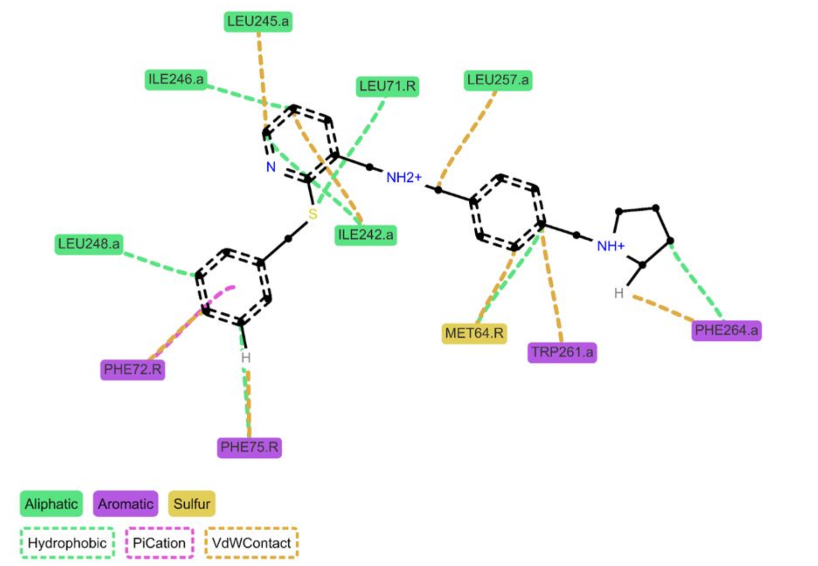	-8.44
**WSA 268**	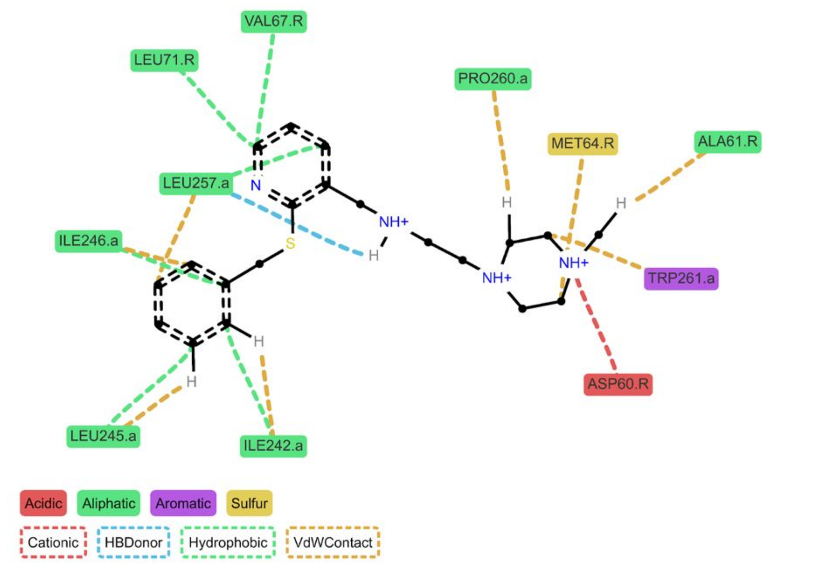	-7.14

**WSA 271**	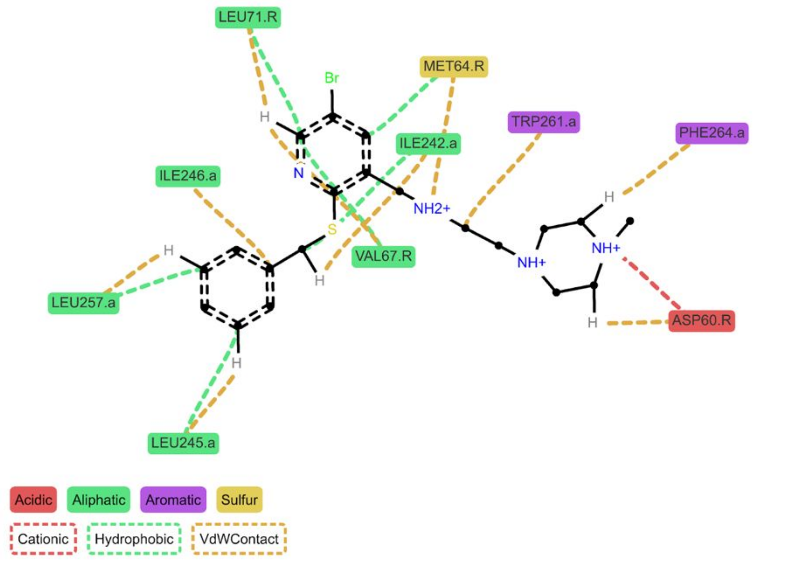	-7.29
**WSA 272**	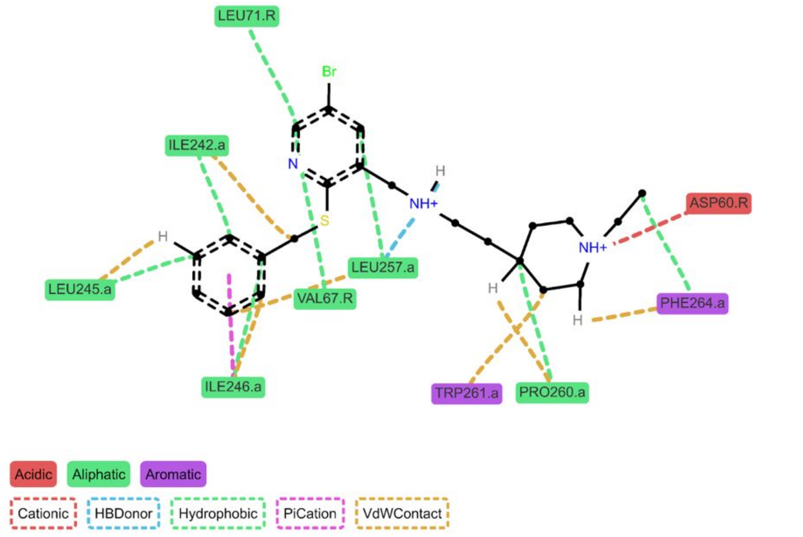	-7.39

**WSA 273**	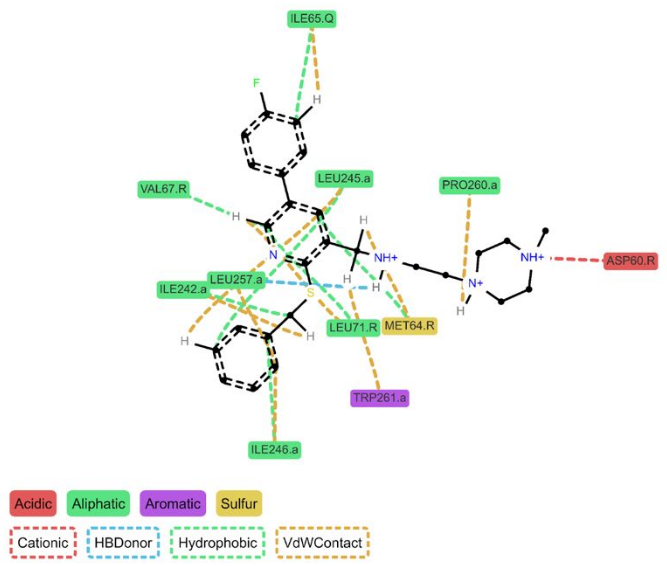	-7.97
**WSA 274**	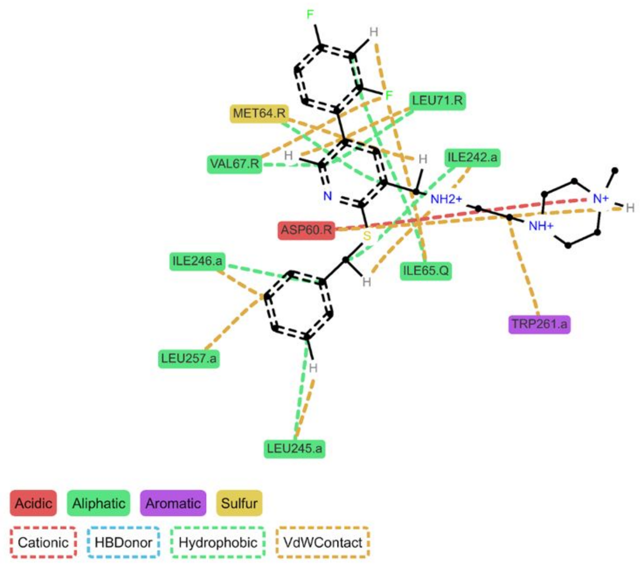	-8.29

<table>
<tr><td>WSA 275</td><td>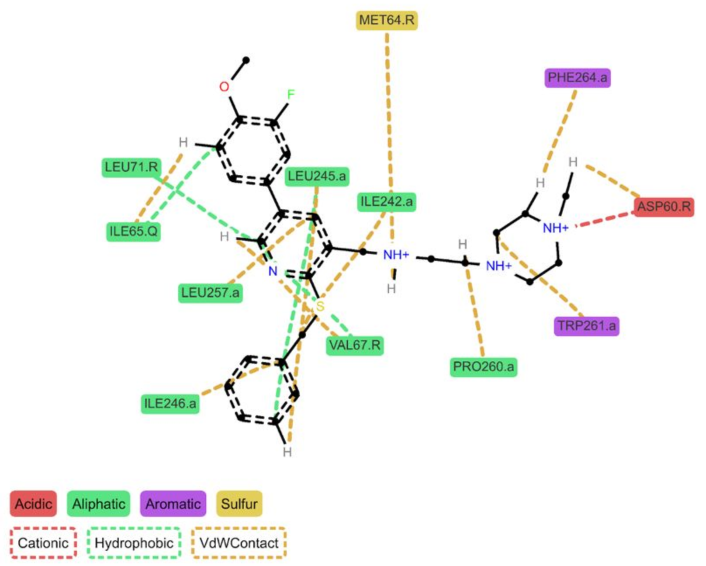
</td><td>-7.97</td></tr>
<tr><td>WSA 276</td><td>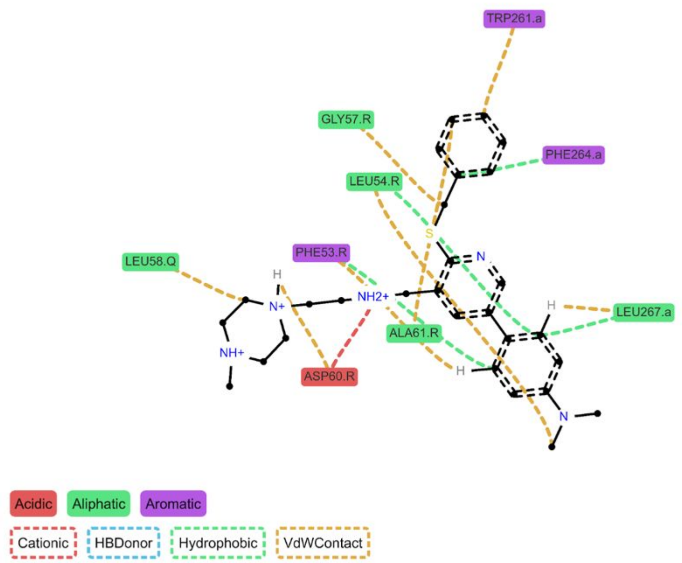
</td><td>-7.64</td></tr>
</table>

<table>
<tr><td>WSA 277</td><td>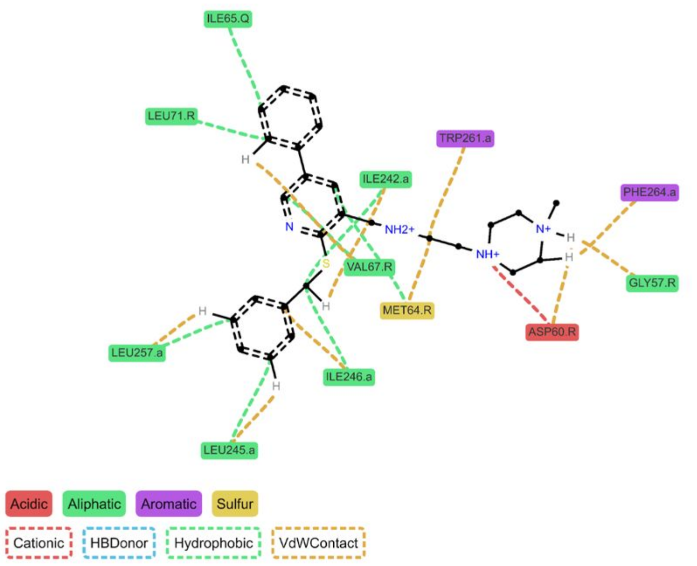
</td><td>-7.98</td></tr>
<tr><td>WSA 278</td><td>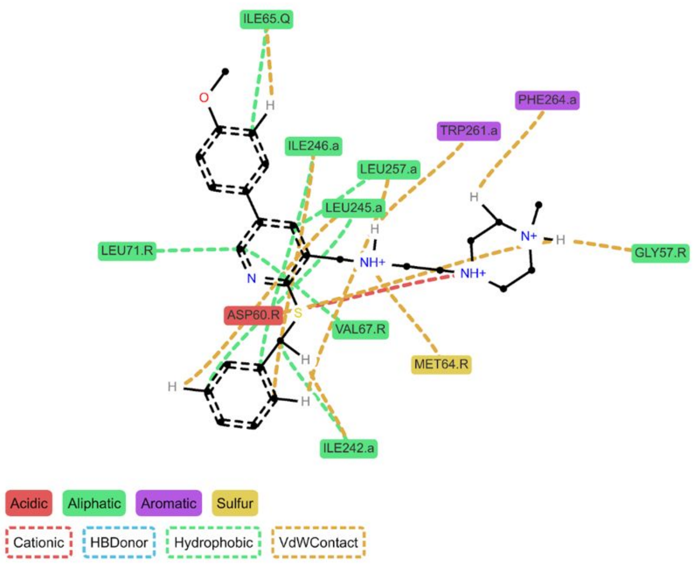
</td><td>-8</td></tr>
</table>

**WSA 280**	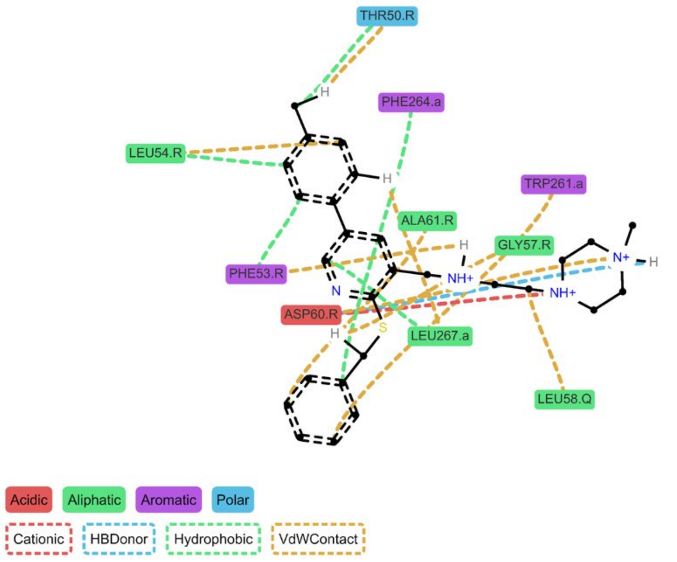	-7.88
**WSA 288**	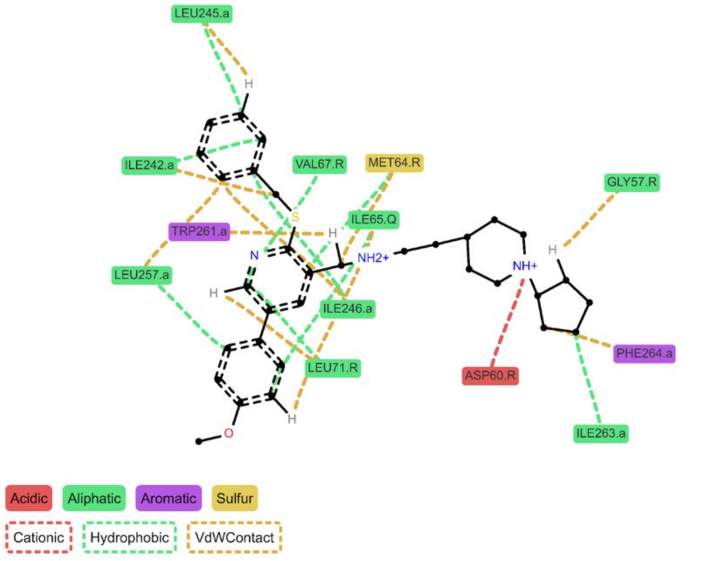	-8.54

<table>
<tr><td>WSA 289</td><td>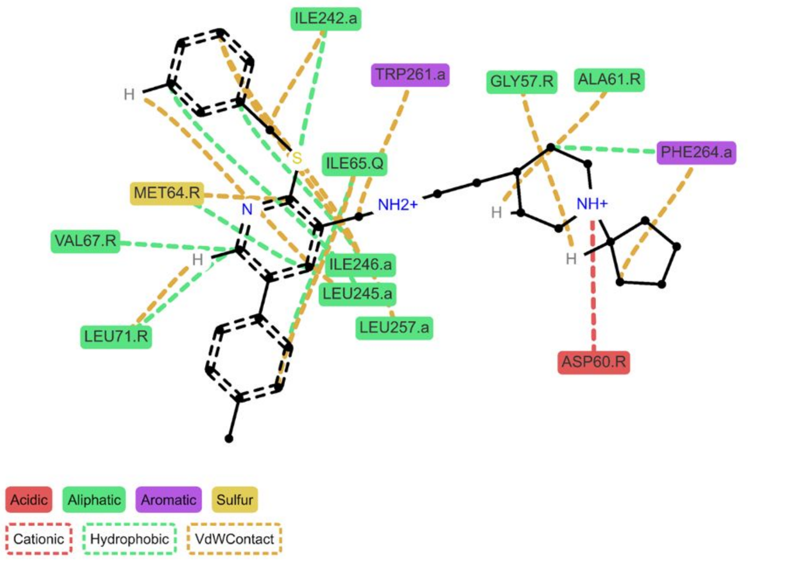
</td><td>-9.1</td></tr>
<tr><td>WSA 290</td><td>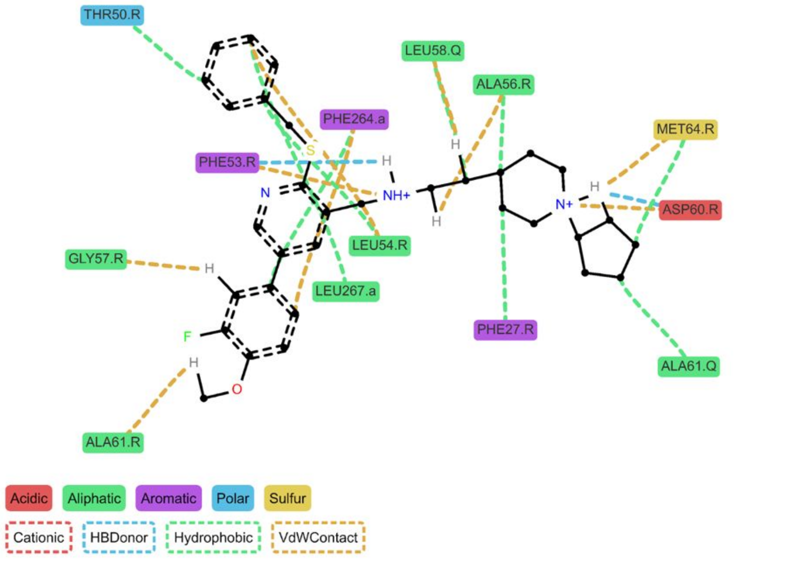
</td><td>-8.48</td></tr>
</table>

**WSA 291**	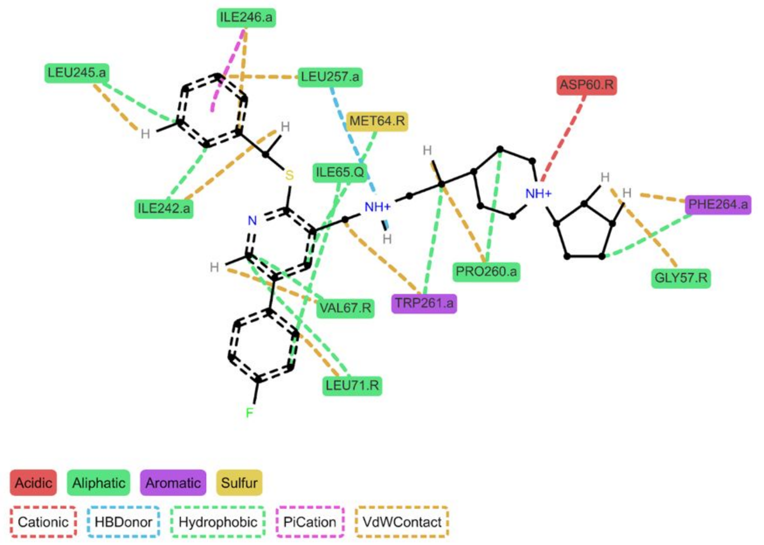	-8.6
**WSA 292**	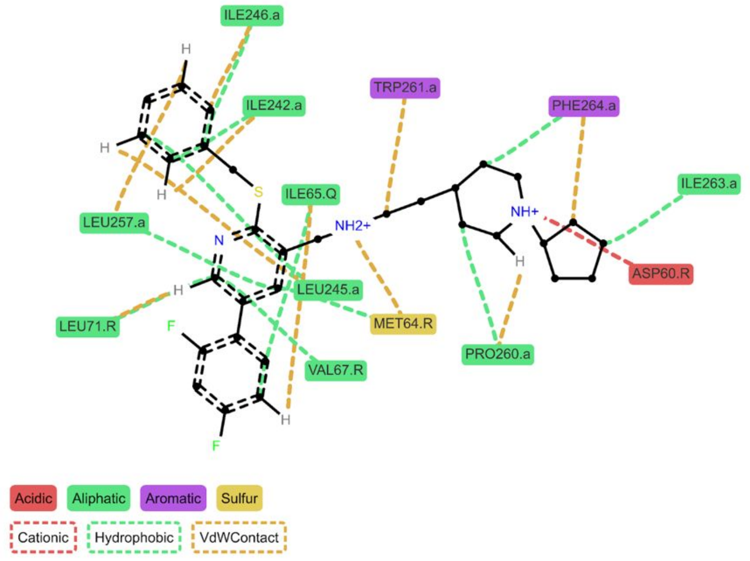	-8.98

**WSA 293**	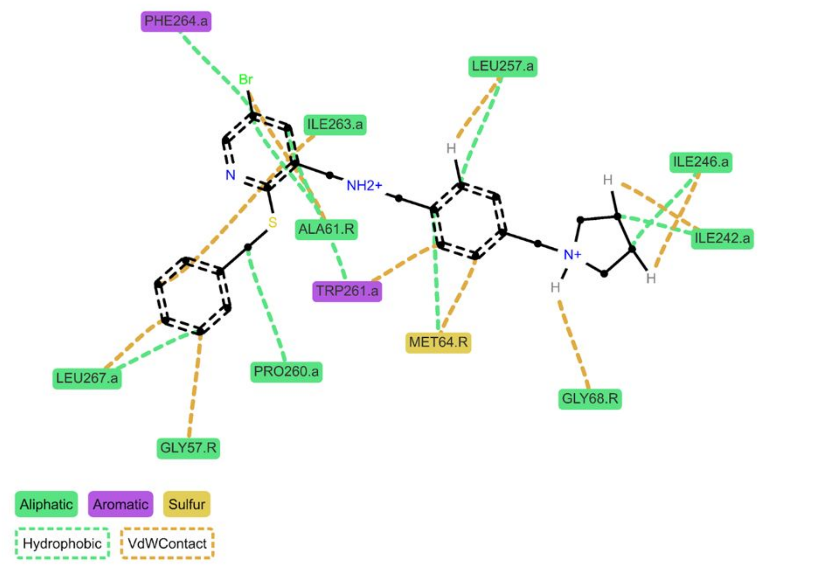	-7.81
**WSA 294**	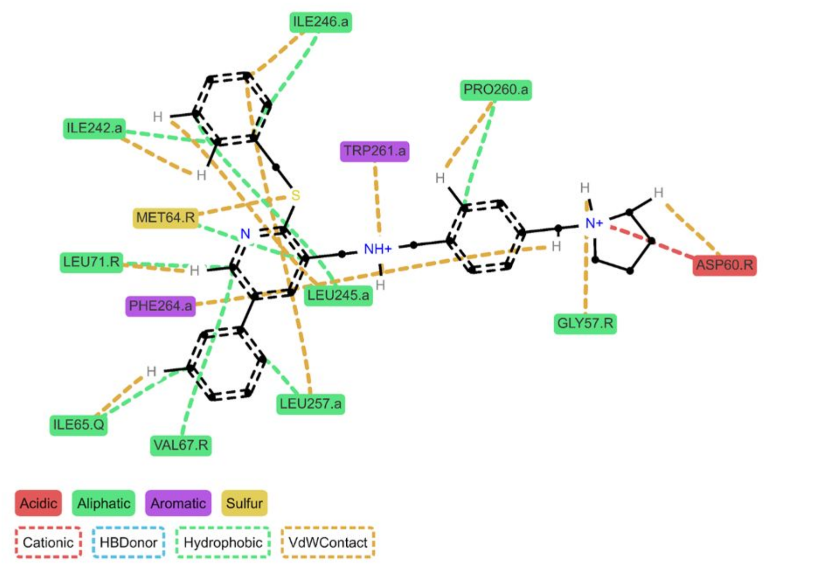	-8.82

**WSA 295**	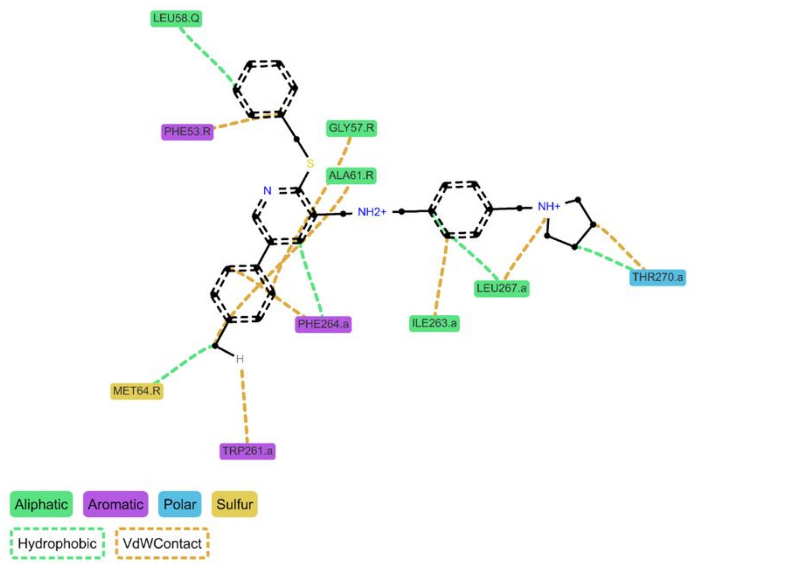	-8.7
**WSA 296**	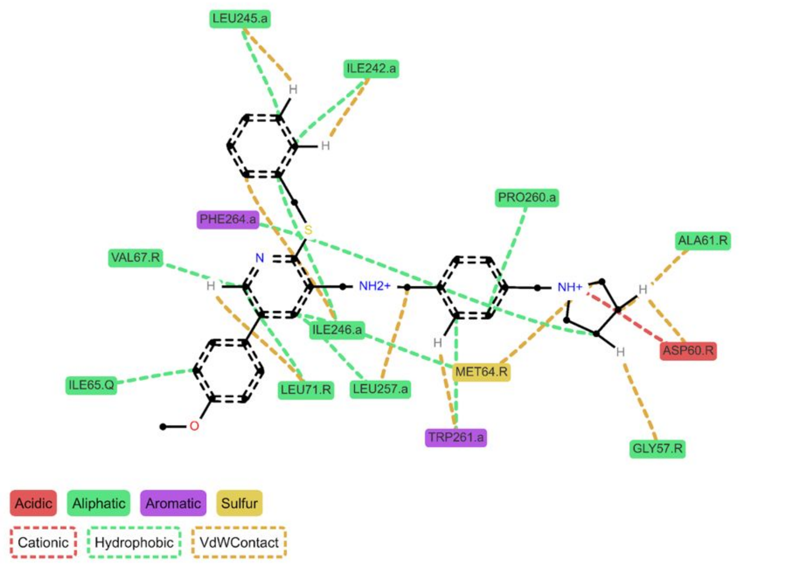	-8.7

**WSA 297**	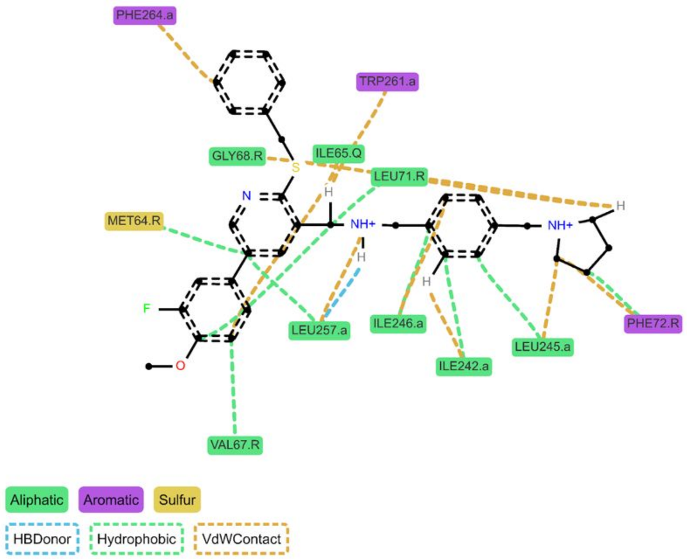	-8.42
**WSA 298**	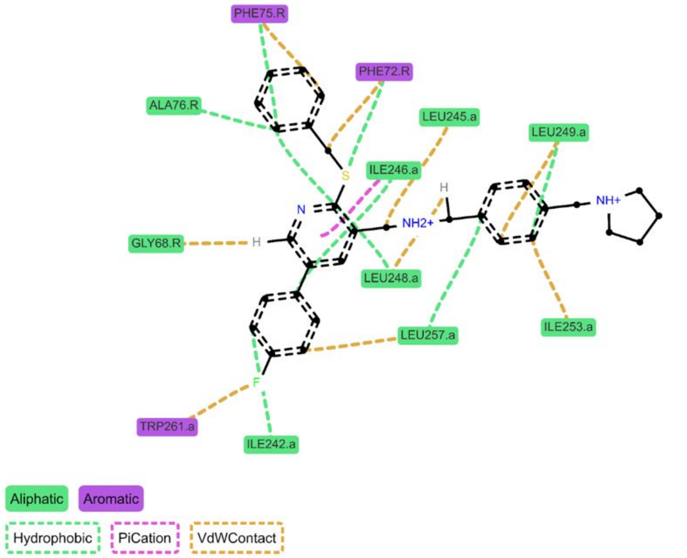	-8.58

<table>
<tr>
<td>WSA 299</td>
<td>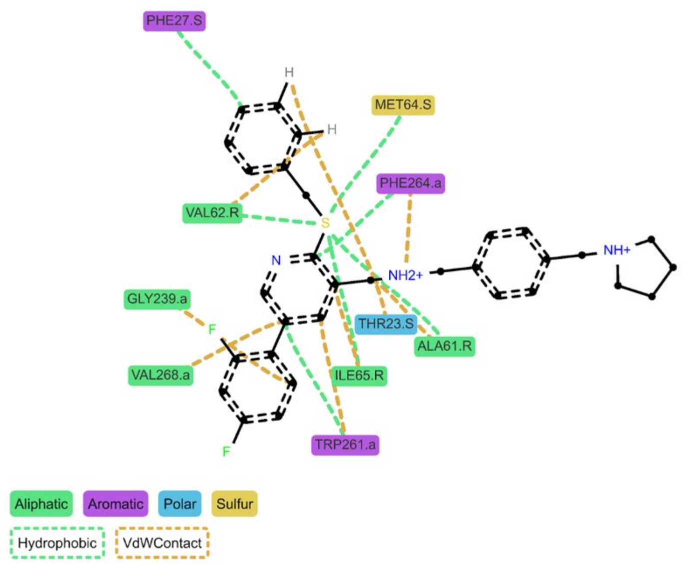
</td>
<td>-8.68</td>
</tr>
<tr>
<td>WSA 300</td>
<td>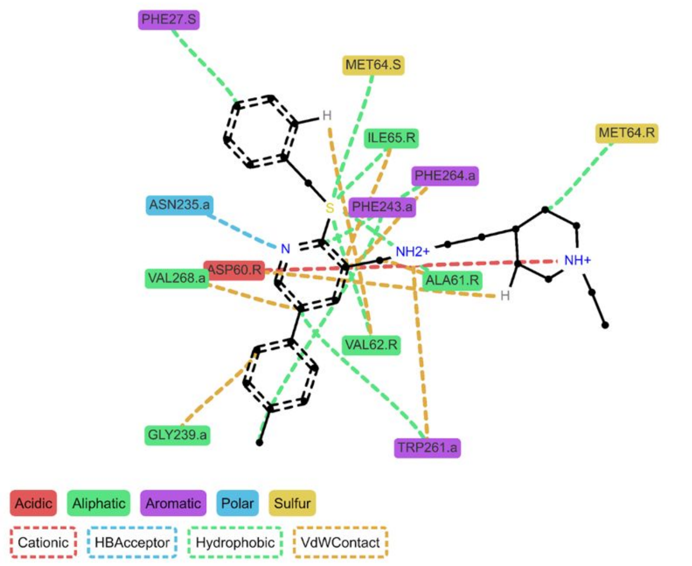
</td>
<td>-8.8</td>
</tr>
</table>

**WSA 301**	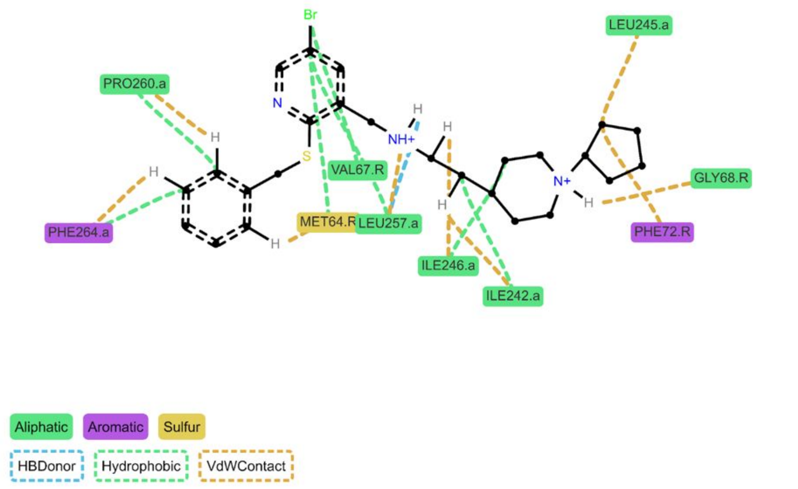	-8.29
**WSA 302**	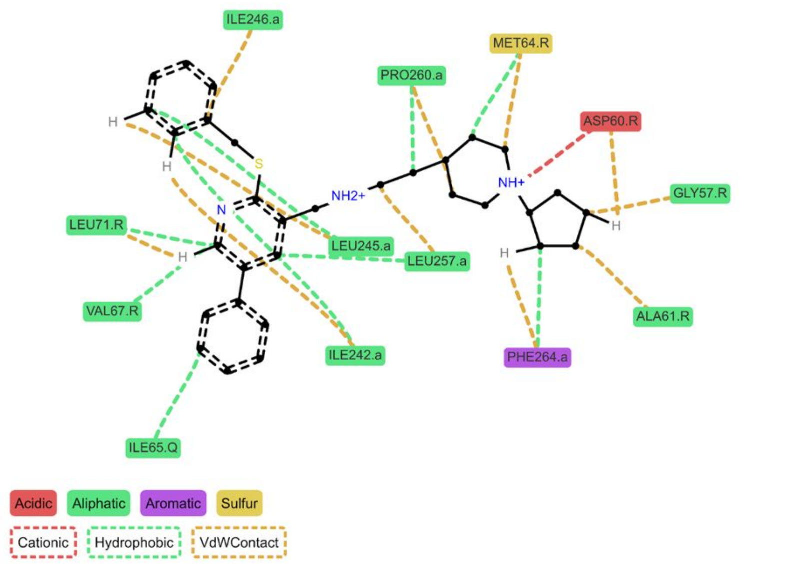	-9.16

<table>
<tr><td>WSA 303</td><td>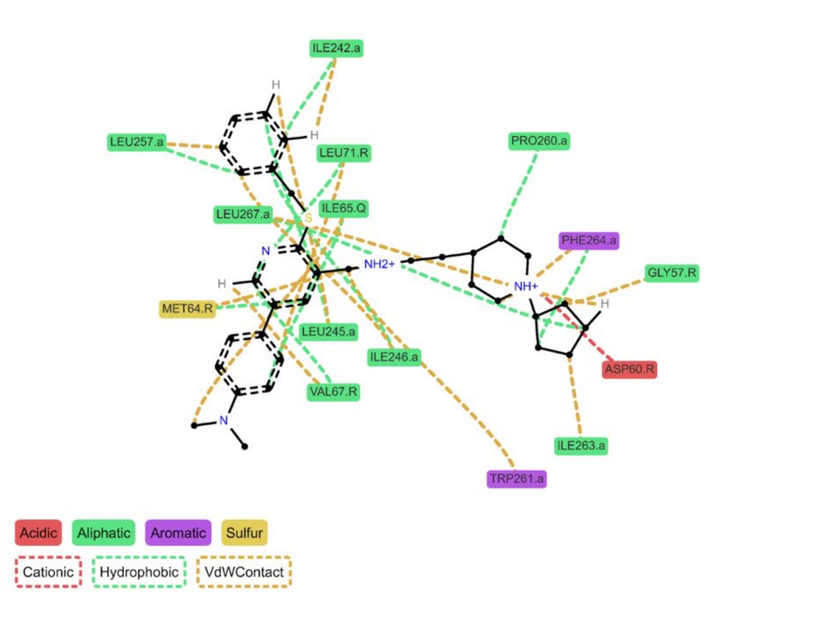
</td><td>-8.45</td></tr>
<tr><td>WSA 304</td><td>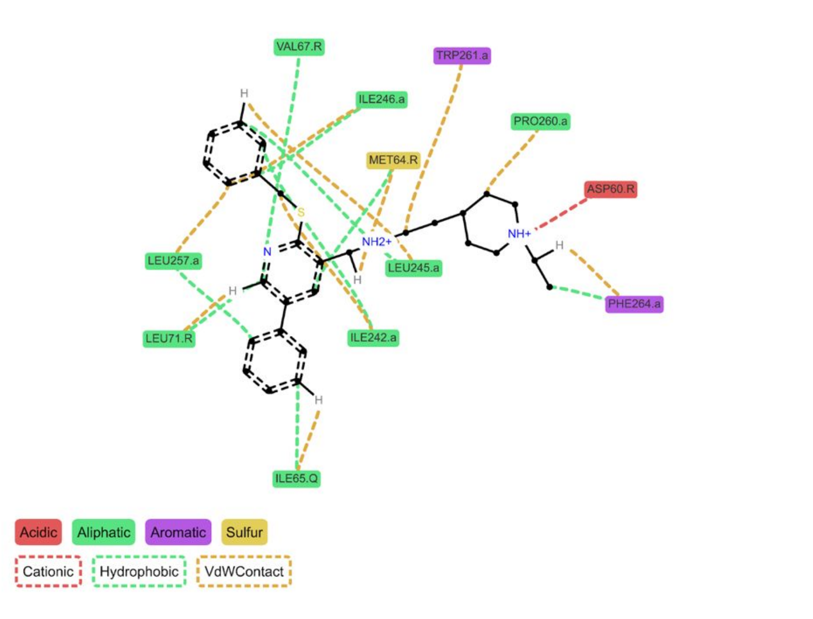
</td><td>-8.39</td></tr>
</table>

<table>
<tr>
<td>WSA 305</td>
<td>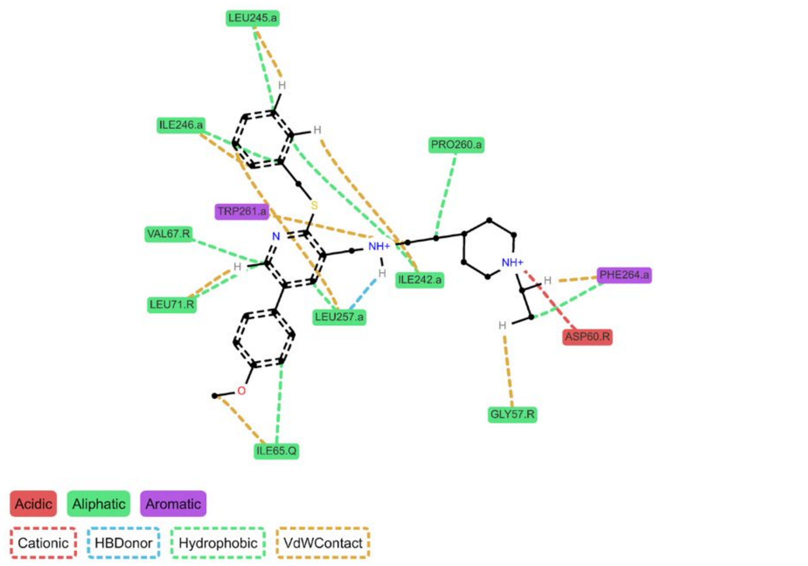
</td>
<td>-8.21</td>
</tr>
<tr>
<td>WSA 306</td>
<td>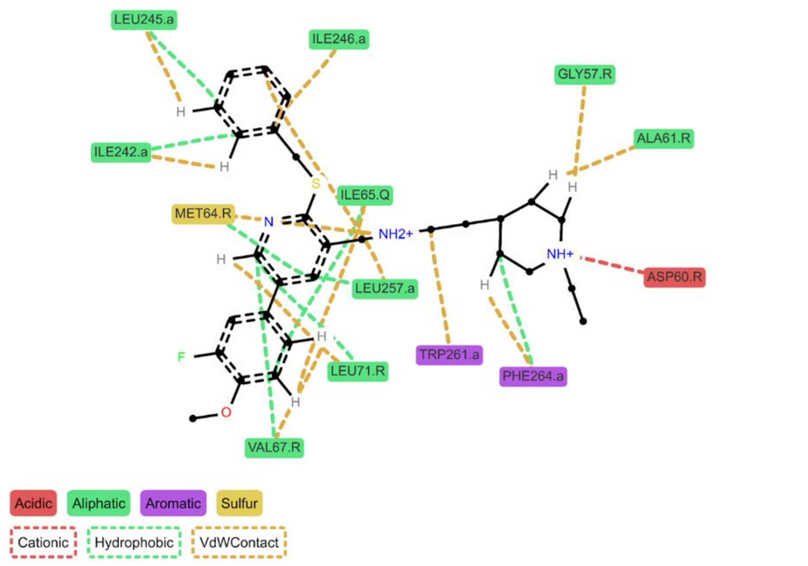
</td>
<td>-8.24</td>
</tr>
</table>

**WSA 307**	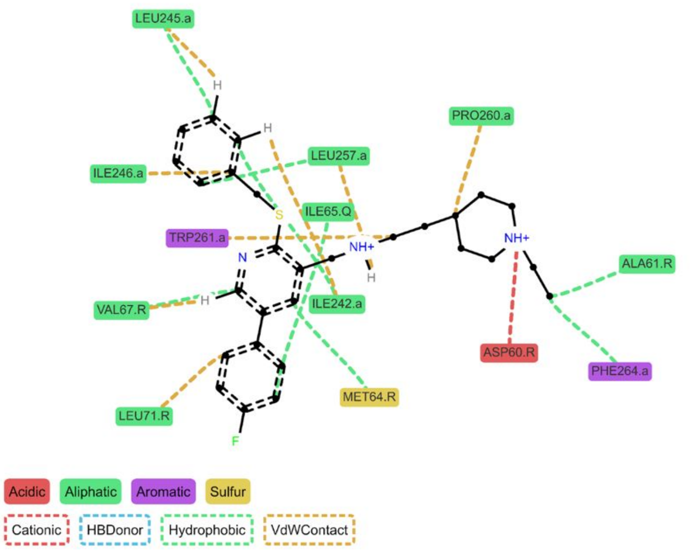	-8.38
**WSA 308**	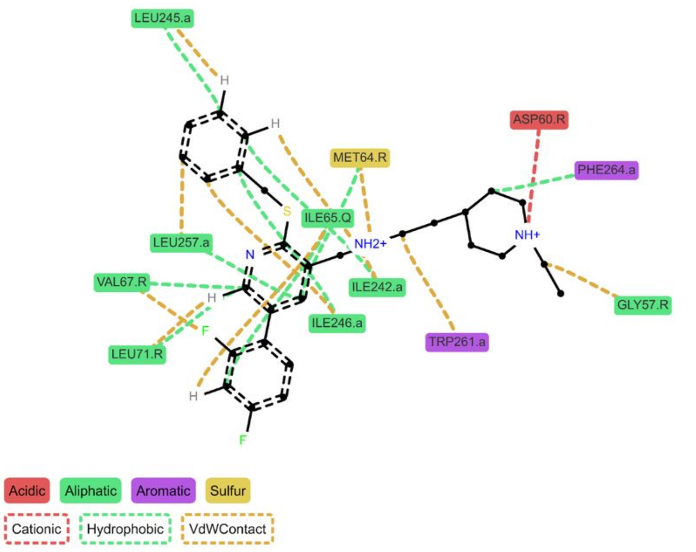	-8.46

Figure S4. Compound 3 ^{1}H and ^{13}C NMR spectra.

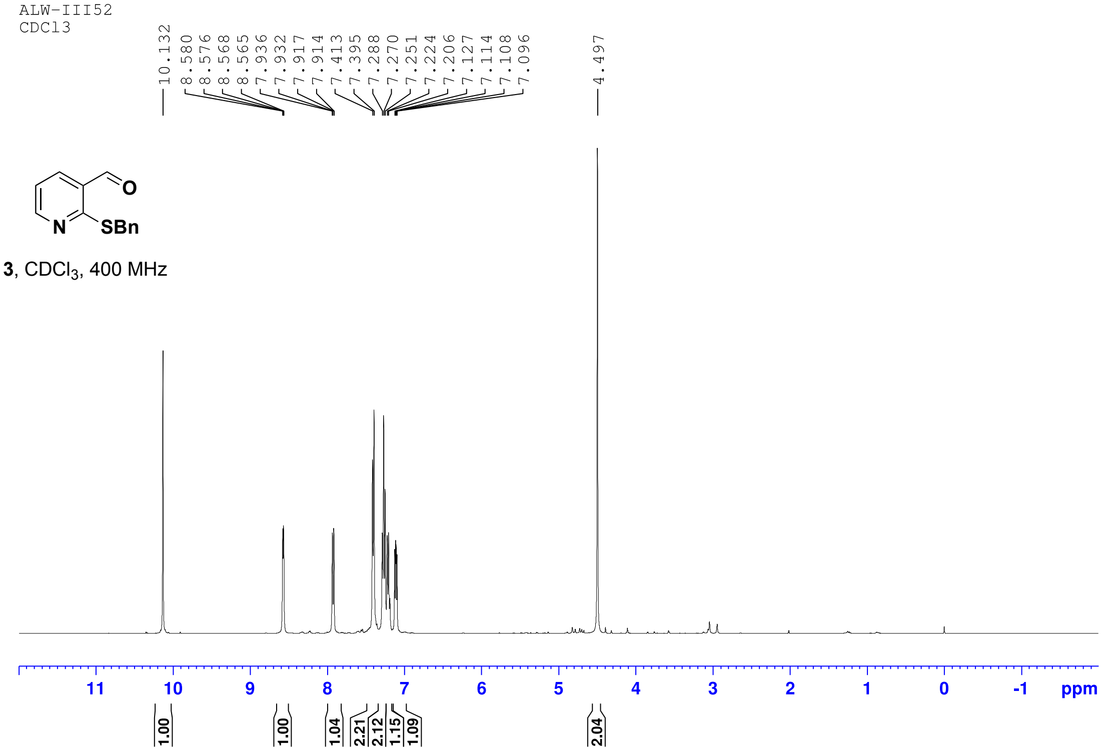

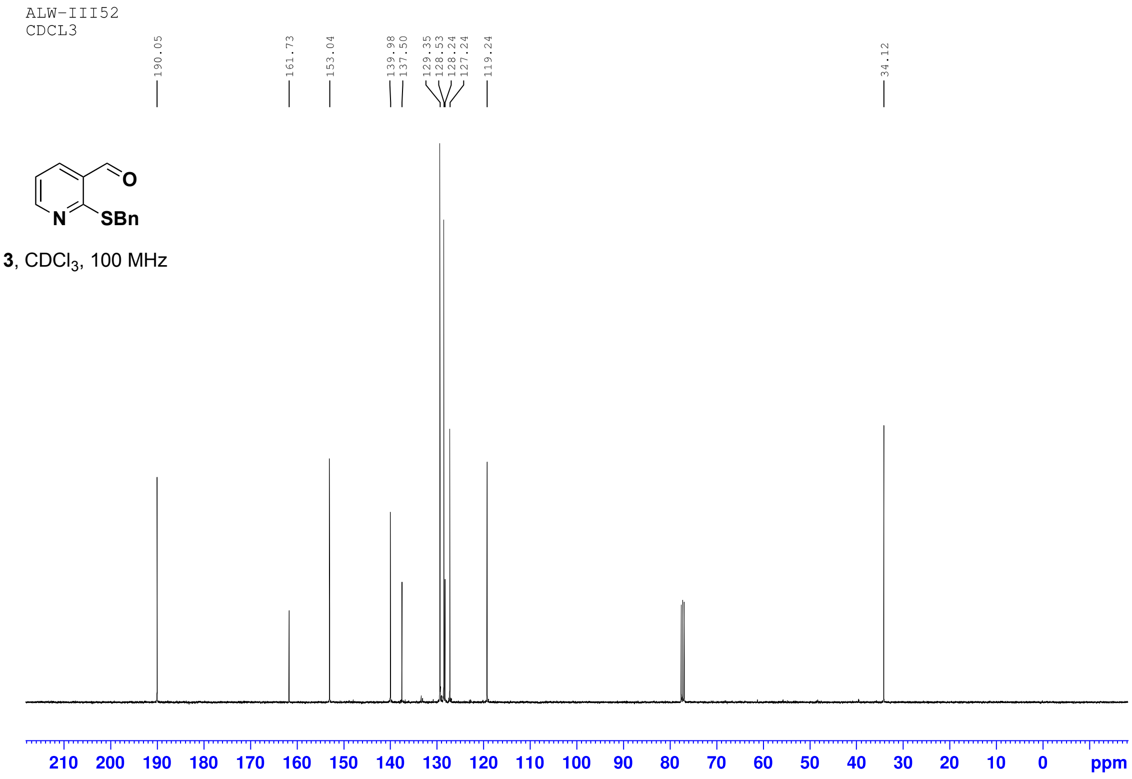

Figure S5. Compound 4 ^{1}H and ^{13}C NMR spectra.

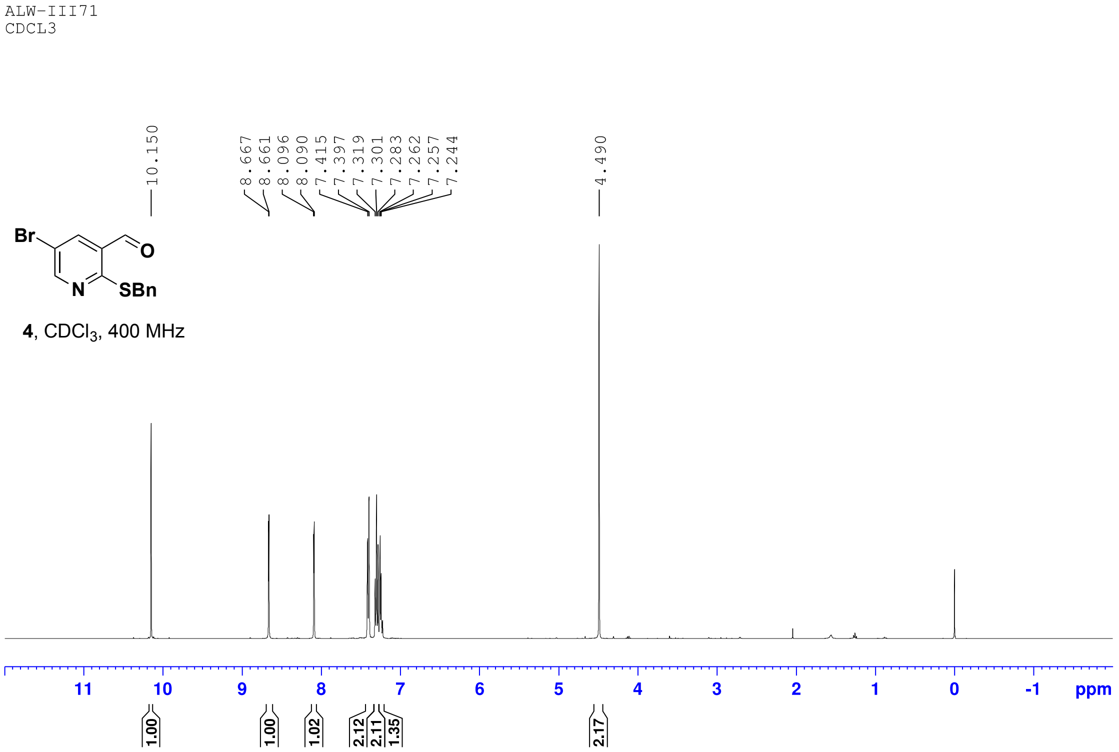

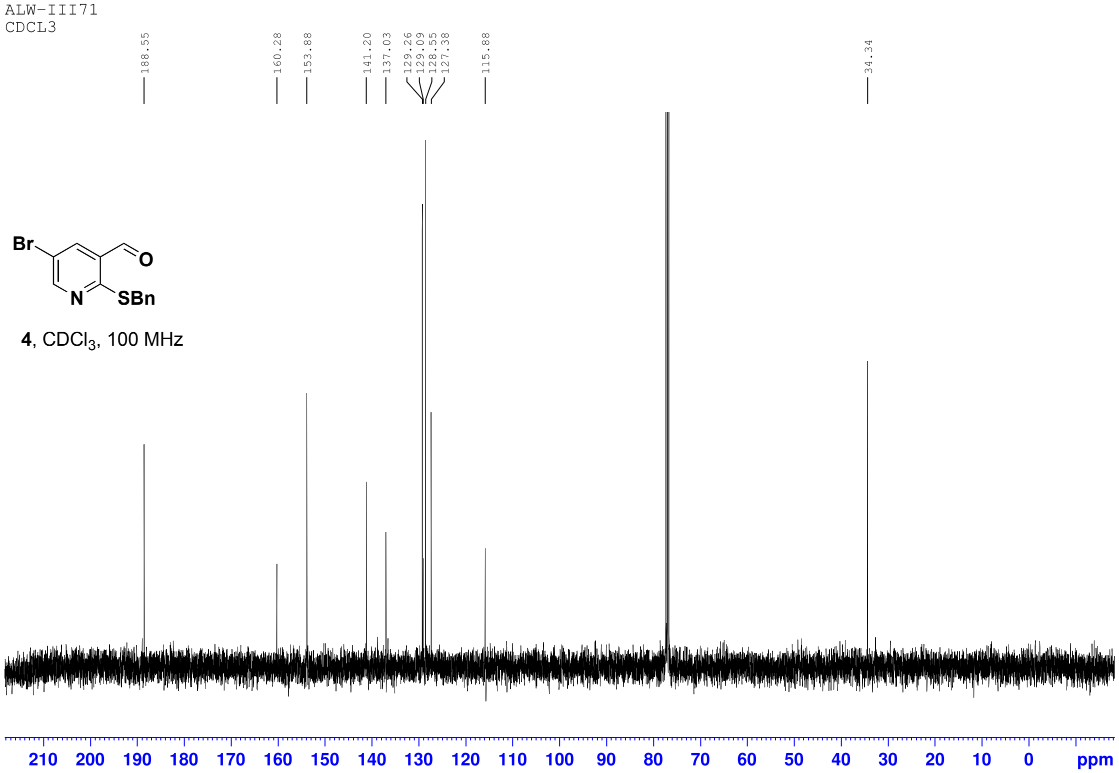

Figure S6. WSA 264 ^1H and ^{13}C NMR spectra.

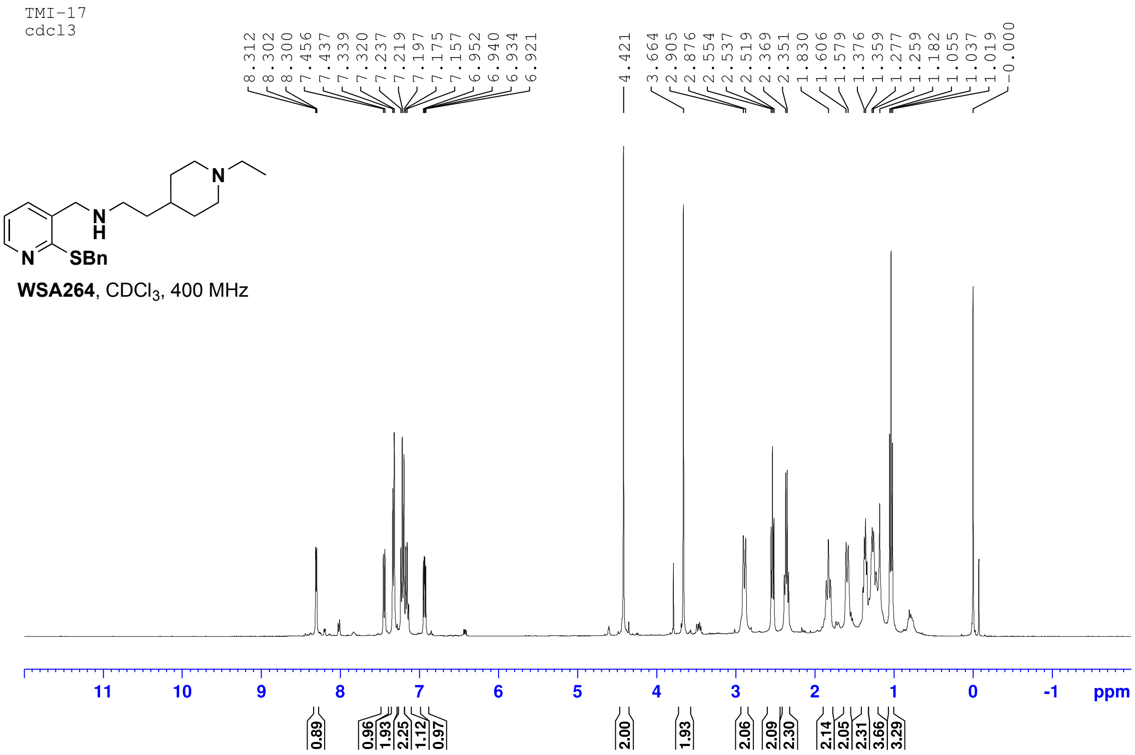

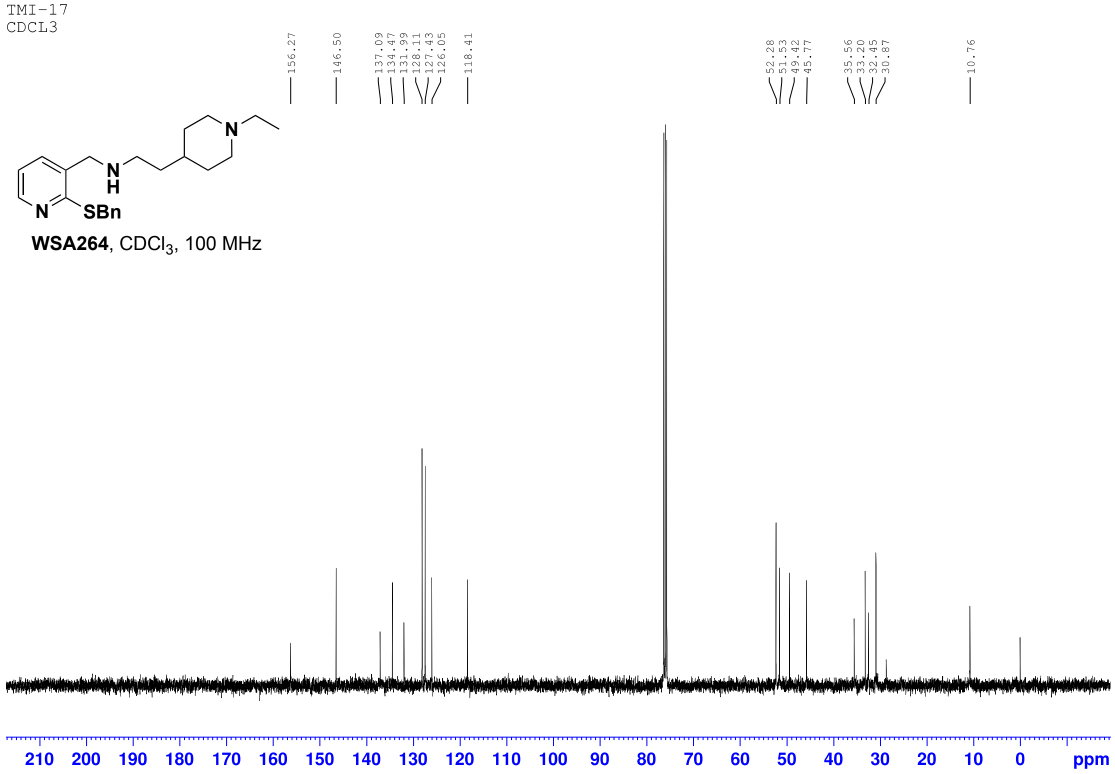

Figure S7. WSA 265 ^{1}H and ^{13}C NMR spectra.

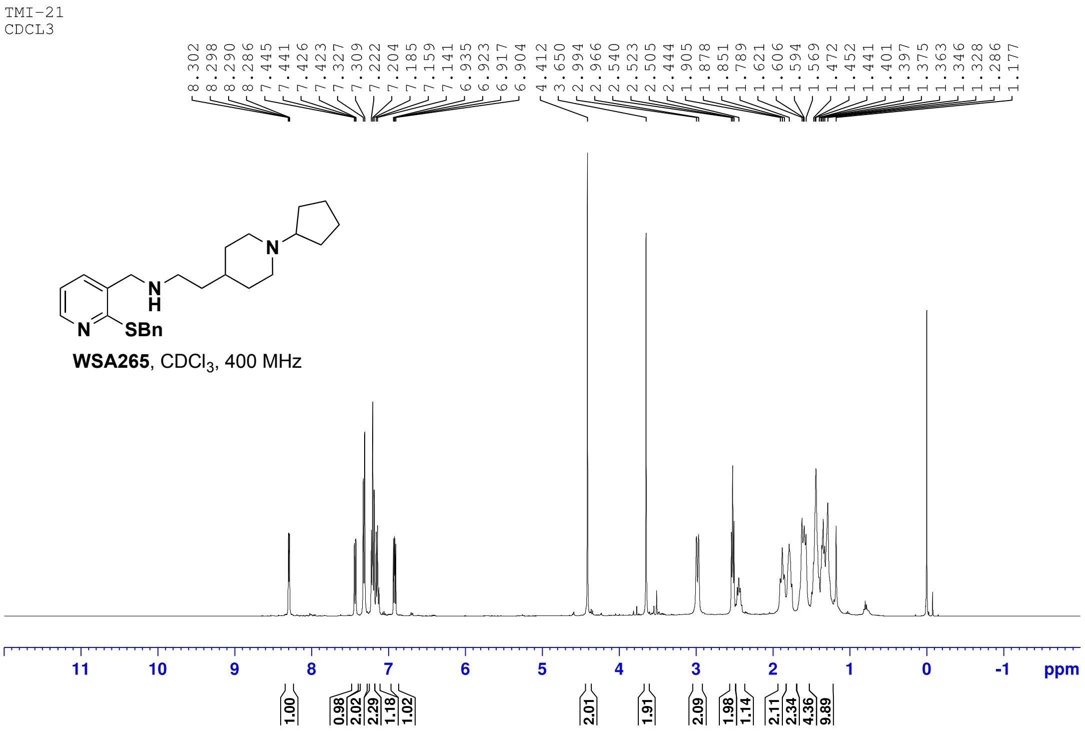

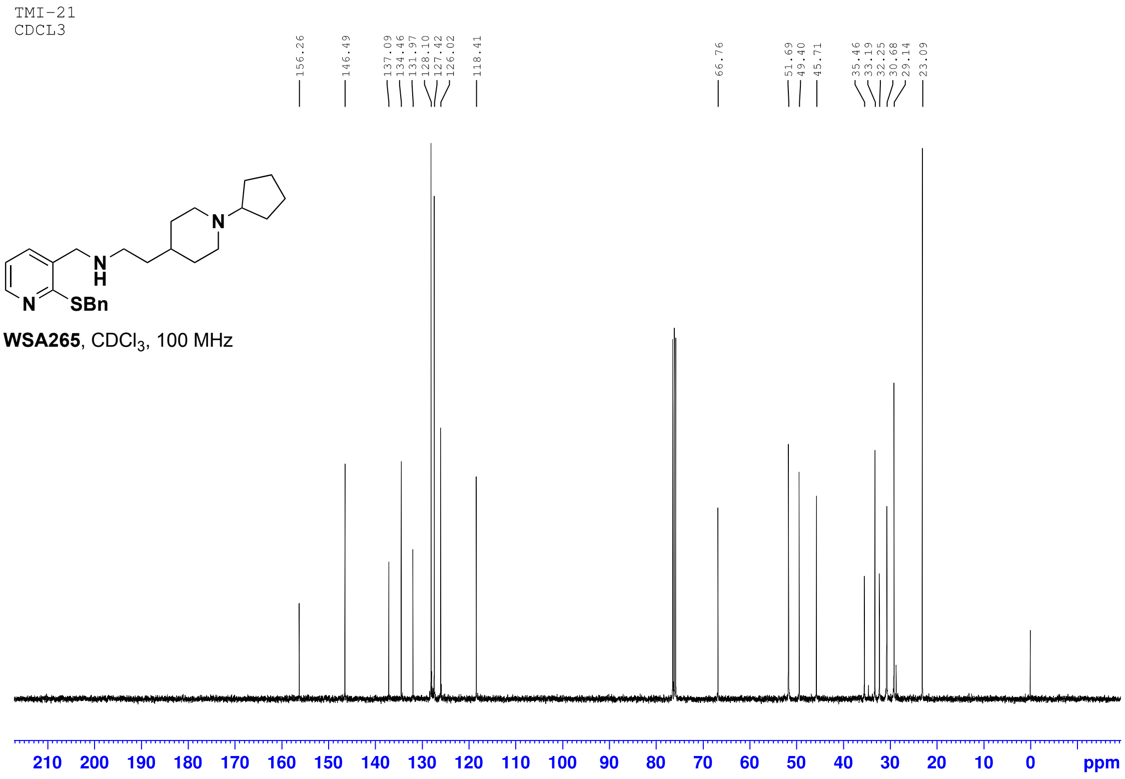

Figure S8. WSA 267 ^{1}H and ^{13}C NMR spectra.

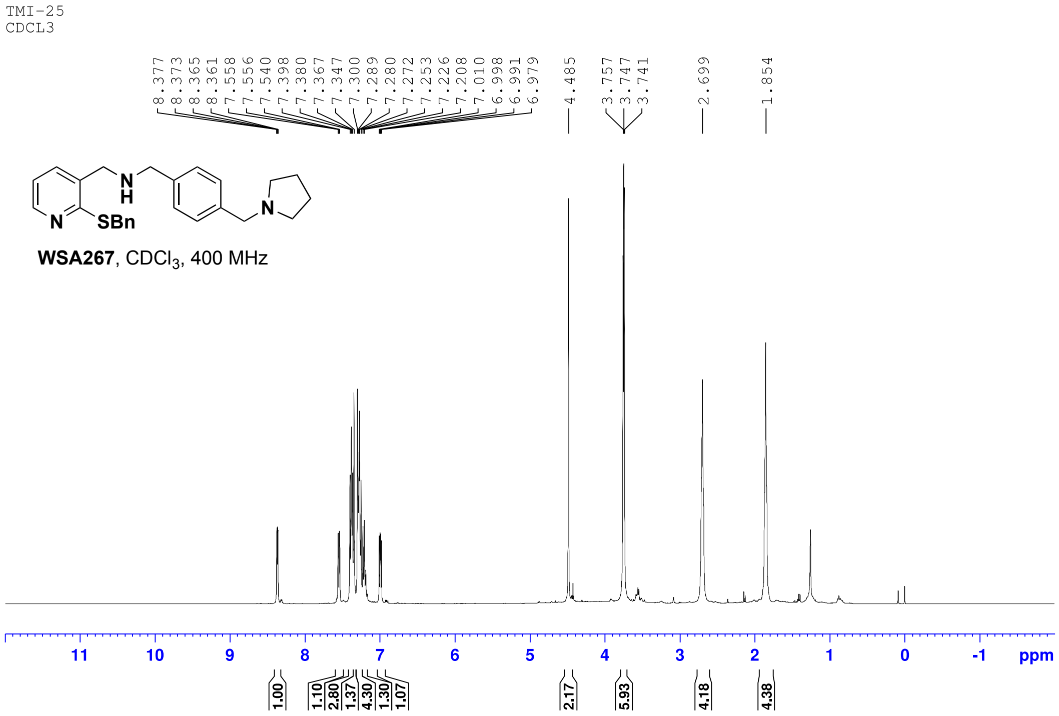

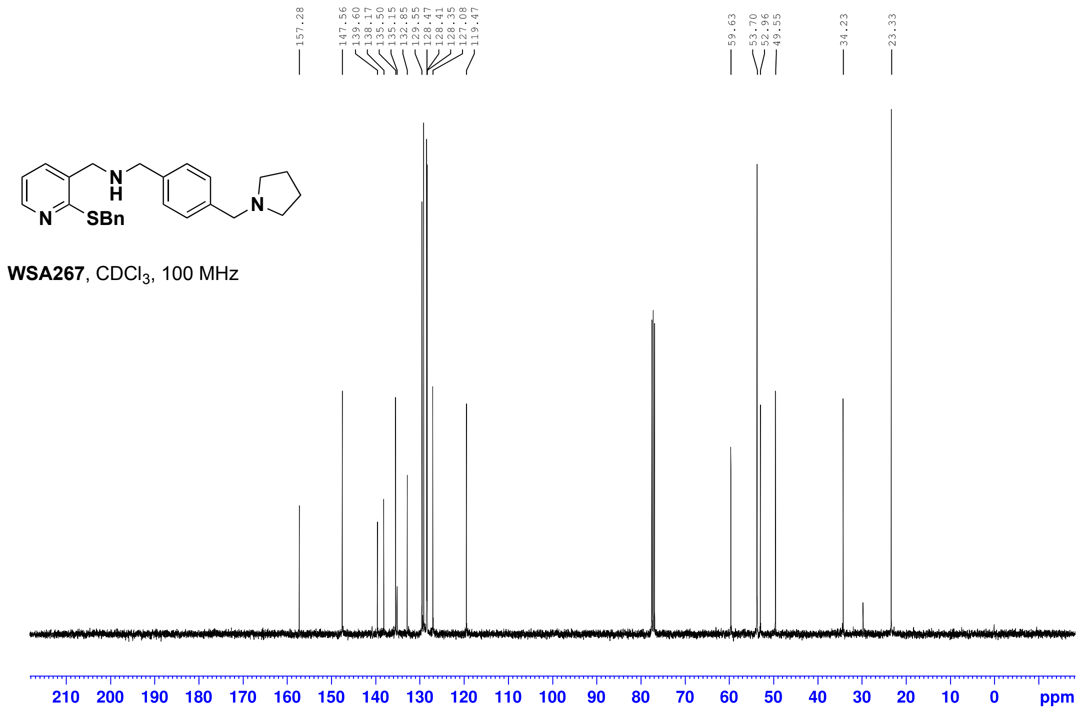

Figure S9. WSA 268 ^{1}H and ^{13}C NMR spectra.

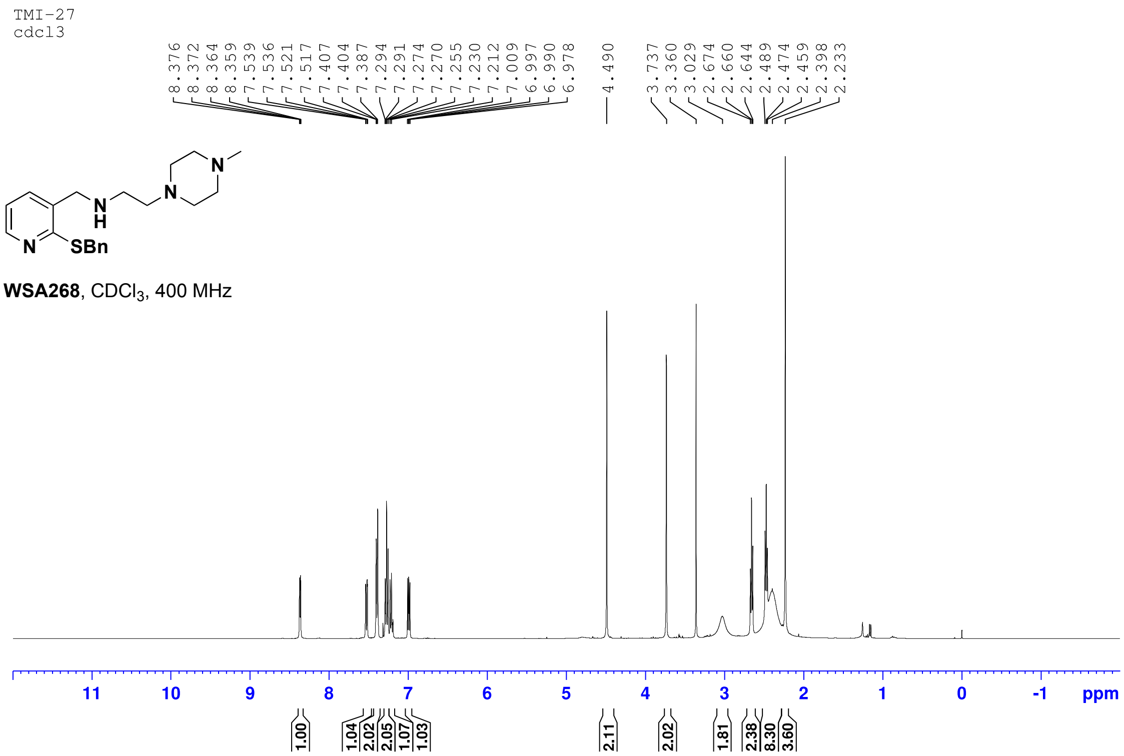

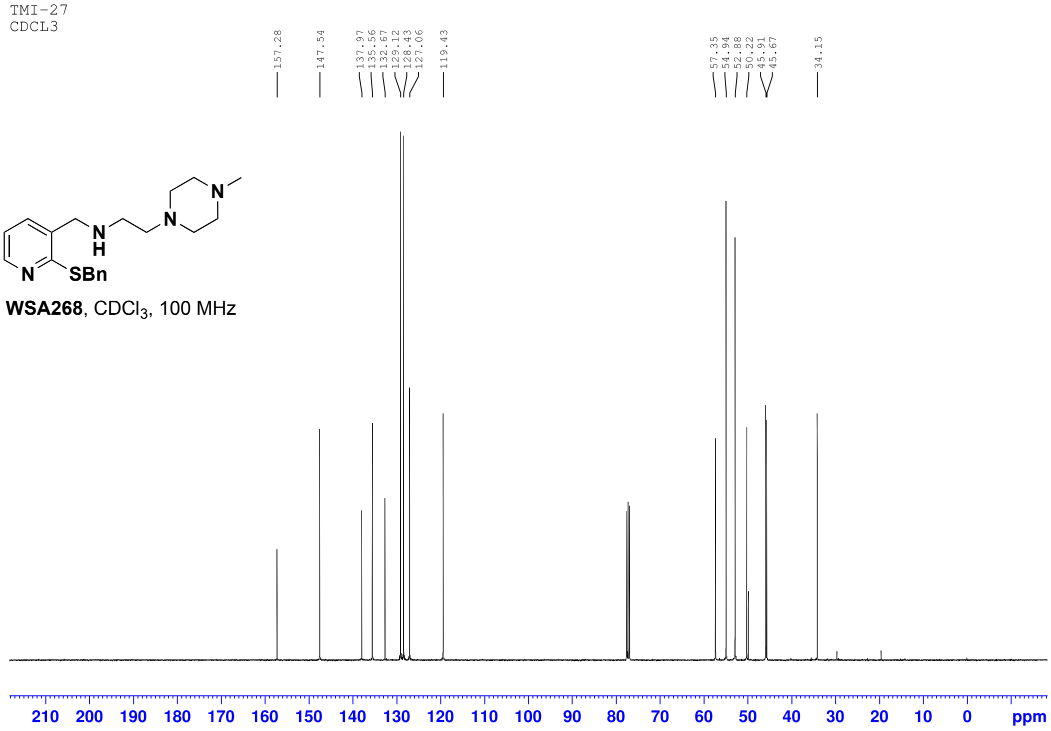

Figure S10. WSA 272 ^{1}H and ^{13}C NMR spectra.

TM-I51 cdcl3

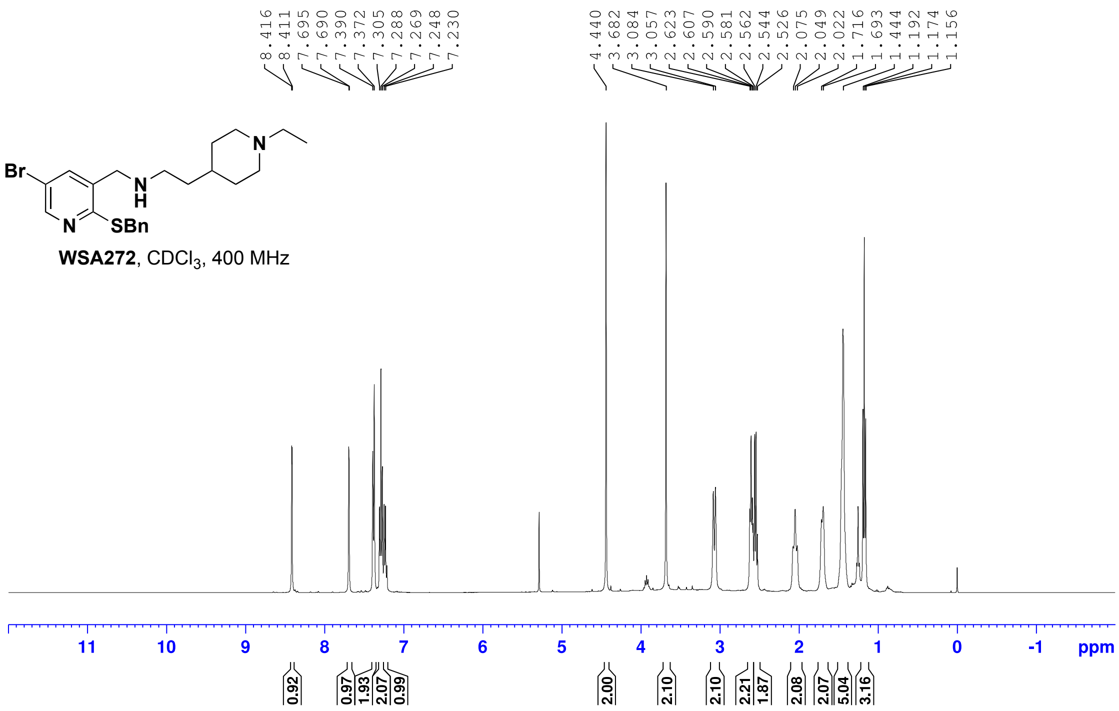

TM-I51 cdcl3

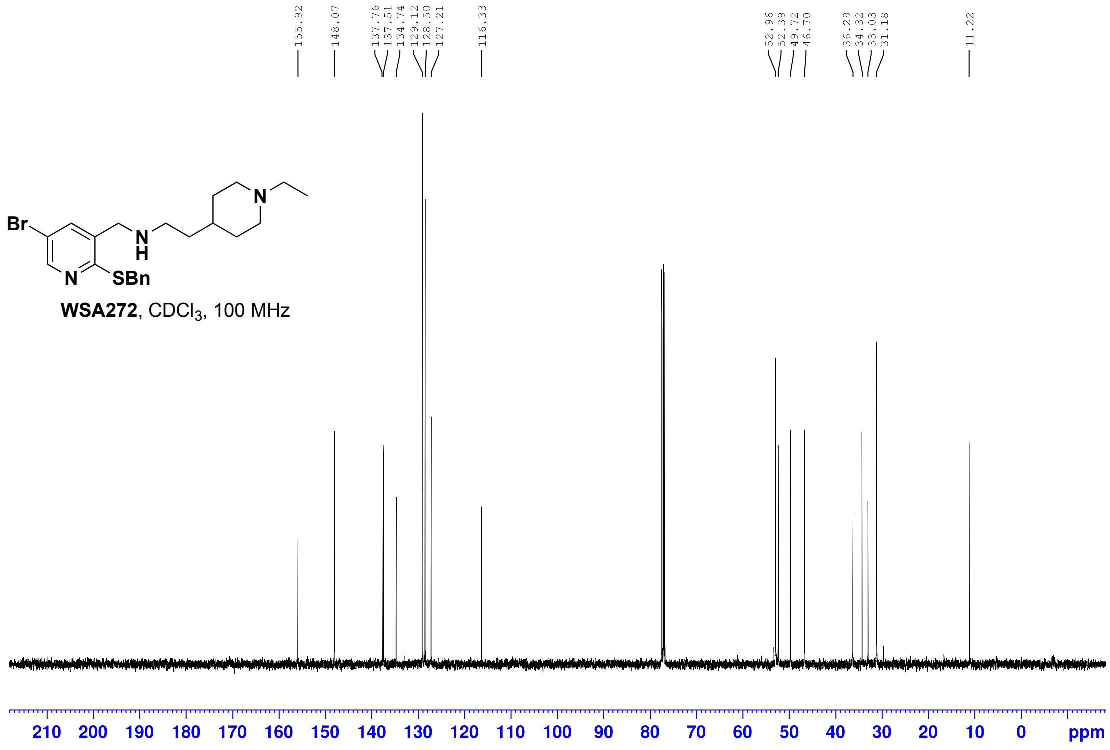

Figure S11. WSA 271 ^{1}H and ^{13}C NMR spectra.

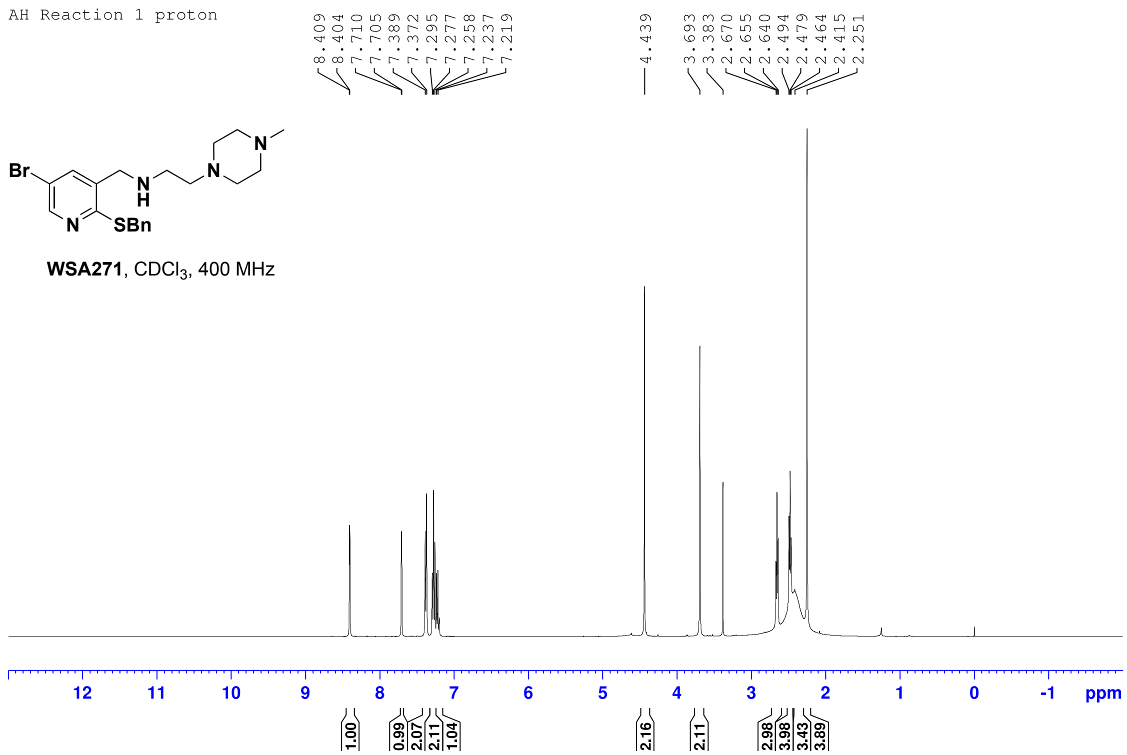

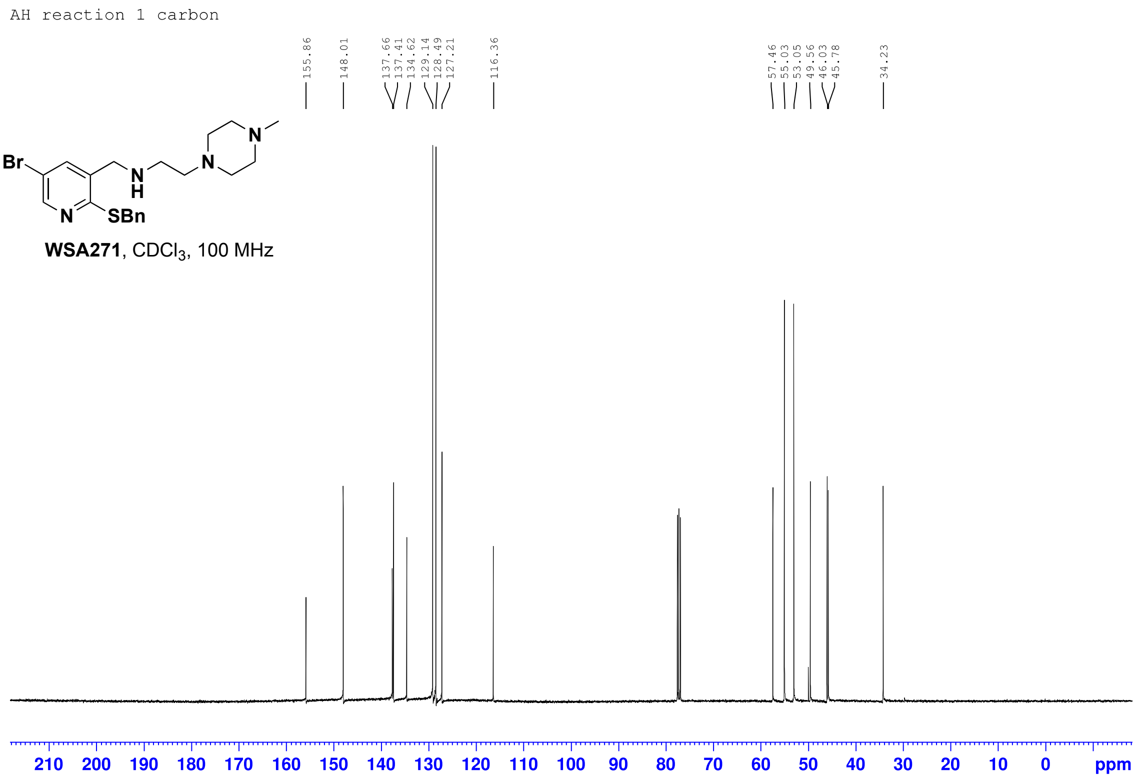

Figure S12. WSA 301 ^{1}H and ^{13}C NMR spectra.

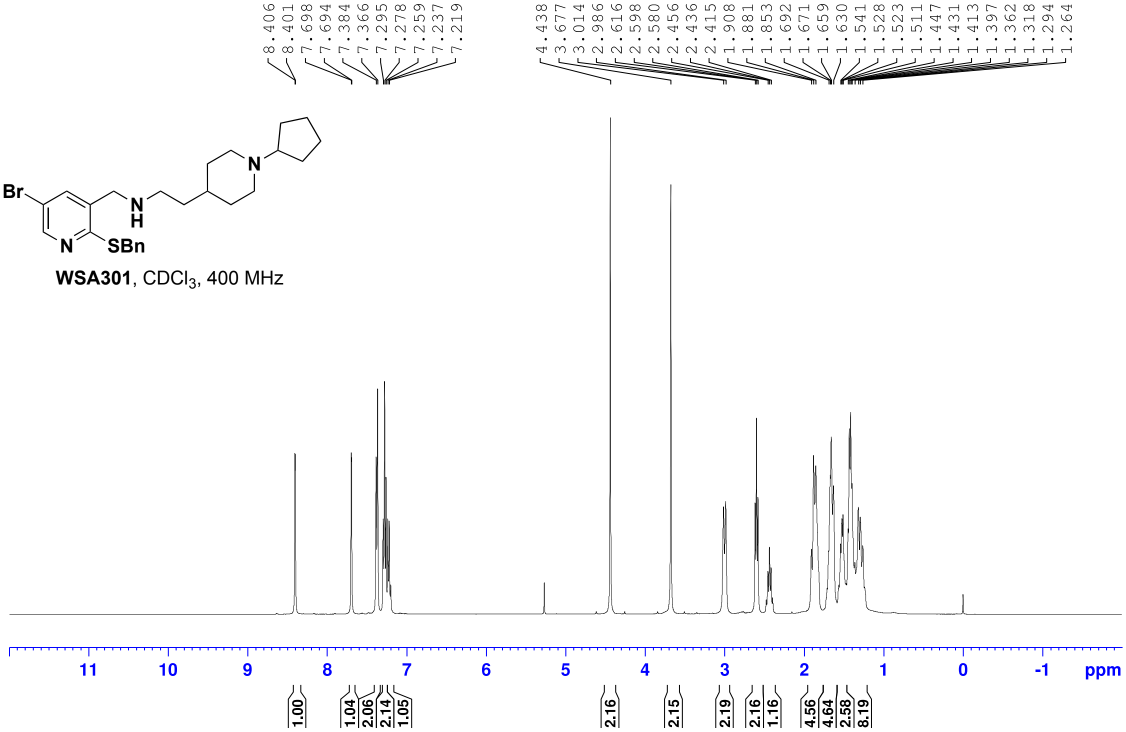

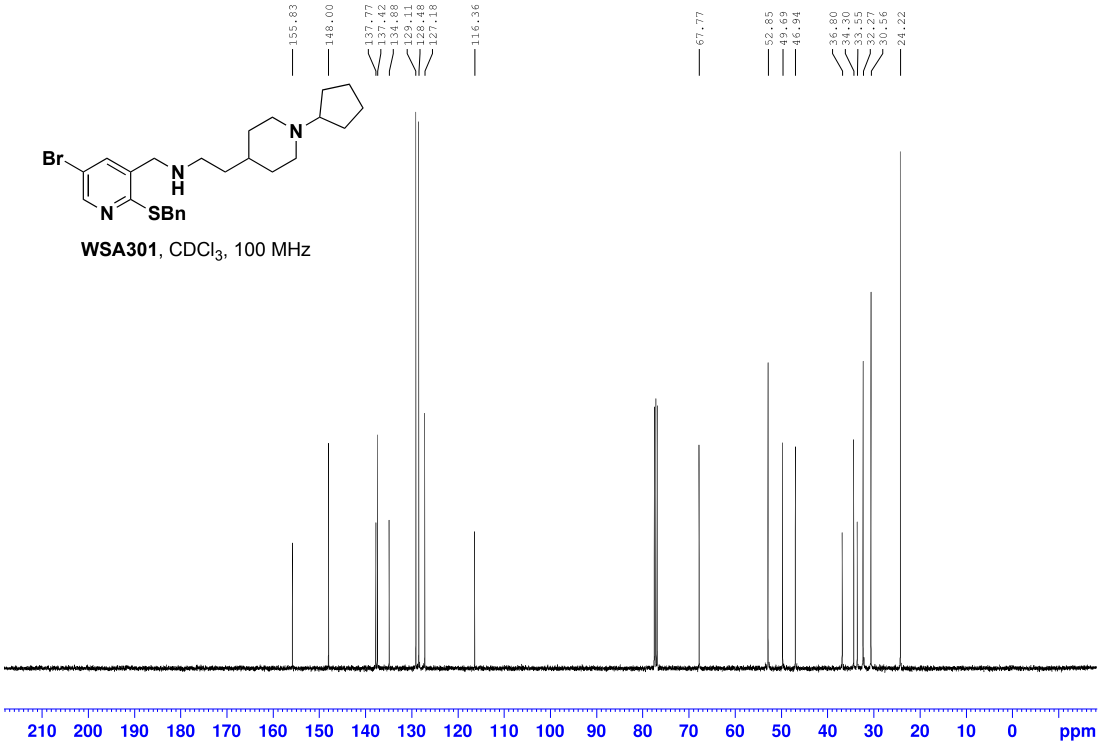

Figure S13. WSA 293 ^{1}H and ^{13}C NMR spectra.

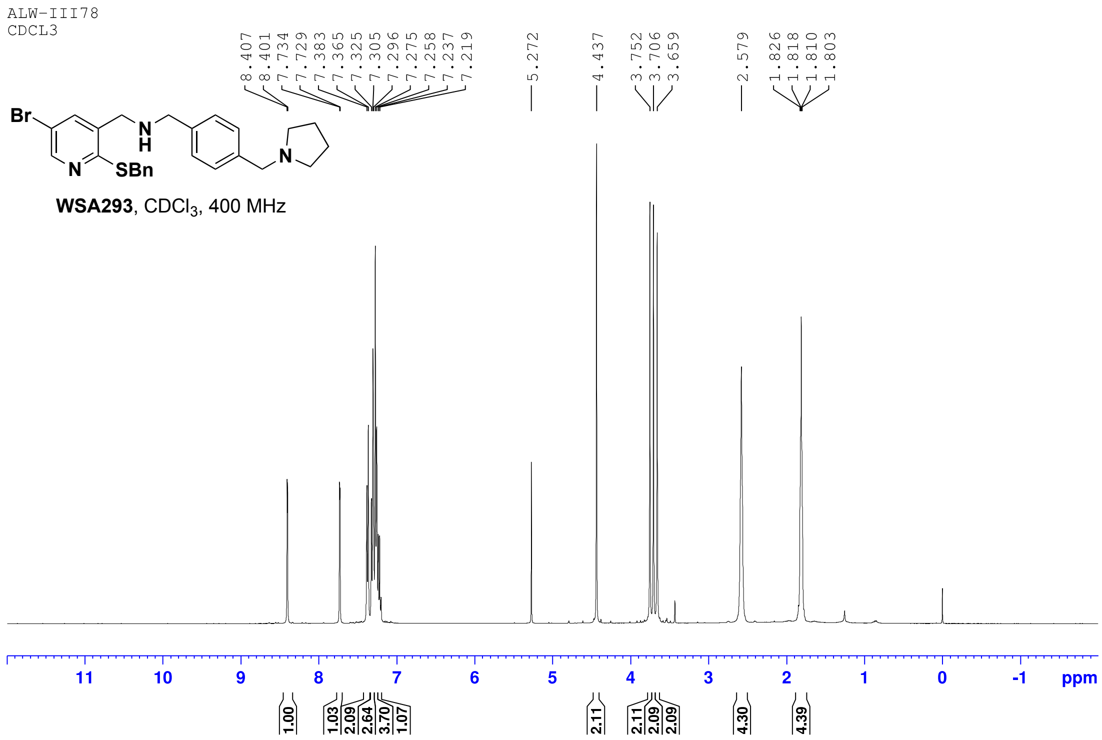

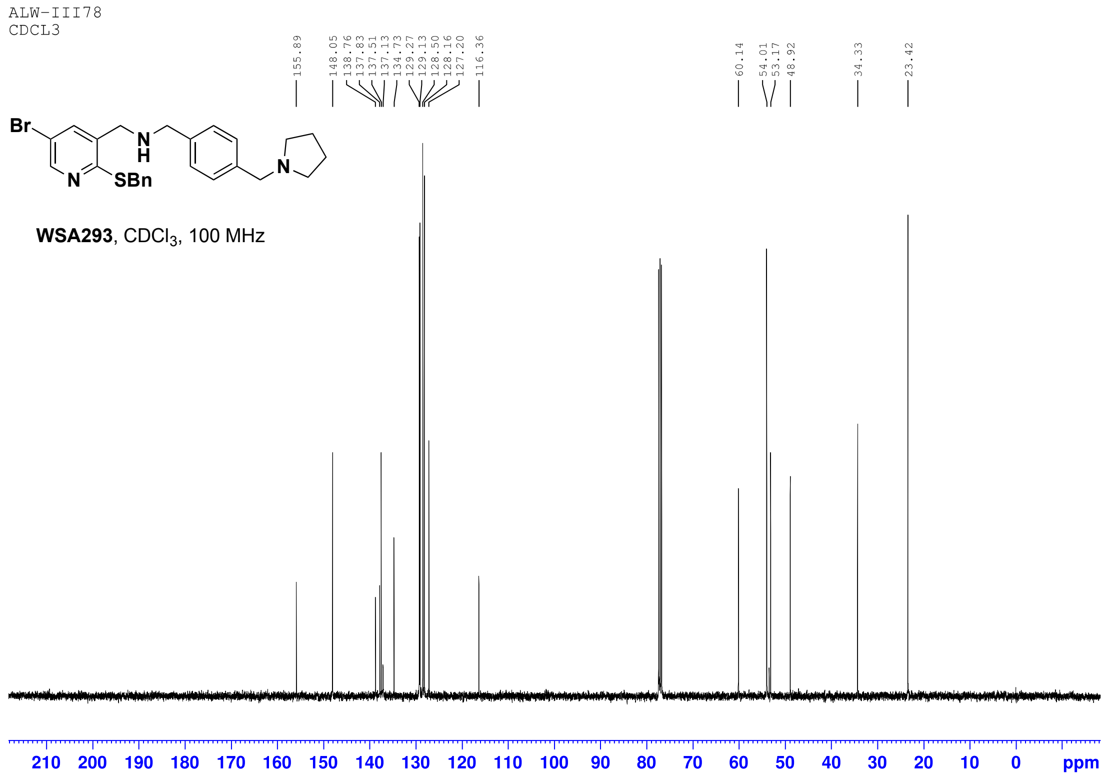

Figure S14. WSA 277 ^{1}H and ^{13}C NMR spectra.

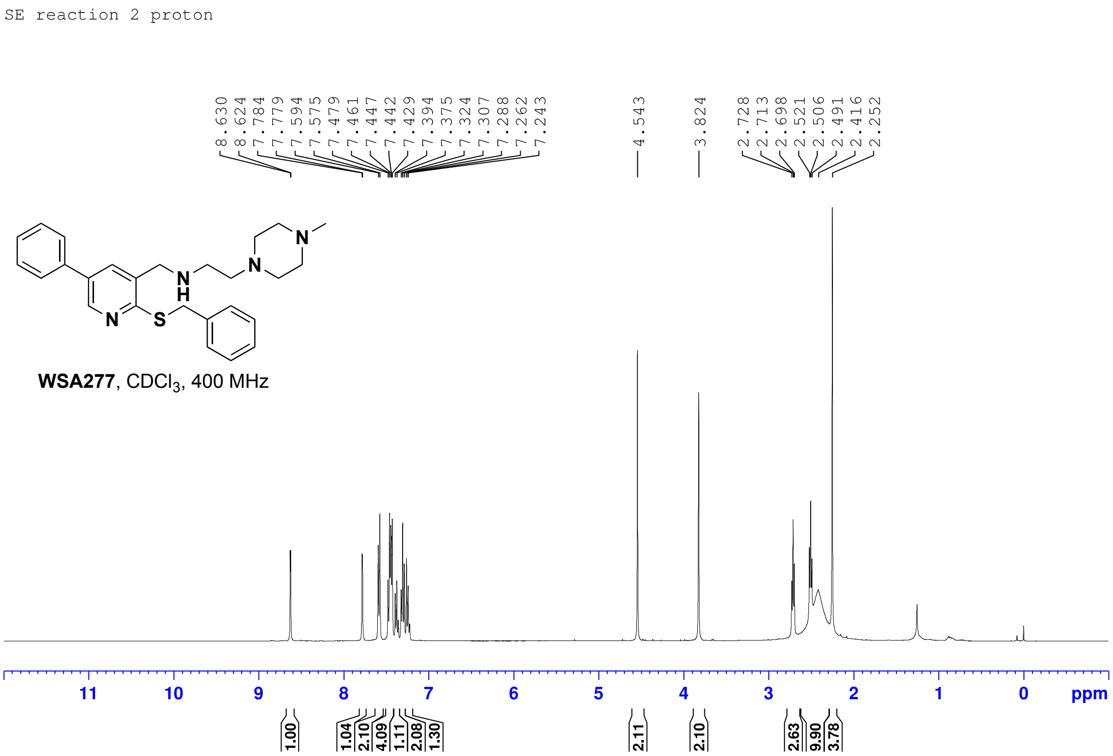

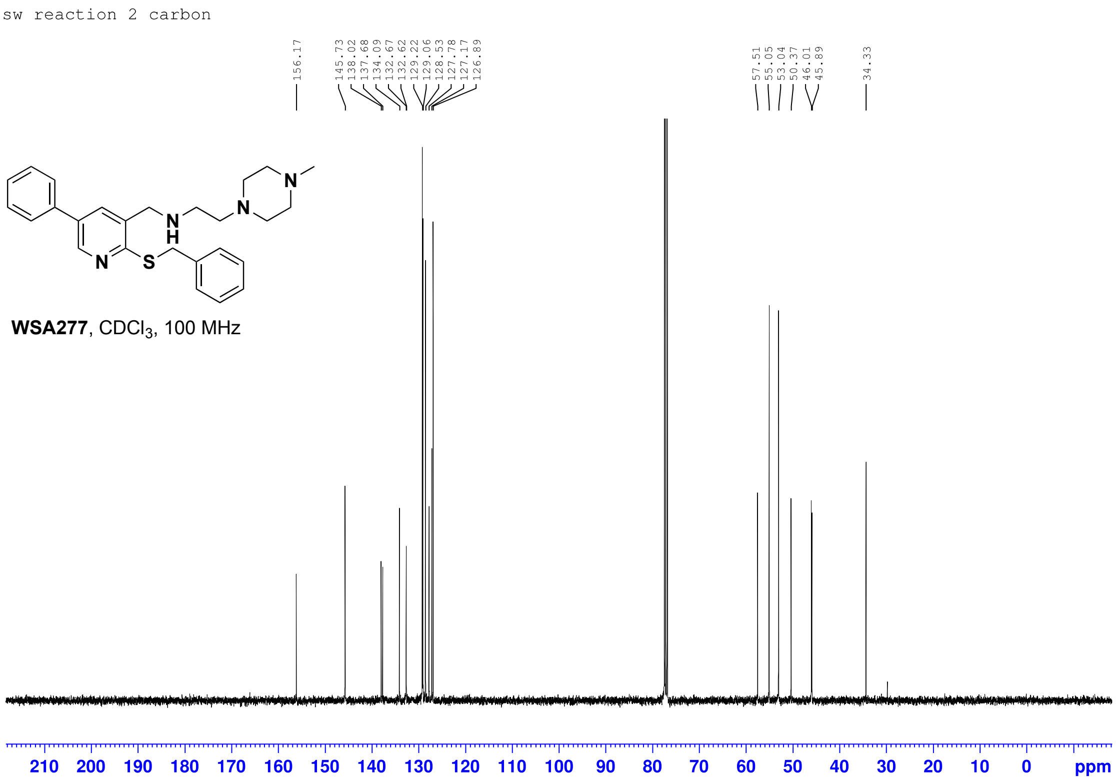

Figure S15. WSA 280 ^{1}H and ^{13}C NMR spectra.

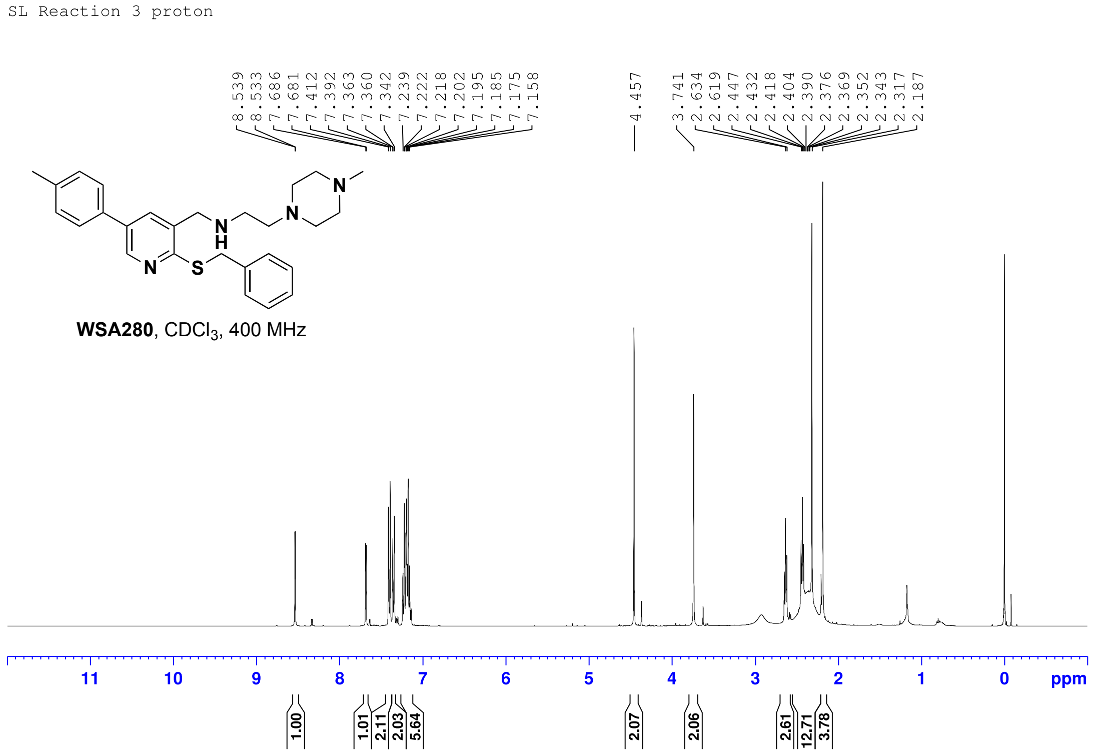

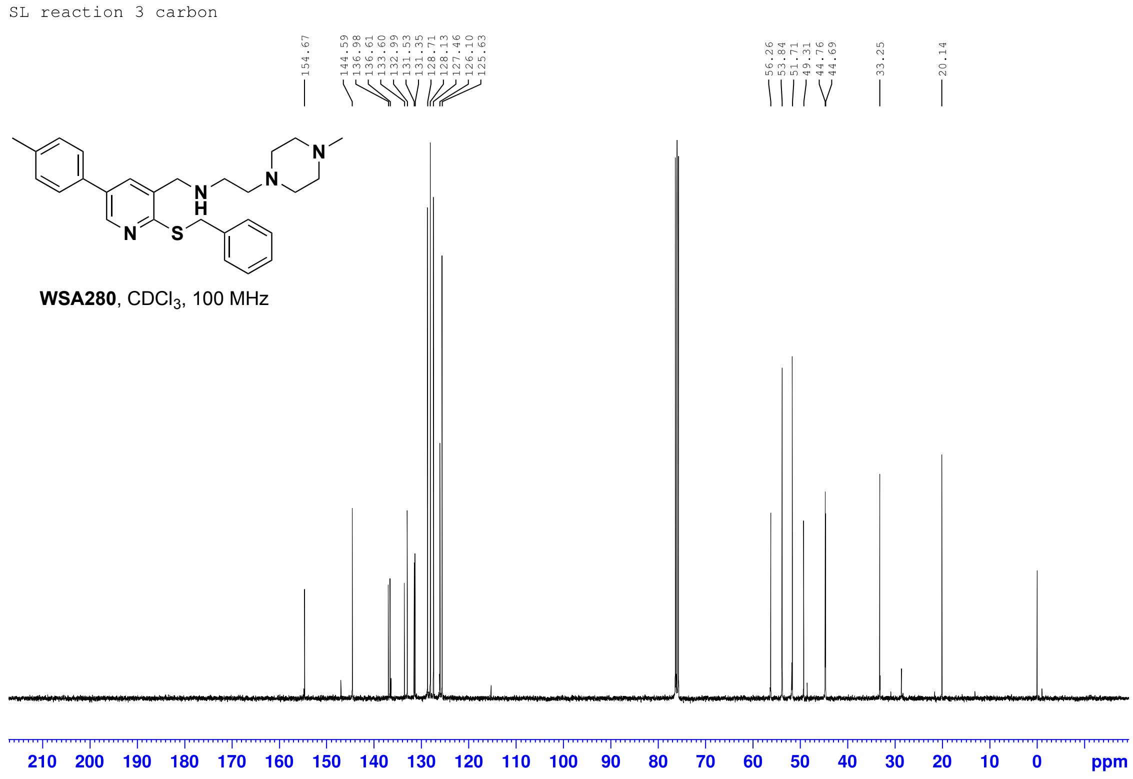

Figure S16. WSA 278 ^{1}H and ^{13}C NMR spectra.

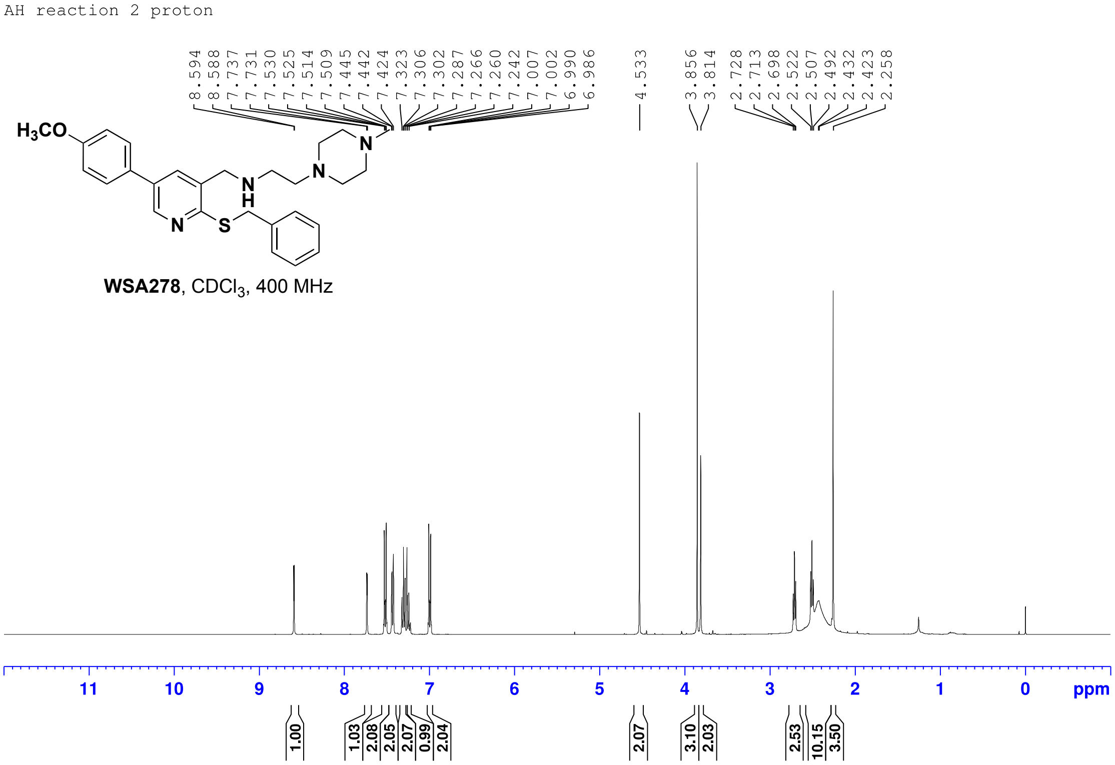

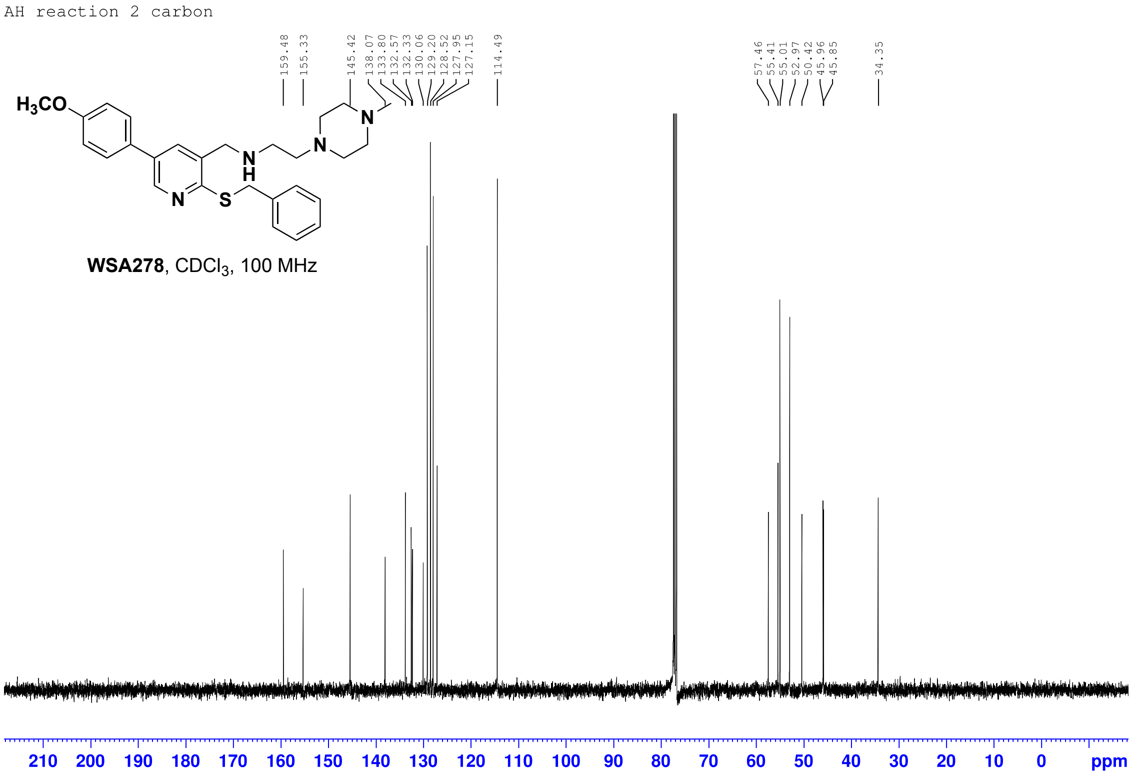

Figure S17. WSA 273 ^{1}H and ^{13}C NMR spectra.

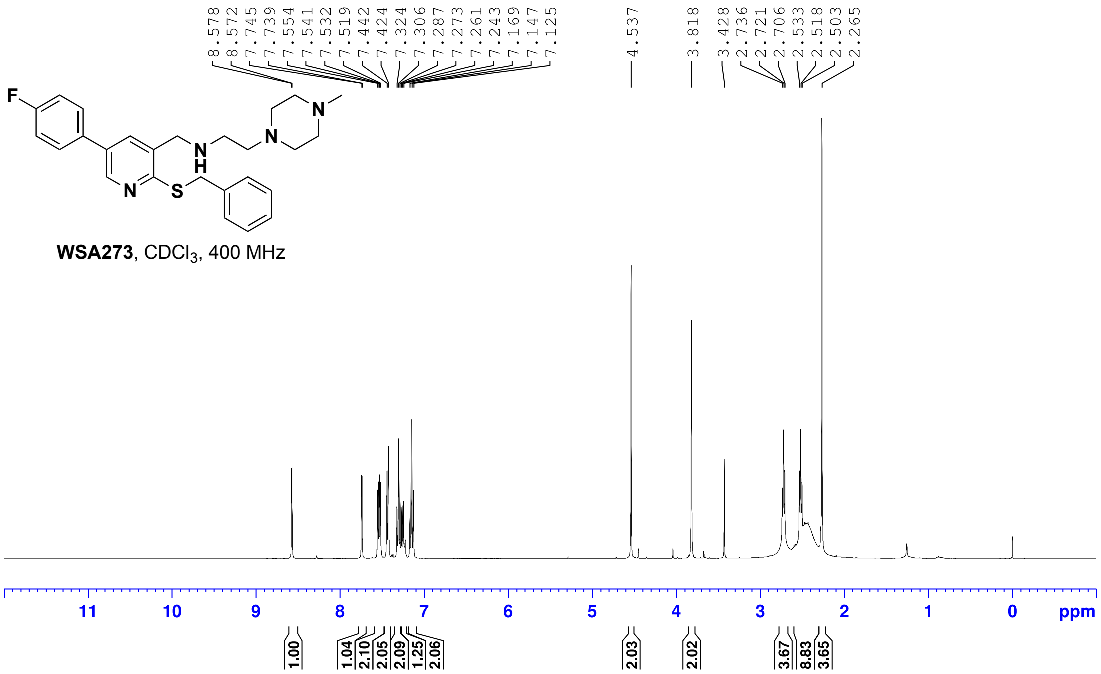

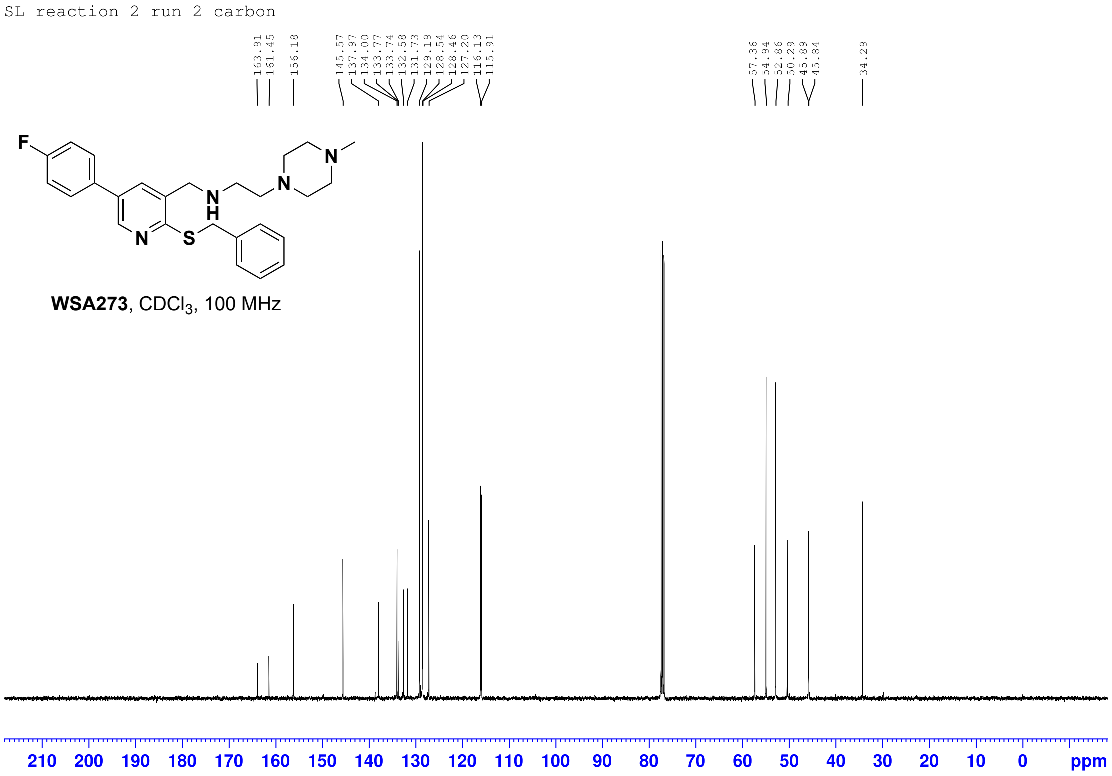

Figure S18. WSA 274 ^{1}H and ^{13}C NMR spectra.

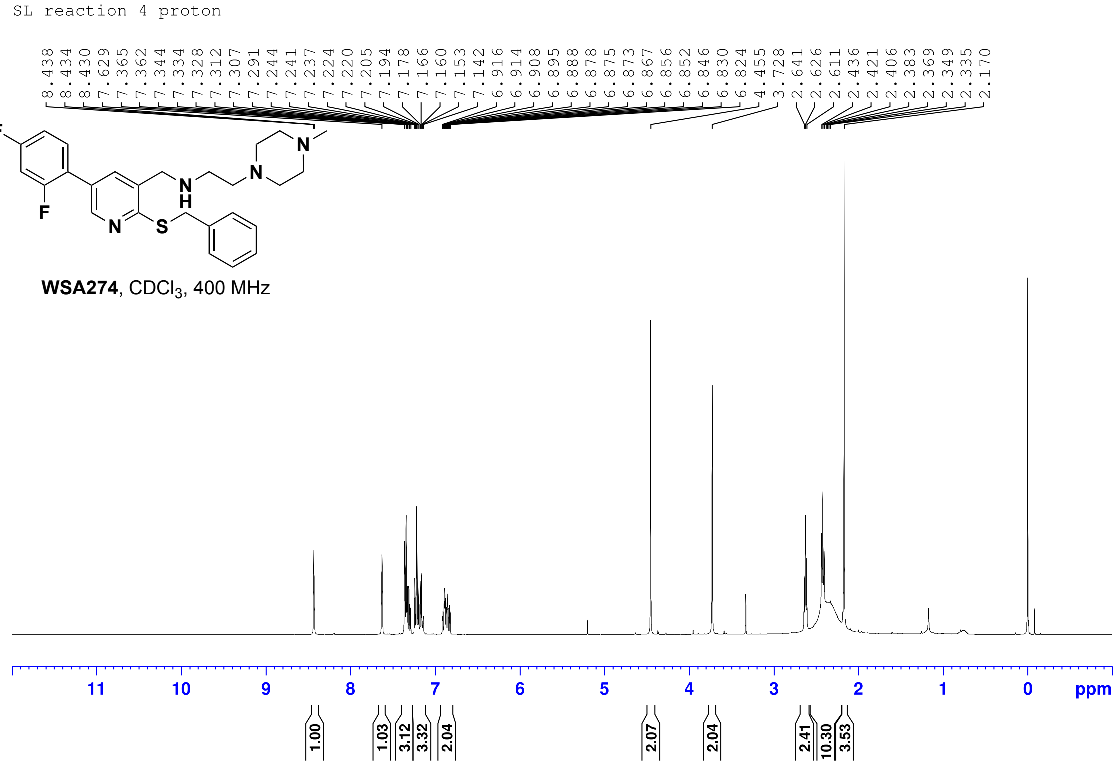

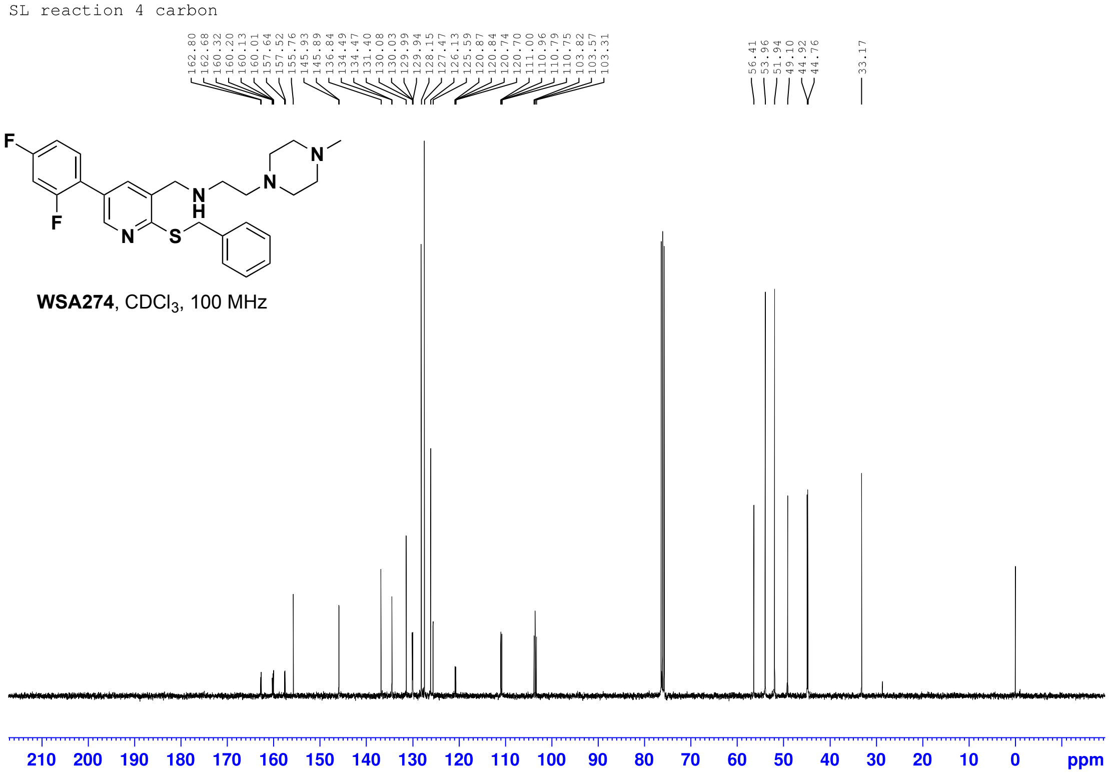

Figure S19. WSA 275 ^{1}H and ^{13}C NMR spectra.

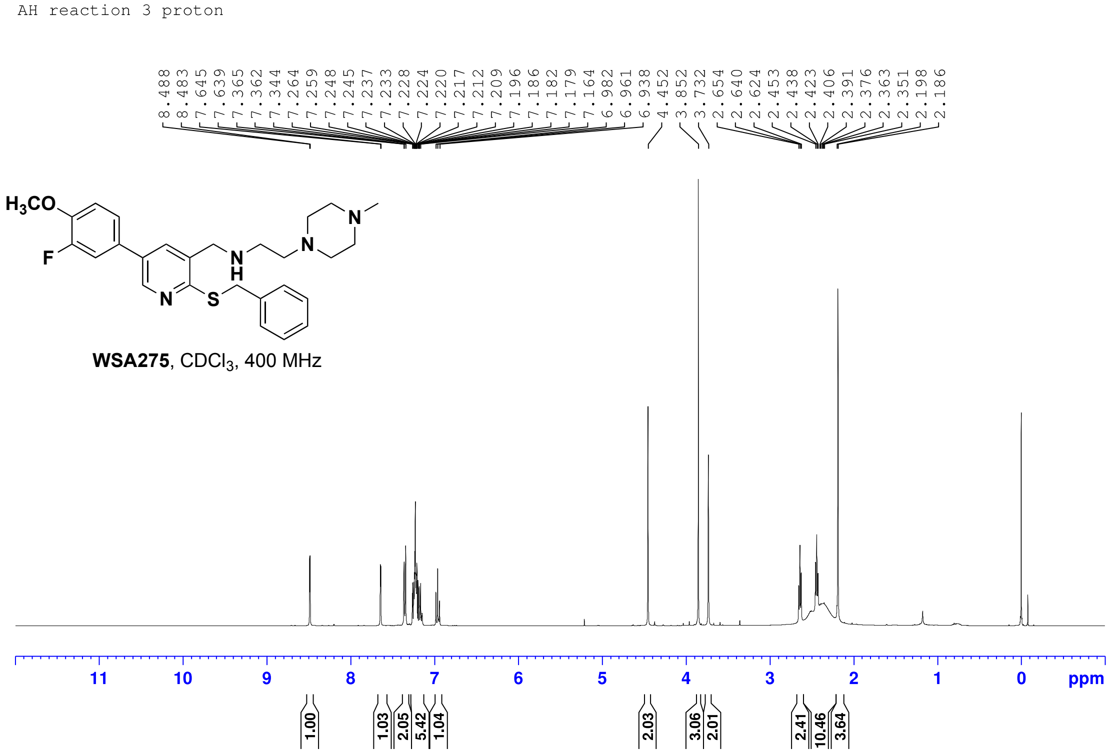

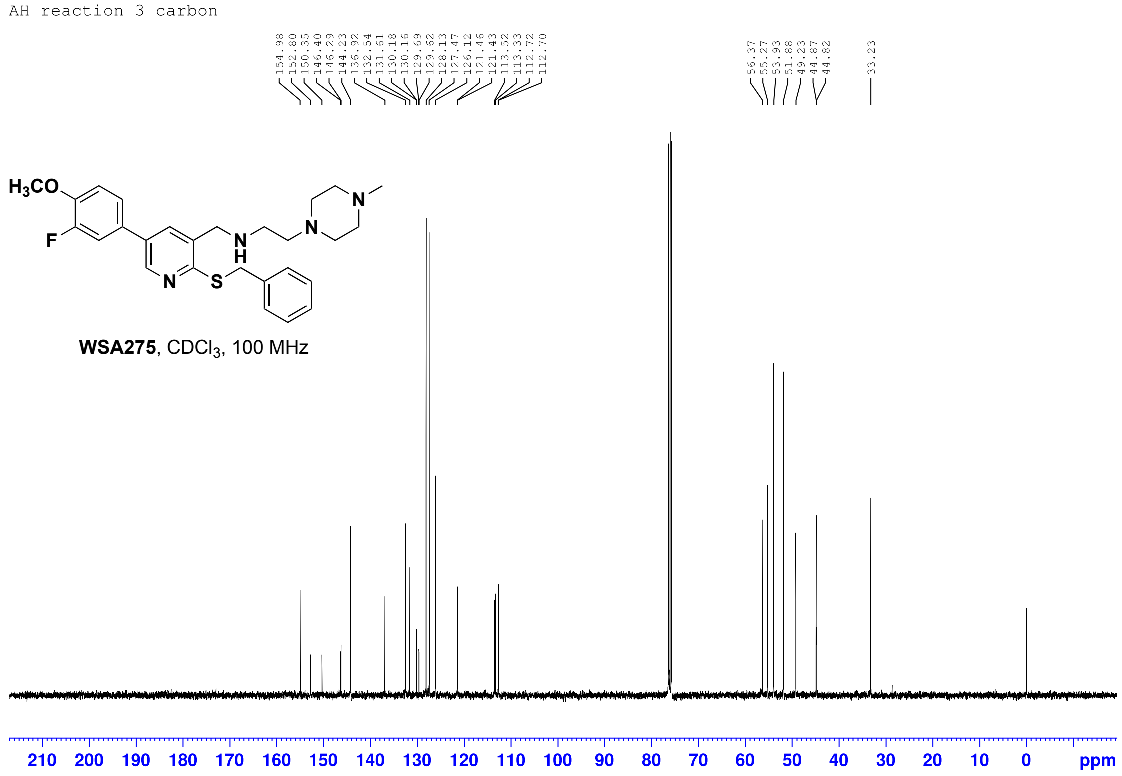

Figure S20. WSA 276 ^{1}H and ^{13}C NMR spectra.

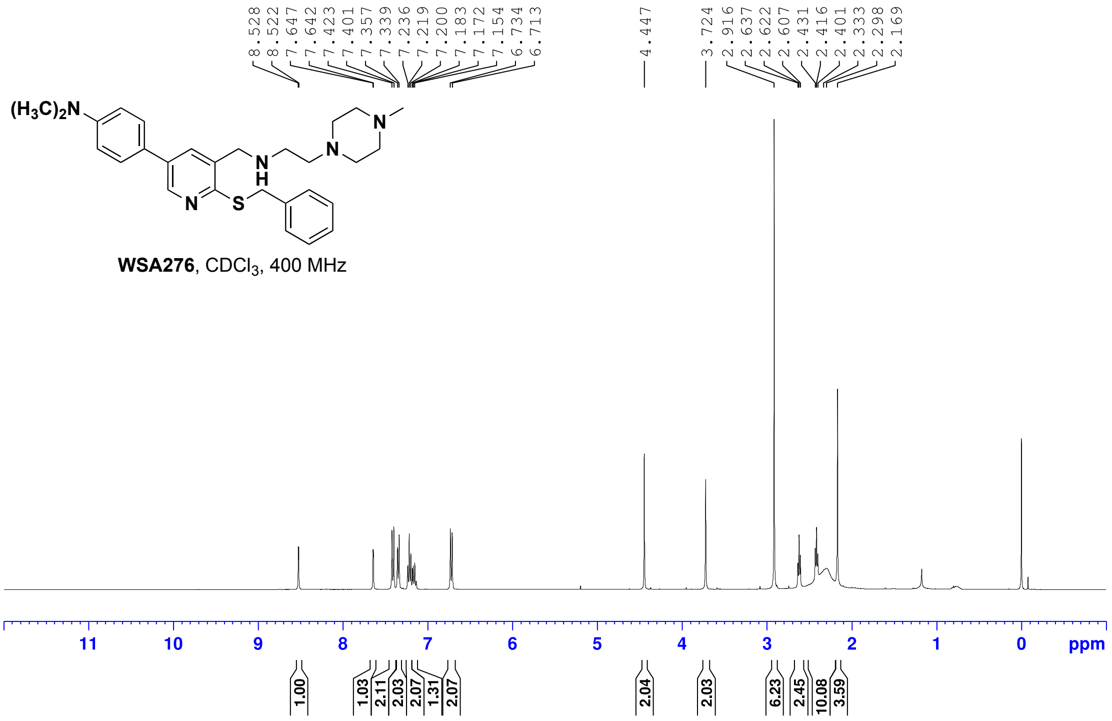

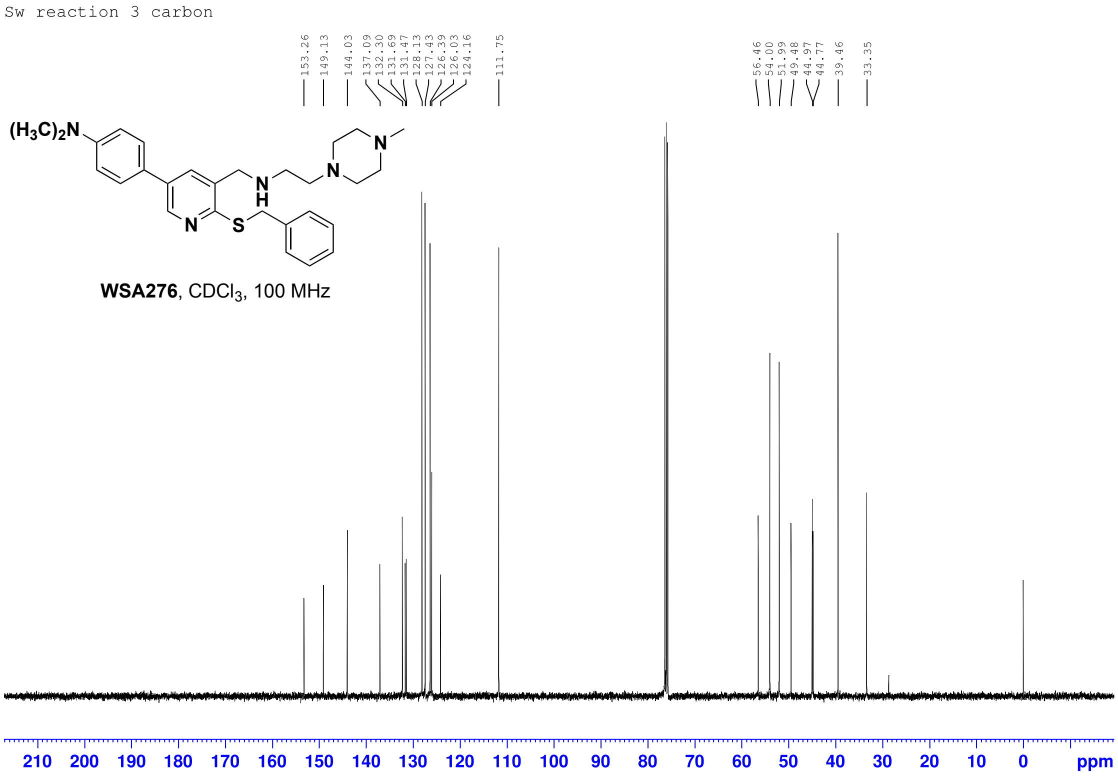

Figure S21. WSA 302 ^{1}H and ^{13}C NMR spectra.

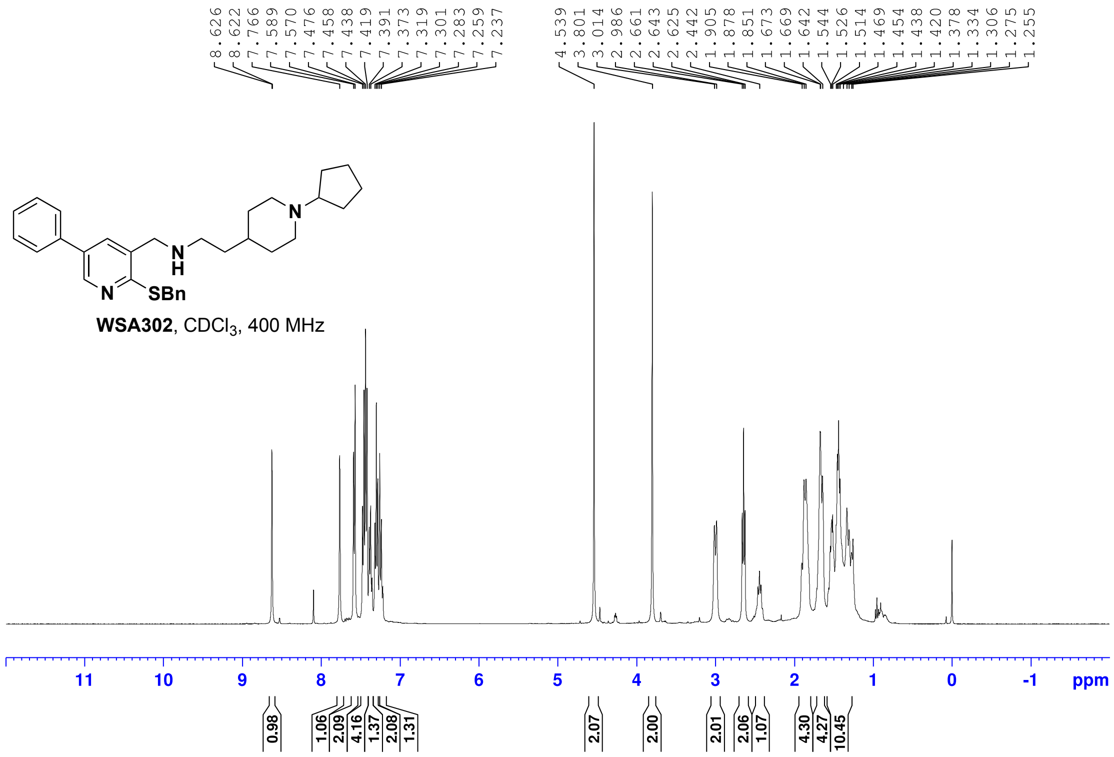
8.626
8.622
7.766
7.589
7.570
7.476
7.458
7.438
7.419
7.391
7.373
7.319
7.301
7.283
7.259
7.237
4.539
3.801
3.014
2.986
2.661
2.643
2.625
2.442
1.905
1.878
1.851
1.673
1.669
1.642
1.544
1.526
1.514
1.469
1.454
1.438
1.420
1.378
1.334
1.306
1.275
1.255
N
H
N
N
SBn
WSA302, $CDCl_3$, 400 MHz
11
10
9
8
7
6
5
4
3
2
1
0
-1
ppm
0.98
1.06
2.09
4.16
1.37
2.08
1.31
2.07
2.00
2.01
2.06
1.07
4.30
4.27
10.45

ASI-14, CDCl3

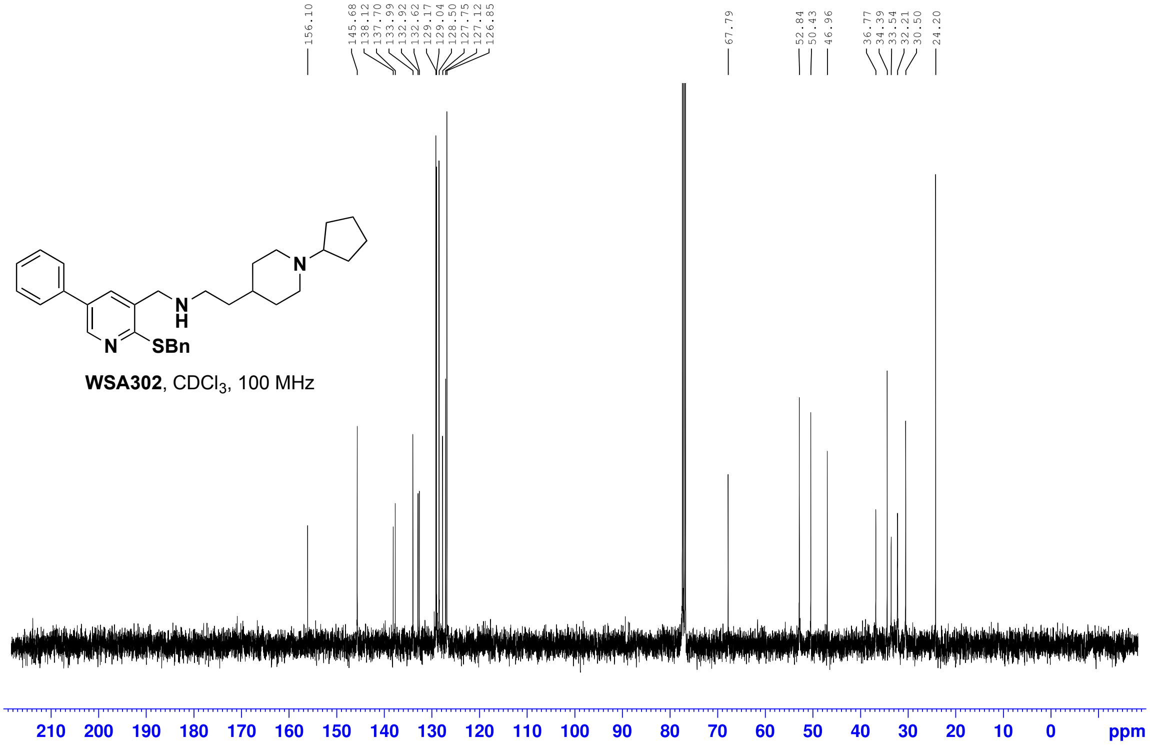
156.10
145.68
138.12
137.70
133.99
132.92
132.62
129.17
129.04
128.50
127.75
127.12
126.85
67.79
52.84
50.43
46.96
36.77
34.39
33.54
32.21
30.50
24.20
N
H
N
N
SBn
WSA302, $CDCl_3$, 100 MHz
210
200
190
180
170
160
150
140
130
120
110
100
90
80
70
60
50
40
30
20
10
0
ppm

Figure S22. WSA 288 ^{1}H and ^{13}C NMR spectra.

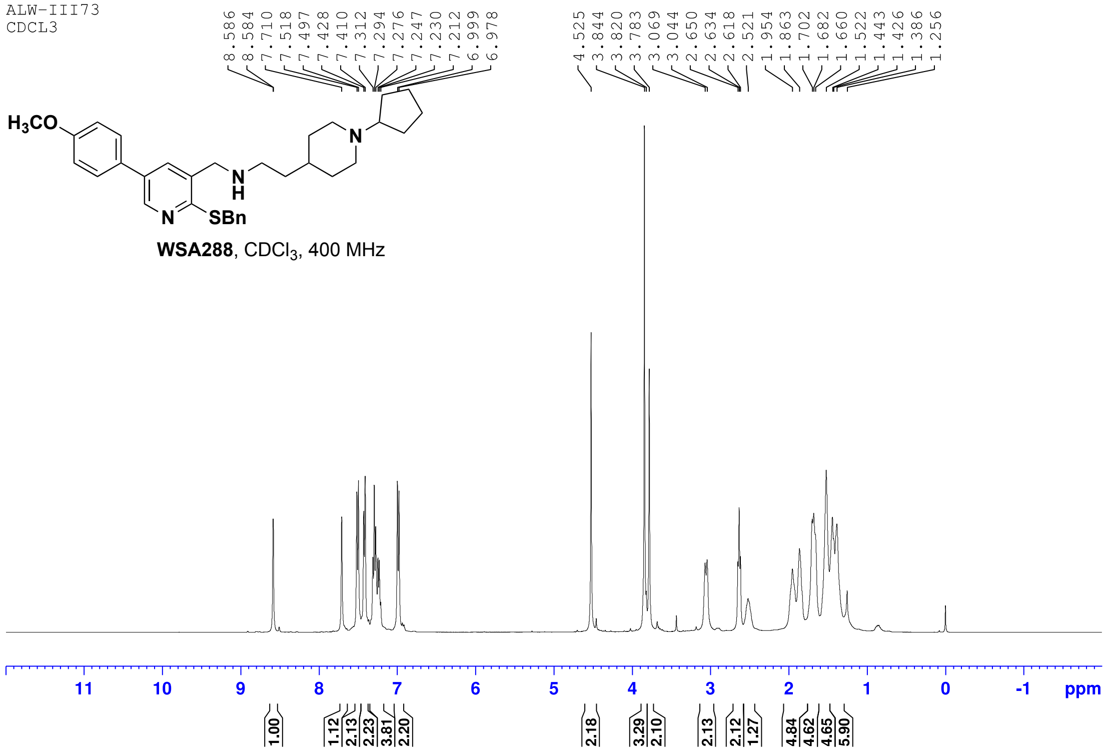

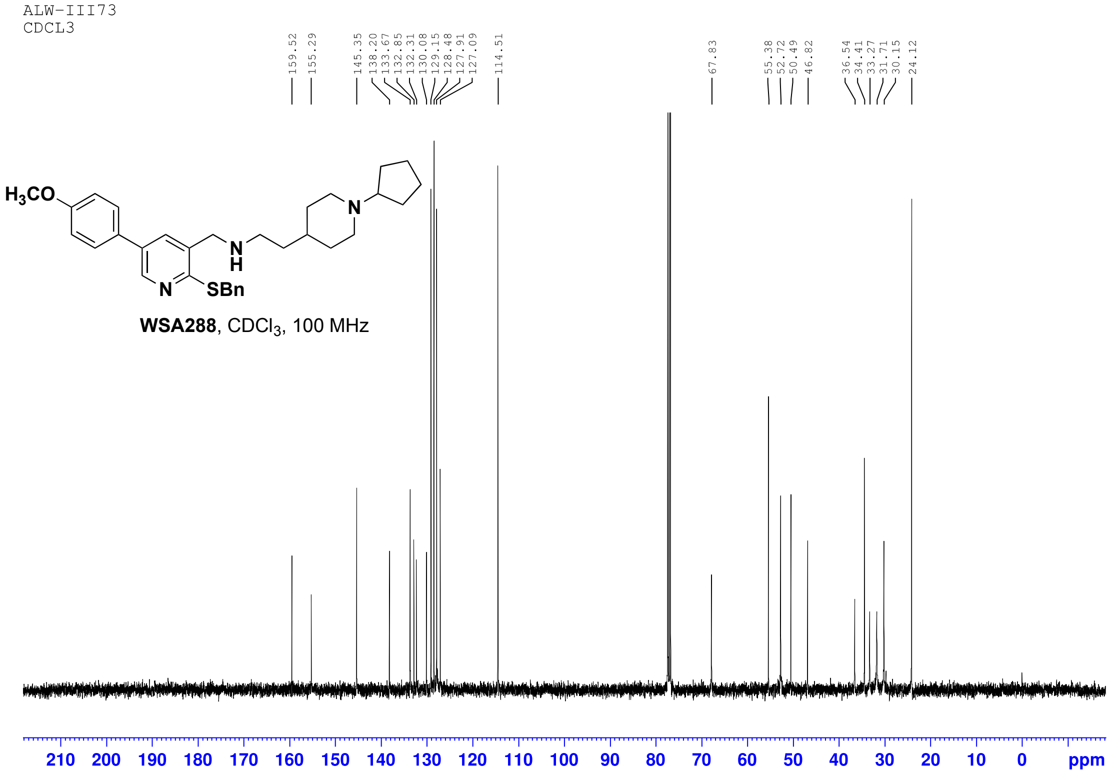

Figure S23. WSA 289 ^{1}H and ^{13}C NMR spectra.

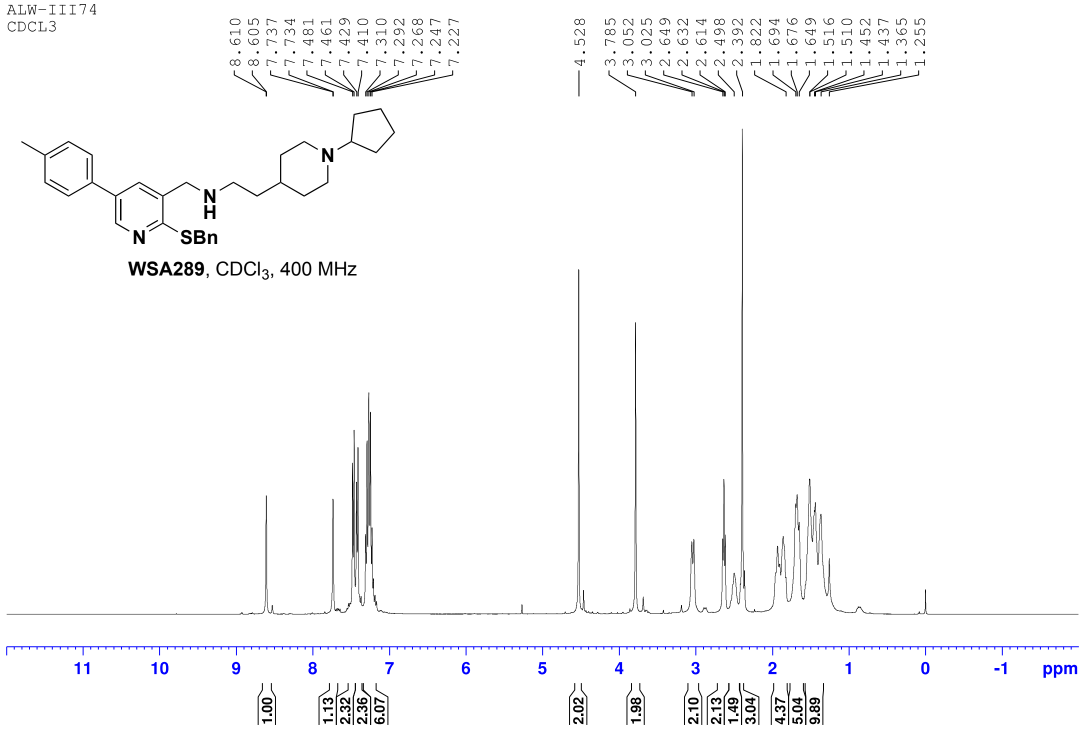

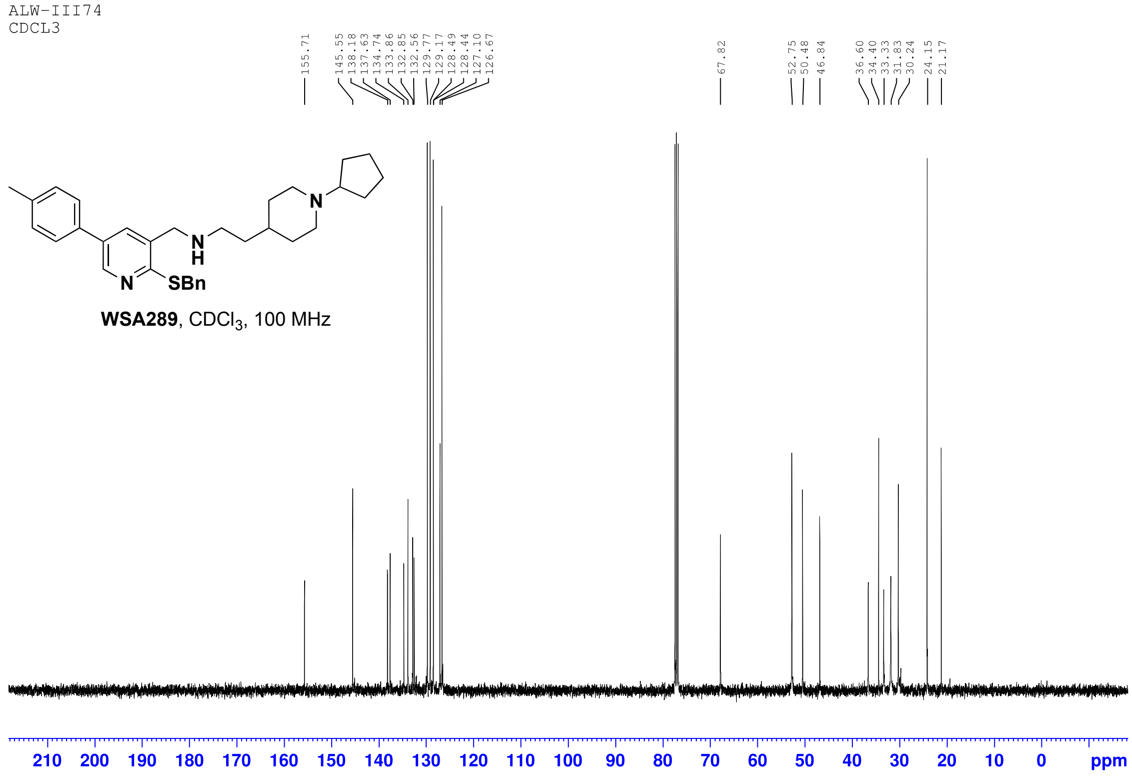

Figure S24. WSA 290 ^{1}H and ^{13}C NMR spectra.

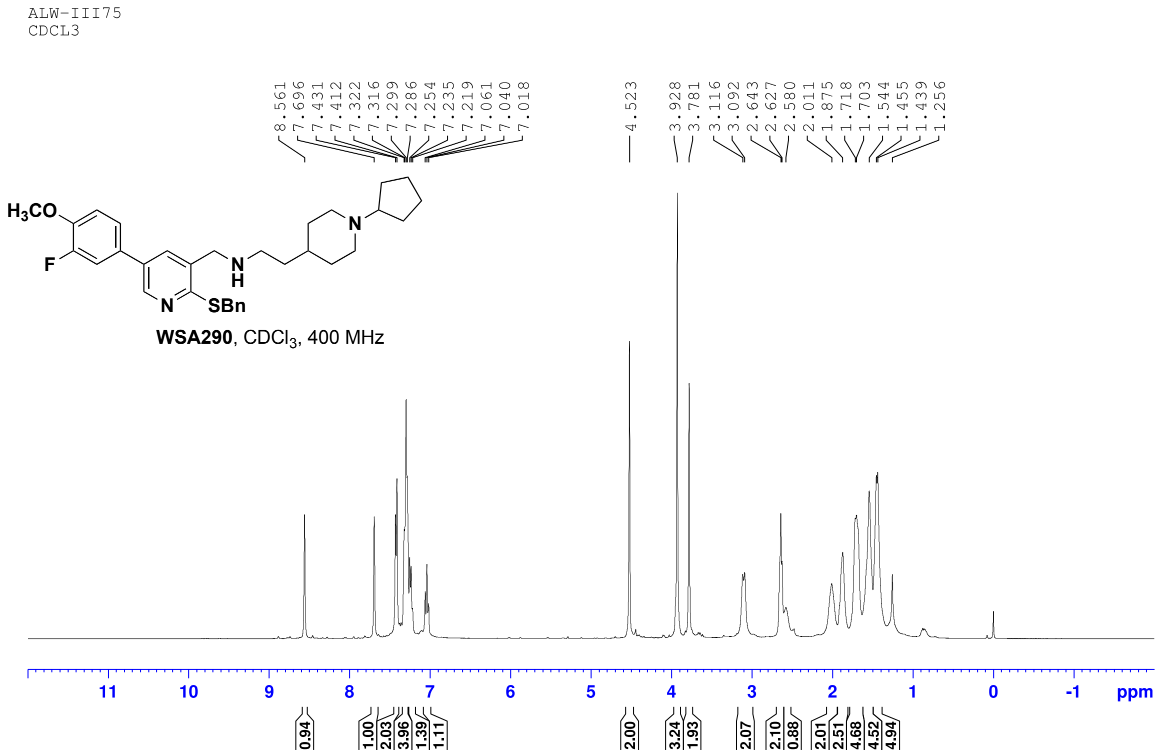

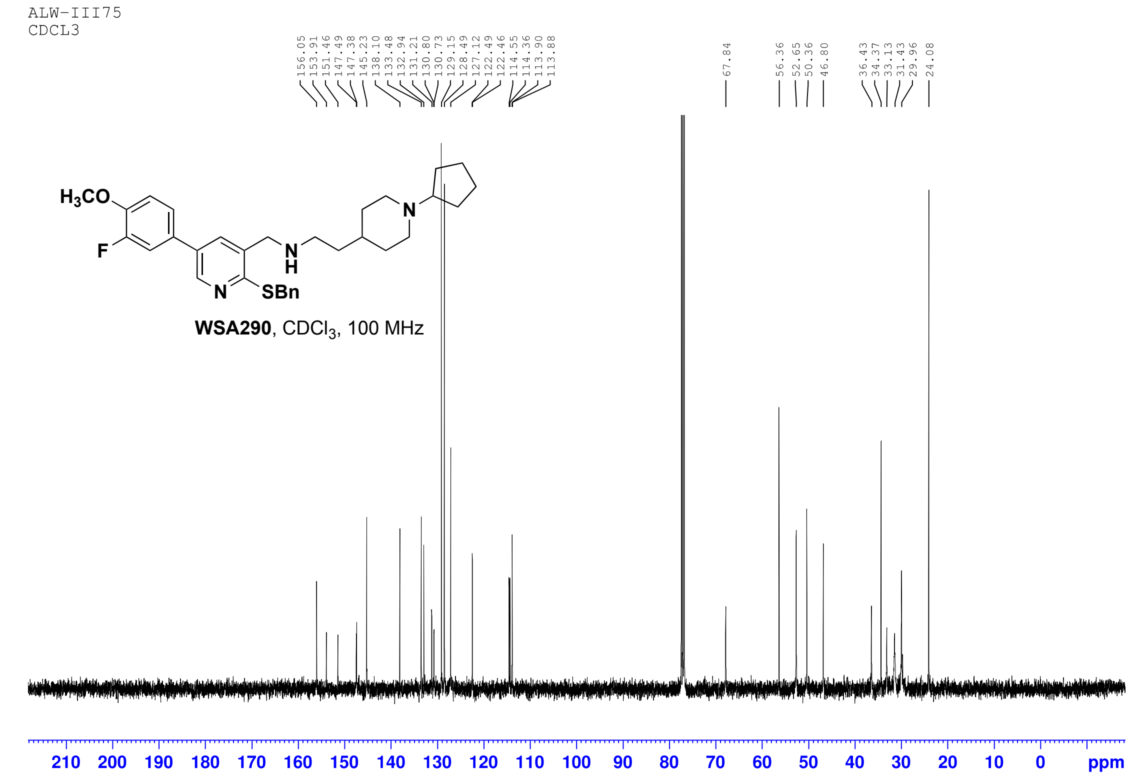

Figure S25. WSA 291 ^{1}H and ^{13}C NMR spectra.

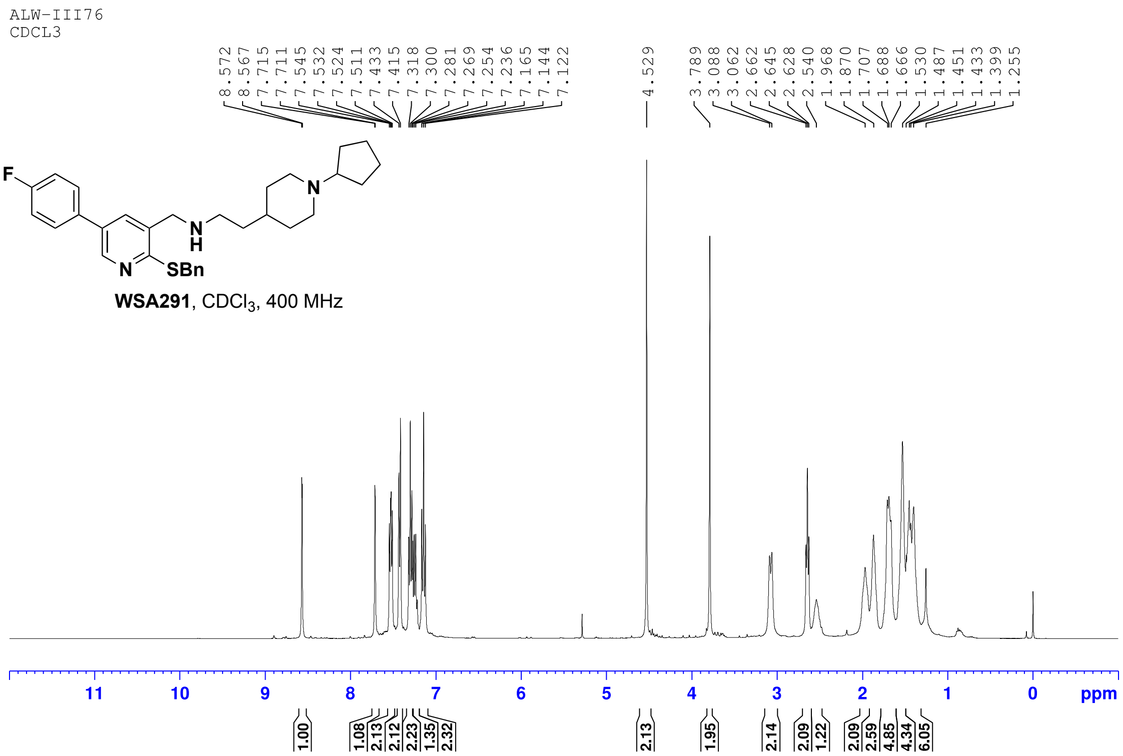

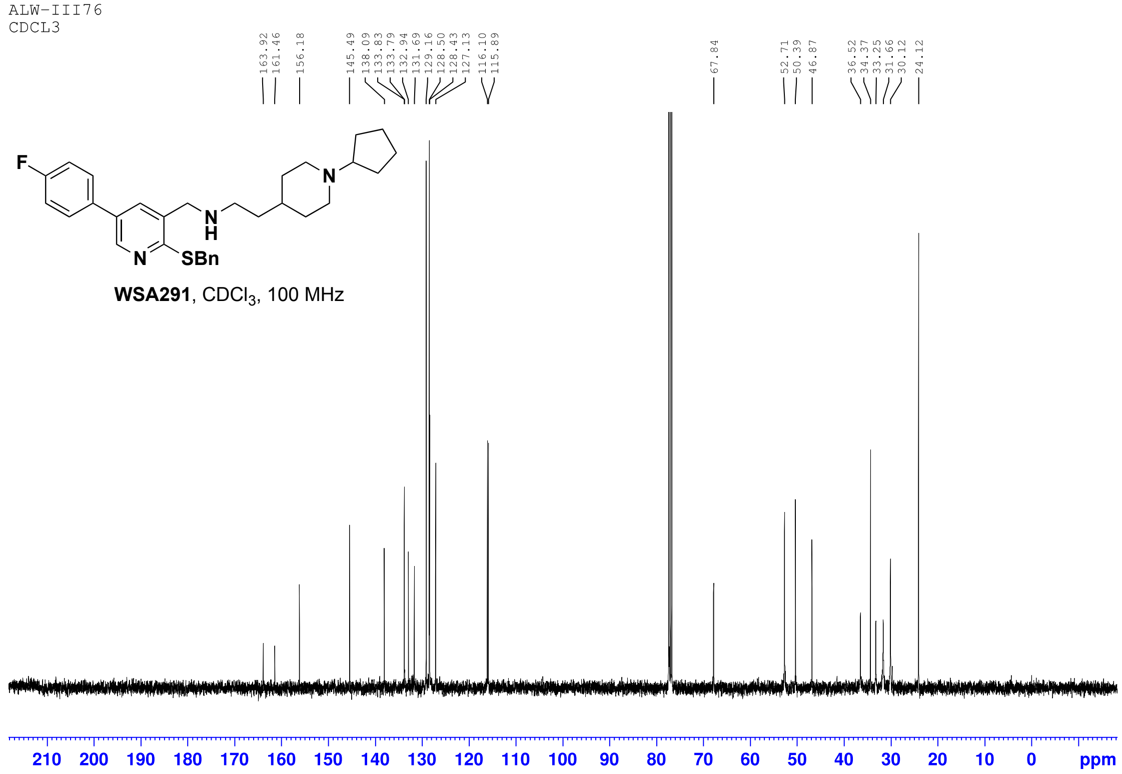

Figure S26. WSA 292 ^{1}H and ^{13}C NMR spectra.

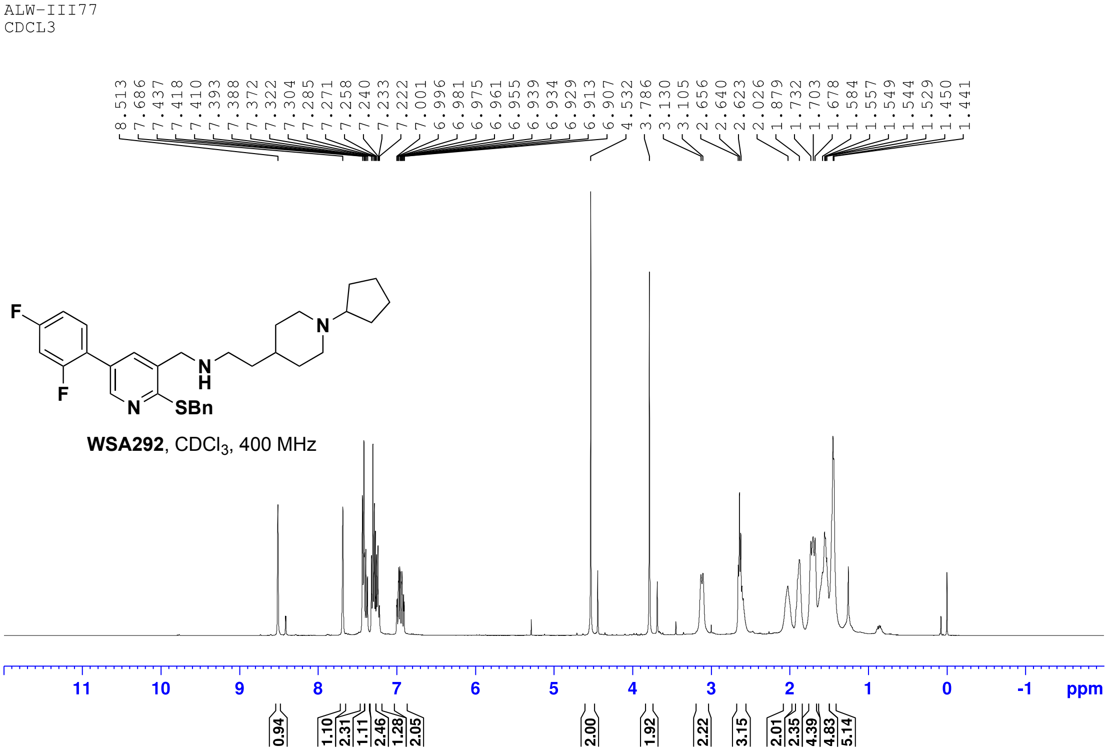

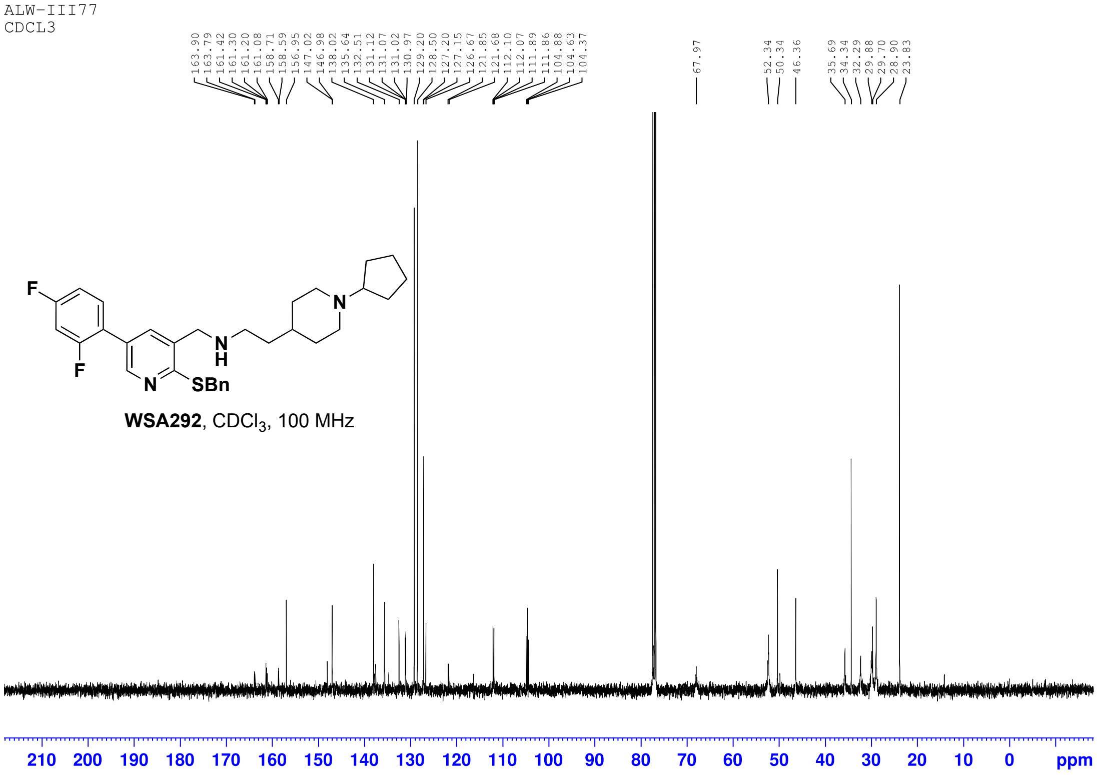

Figure S27. WSA 303 ^{1}H and ^{13}C NMR spectra.

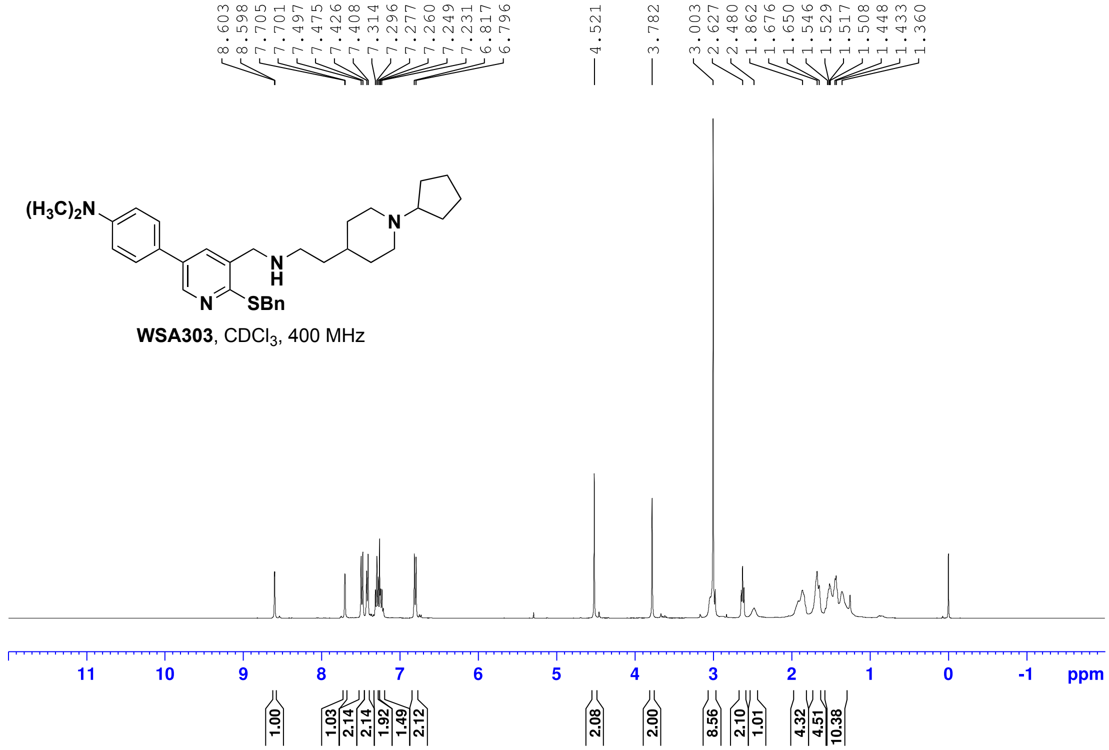

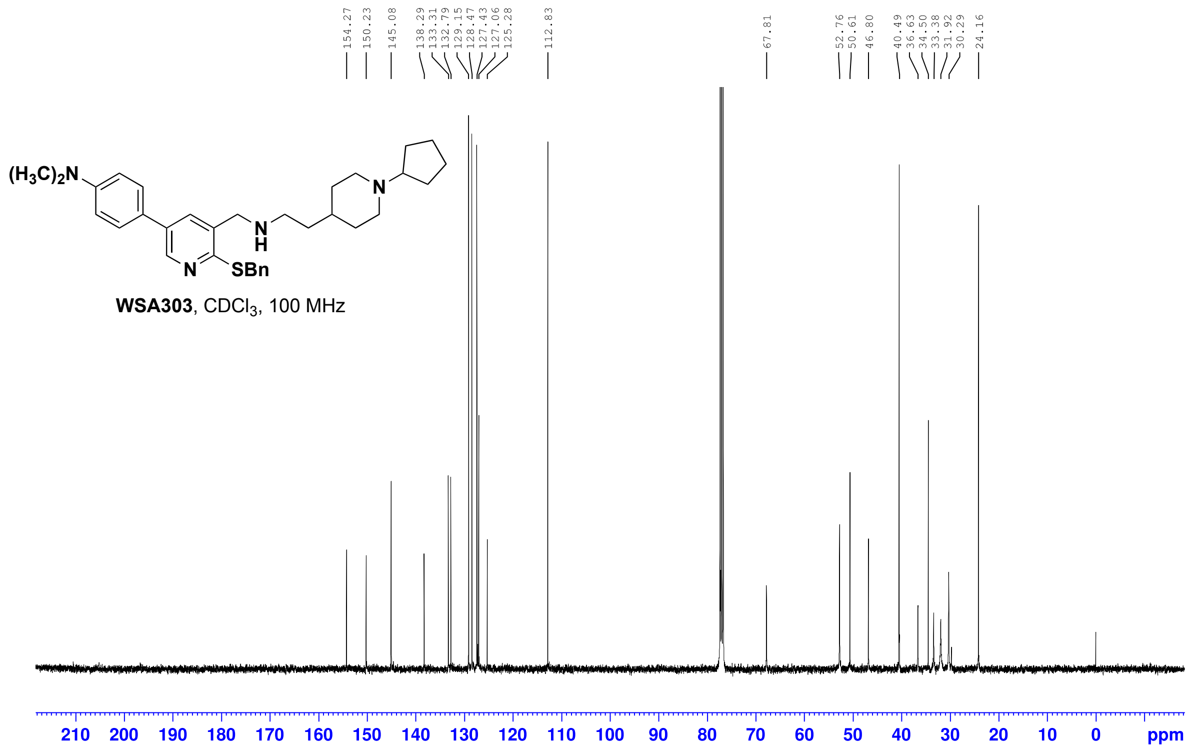

Figure S28. WSA 294 ^{1}H and ^{13}C NMR spectra.

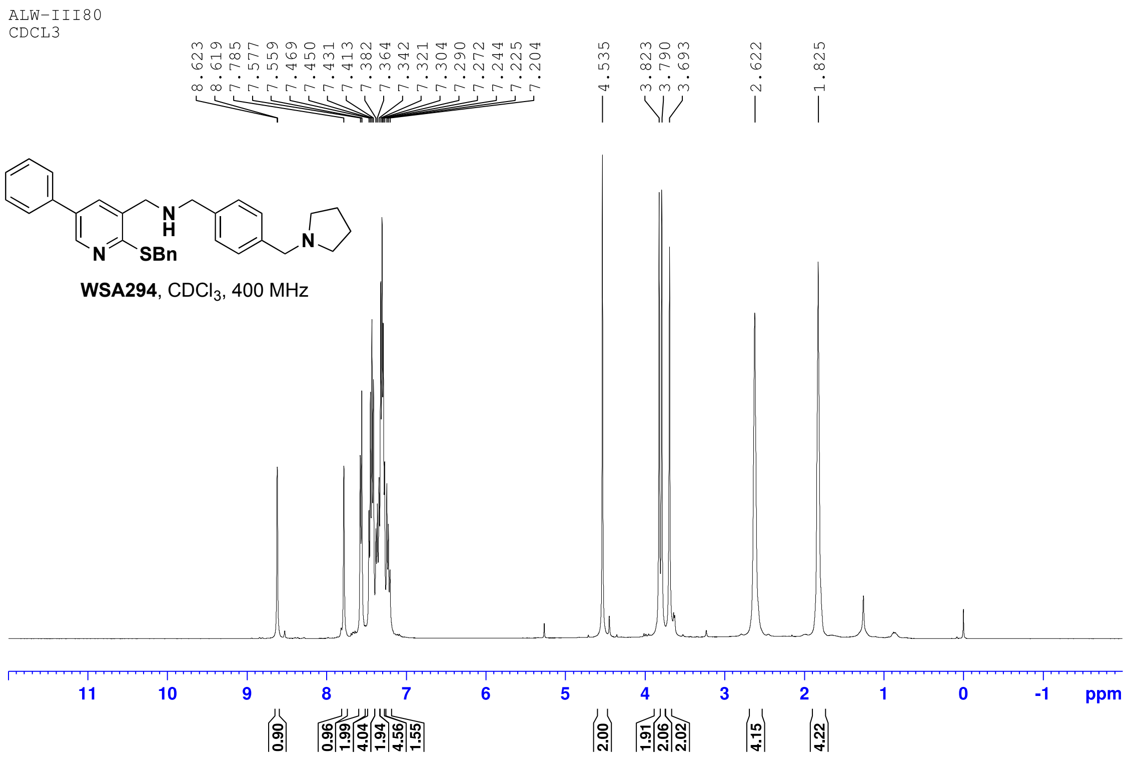
ALW-III80
CDCL3
8.623
8.619
7.785
7.577
7.559
7.469
7.450
7.431
7.413
7.382
7.364
7.342
7.321
7.304
7.290
7.272
7.244
7.225
7.204
4.535
3.823
3.790
3.693
2.622
1.825
N
H
SBn
N
WSA294, CDCl3, 400 MHz
11 10 9 8 7 6 5 4 3 2 1 0 -1 ppm
0.90
0.96
1.99
4.04
1.94
4.56
1.55
2.00
1.91
2.06
2.02
4.15
4.22

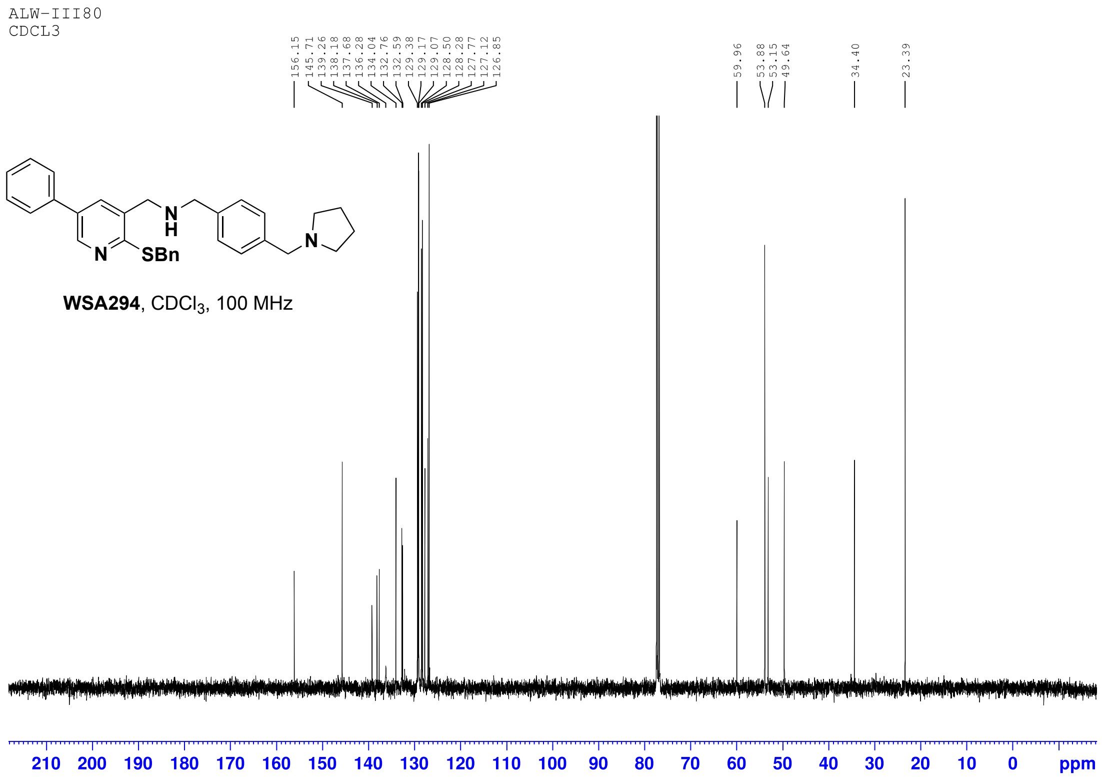
ALW-III80
CDCL3
156.15
145.71
139.26
138.18
137.68
136.28
134.04
132.76
132.59
129.38
129.17
129.07
128.50
128.28
127.77
127.12
126.85
59.96
53.88
53.15
49.64
34.40
23.39
N
H
SBn
N
WSA294, CDCl3, 100 MHz
210 200 190 180 170 160 150 140 130 120 110 100 90 80 70 60 50 40 30 20 10 0 ppm

Figure S29. WSA 295 ^{1}H and ^{13}C NMR spectra.

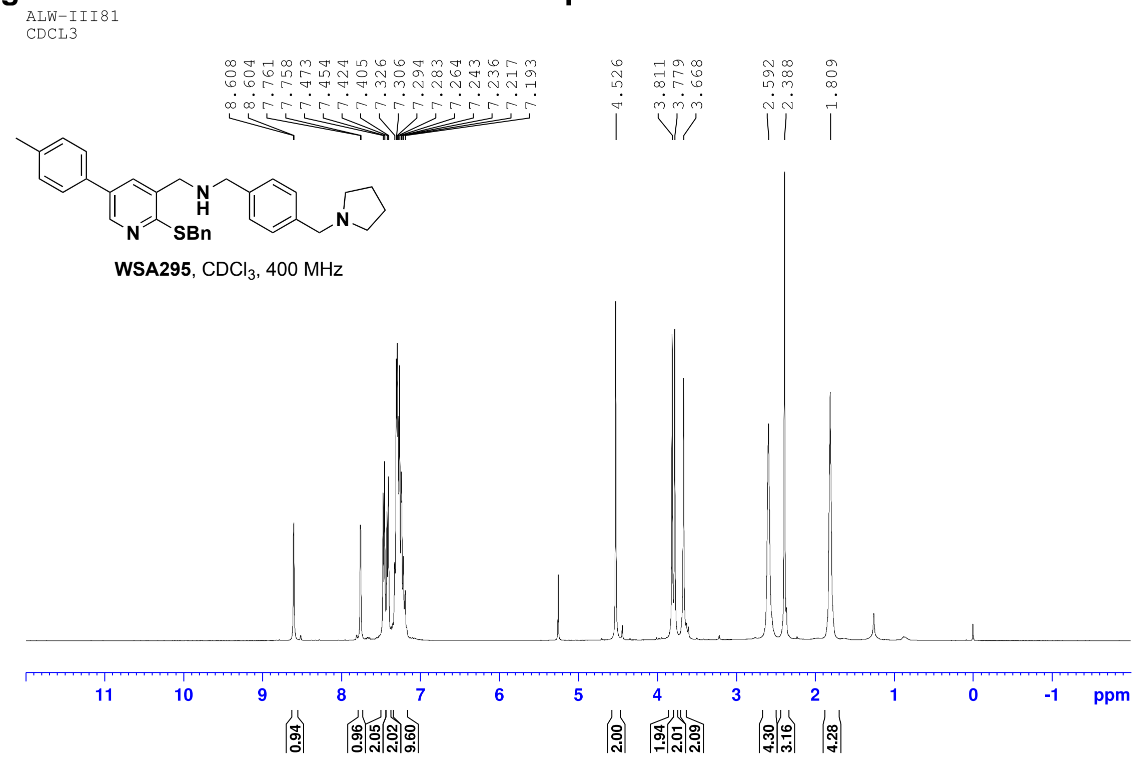

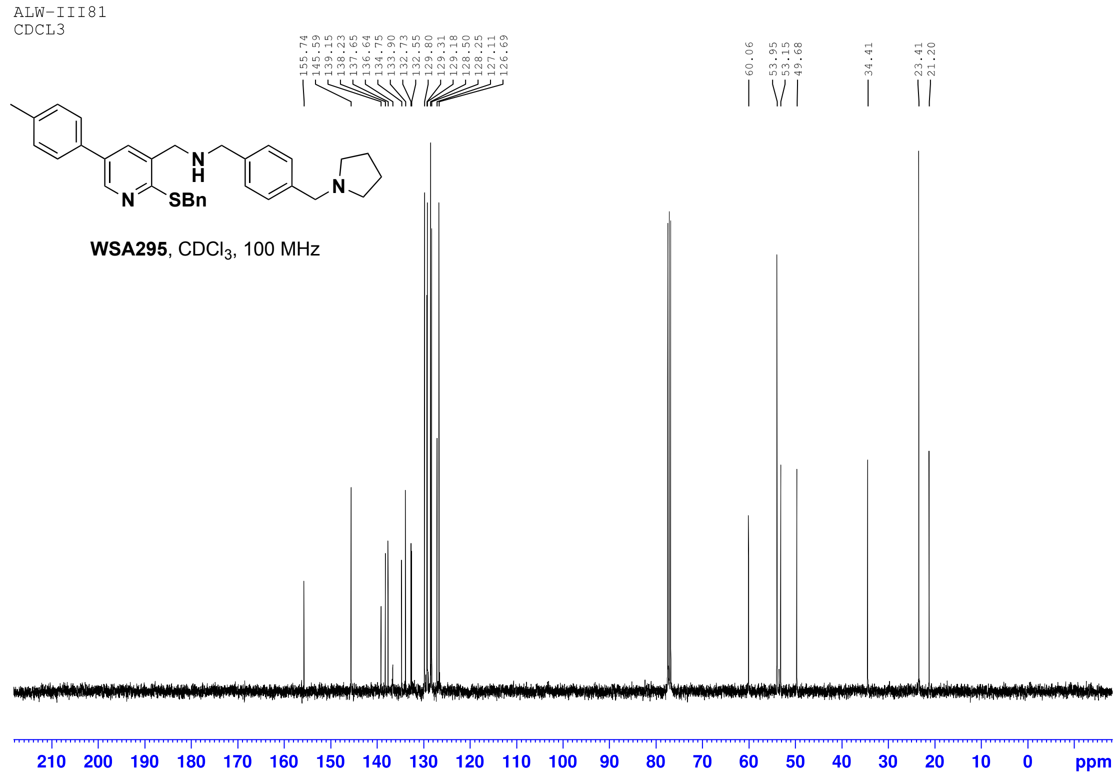

Figure S30. WSA 296 ^{1}H and ^{13}C NMR spectra.

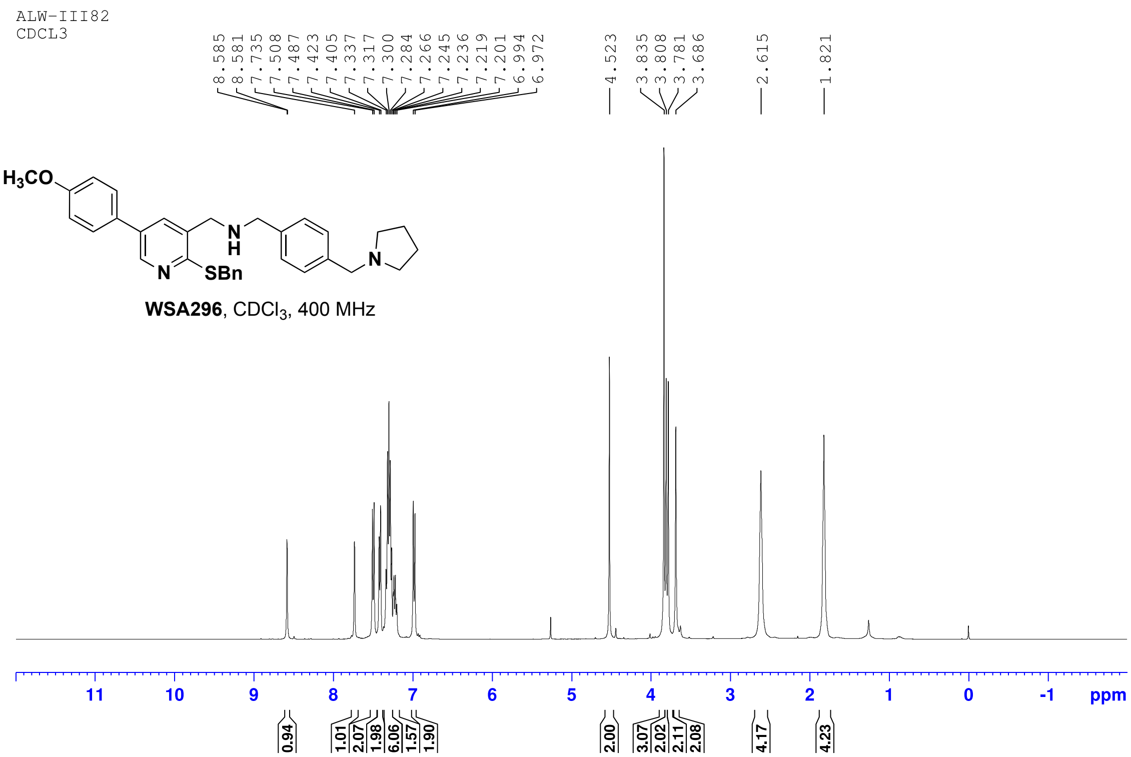
ALW-III82
CDCL3
8.585 8.581 7.735 7.508 7.487 7.423 7.405 7.337 7.317 7.300 7.284 7.266 7.245 7.236 7.219 7.201 6.994 6.972
4.523
3.835 3.808 3.781 3.686
2.615
1.821
H3CO
N
H
N
SBn
N
WSA296, CDCl3, 400 MHz
11 10 9 8 7 6 5 4 3 2 1 0 -1 ppm
0.94
1.01 2.07 1.98 6.06 1.57 1.90
2.00
3.07 2.02 2.11 2.08
4.17
4.23

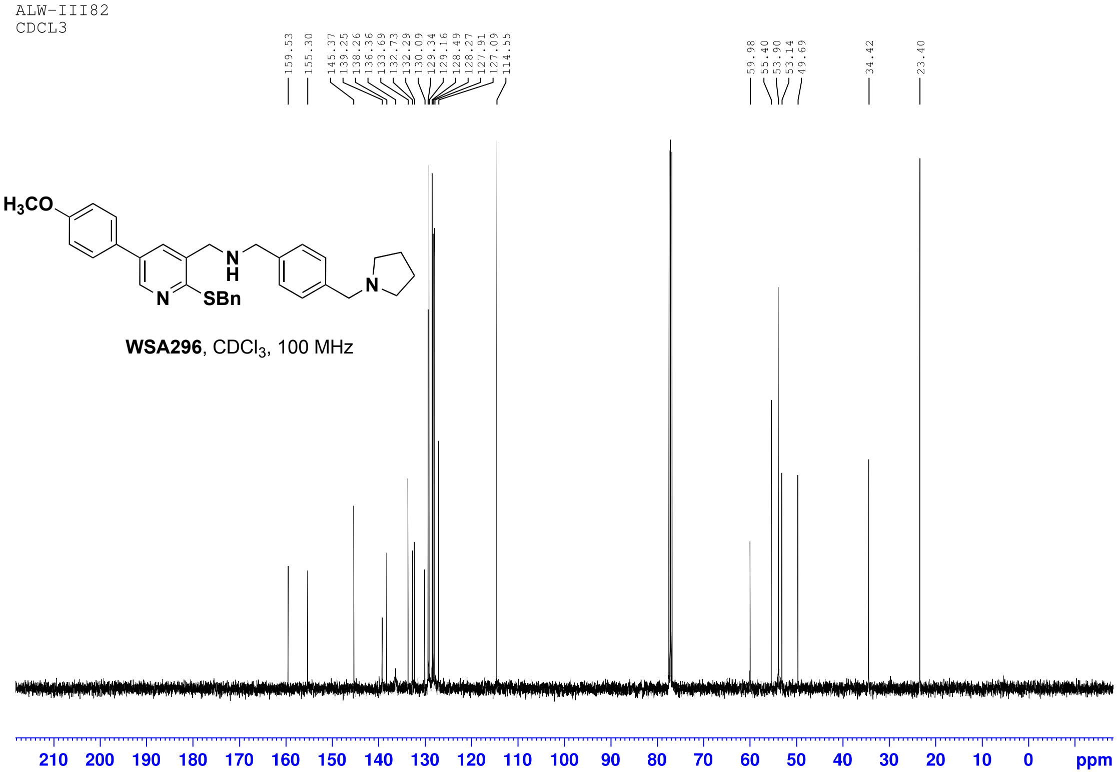
ALW-III82
CDCL3
159.53
155.30
145.37 139.25 138.26 136.36 133.69 132.73 132.29 130.09 129.34 129.16 128.49 128.27 127.91 127.09 114.55
59.98 55.40 53.90 53.14 49.69
34.42
23.40
H3CO
N
H
N
SBn
N
WSA296, CDCl3, 100 MHz
210 200 190 180 170 160 150 140 130 120 110 100 90 80 70 60 50 40 30 20 10 0 ppm

Figure S31. WSA 297 ^{1}H and ^{13}C NMR spectra.

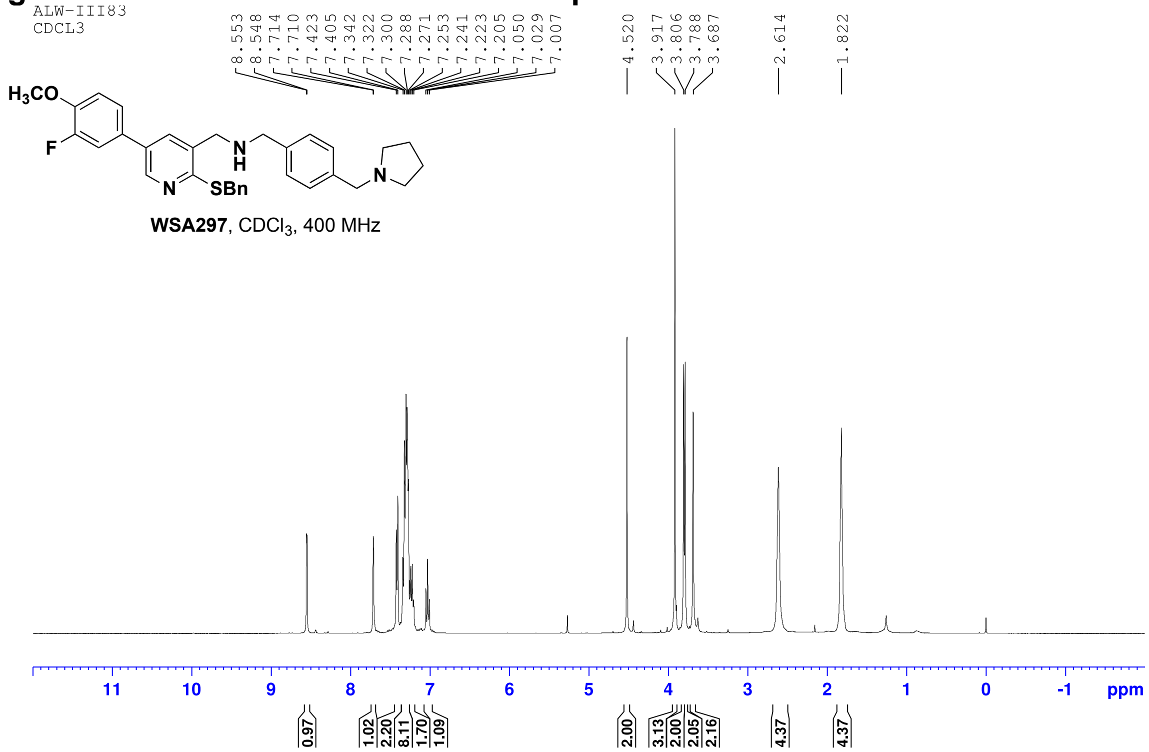

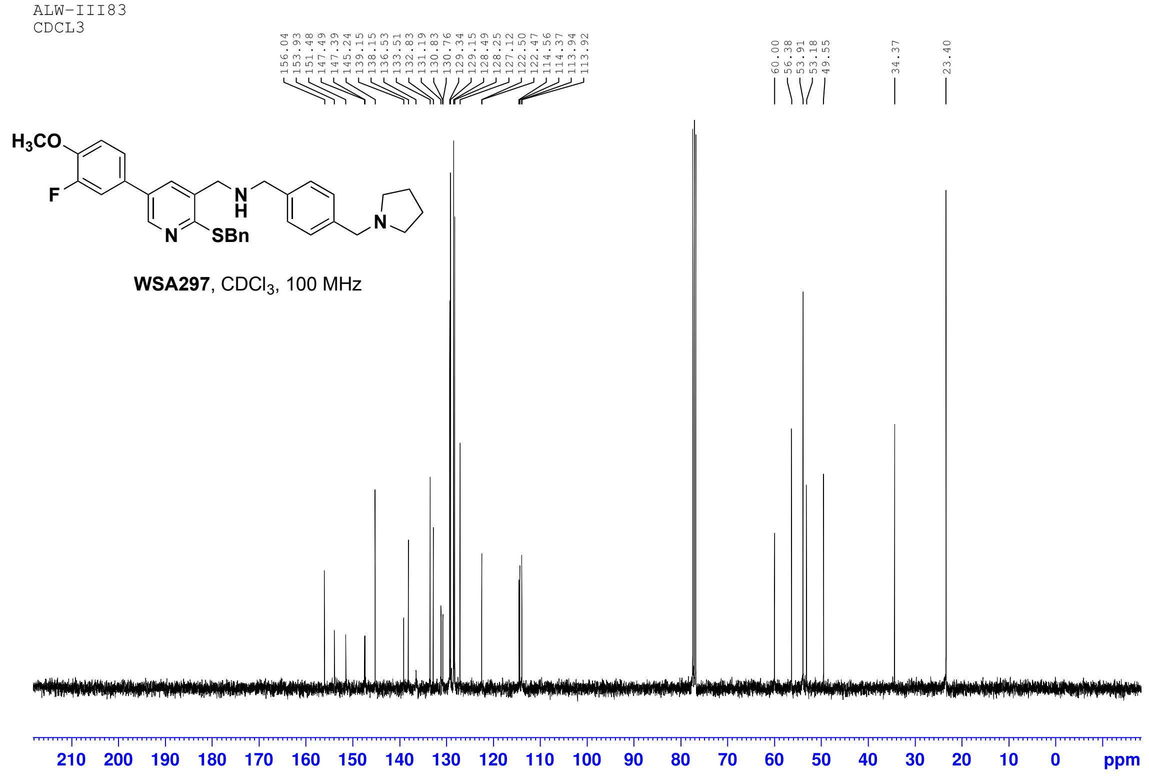

Figure S32. WSA 298 ^{1}H and ^{13}C NMR spectra.

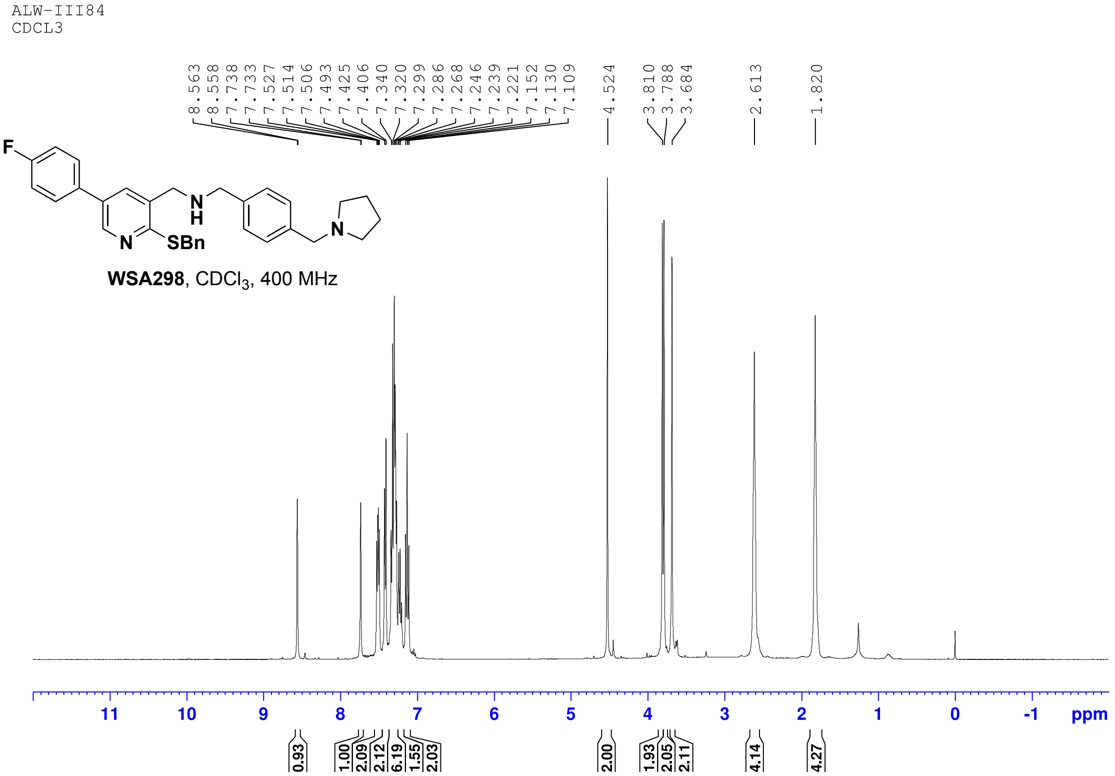

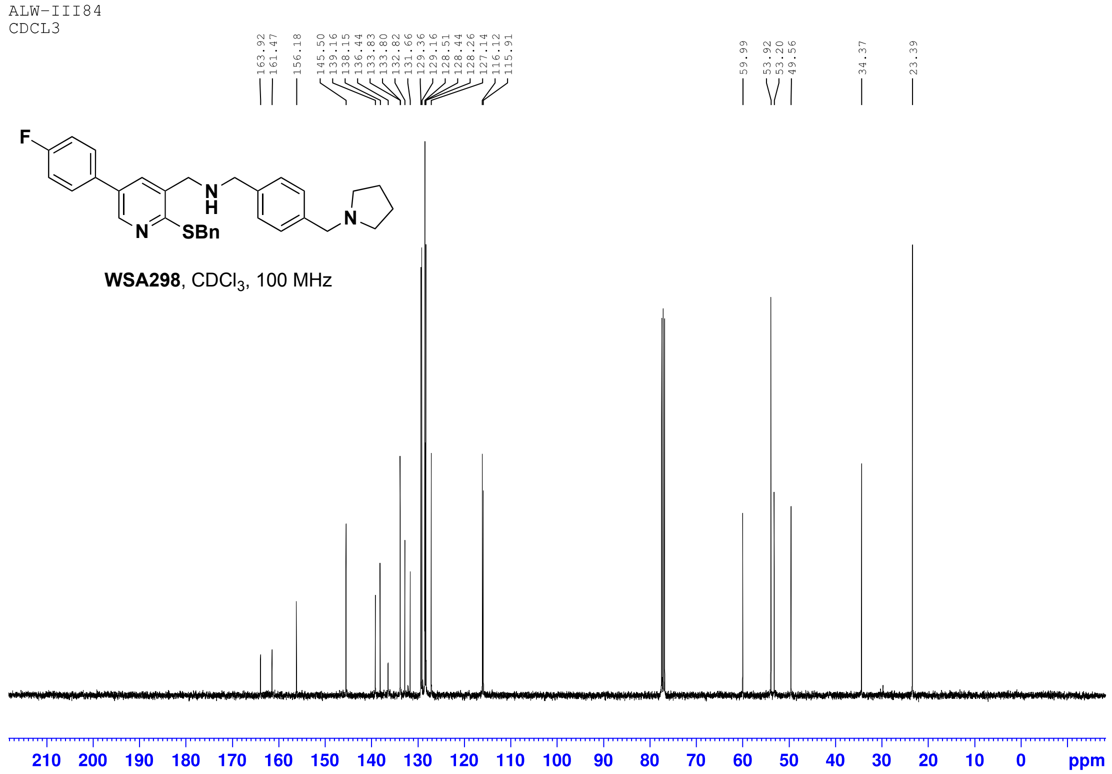

Figure S33. WSA 299 ^{1}H and ^{13}C NMR spectra.

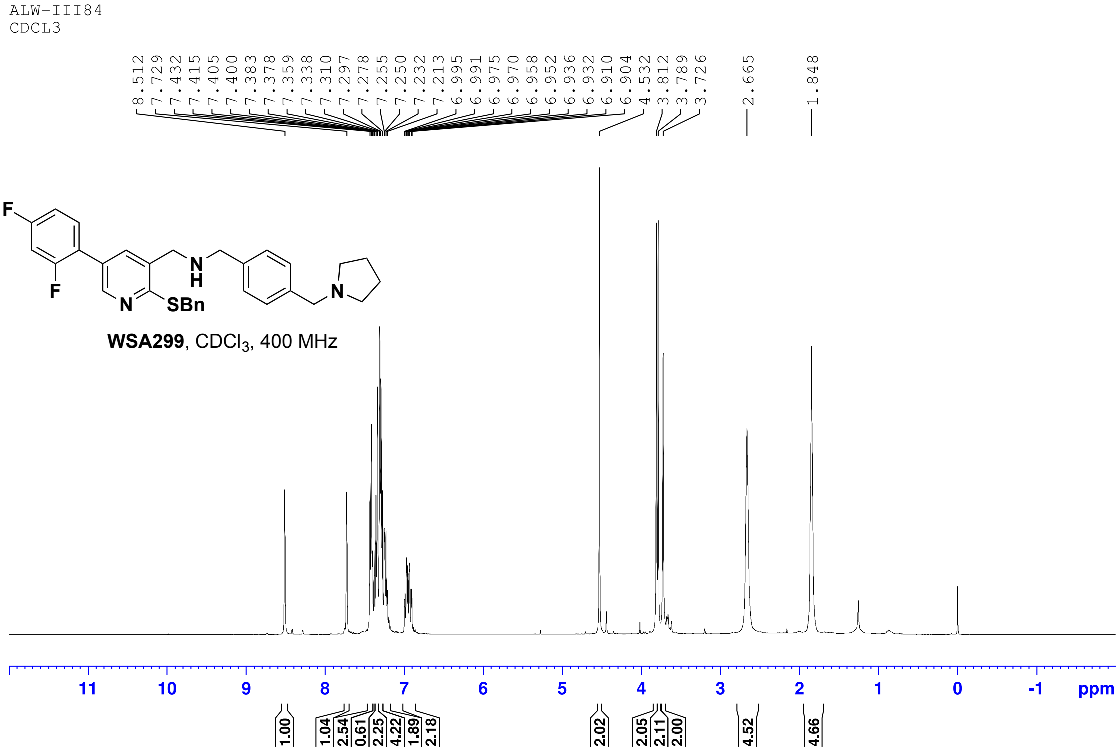

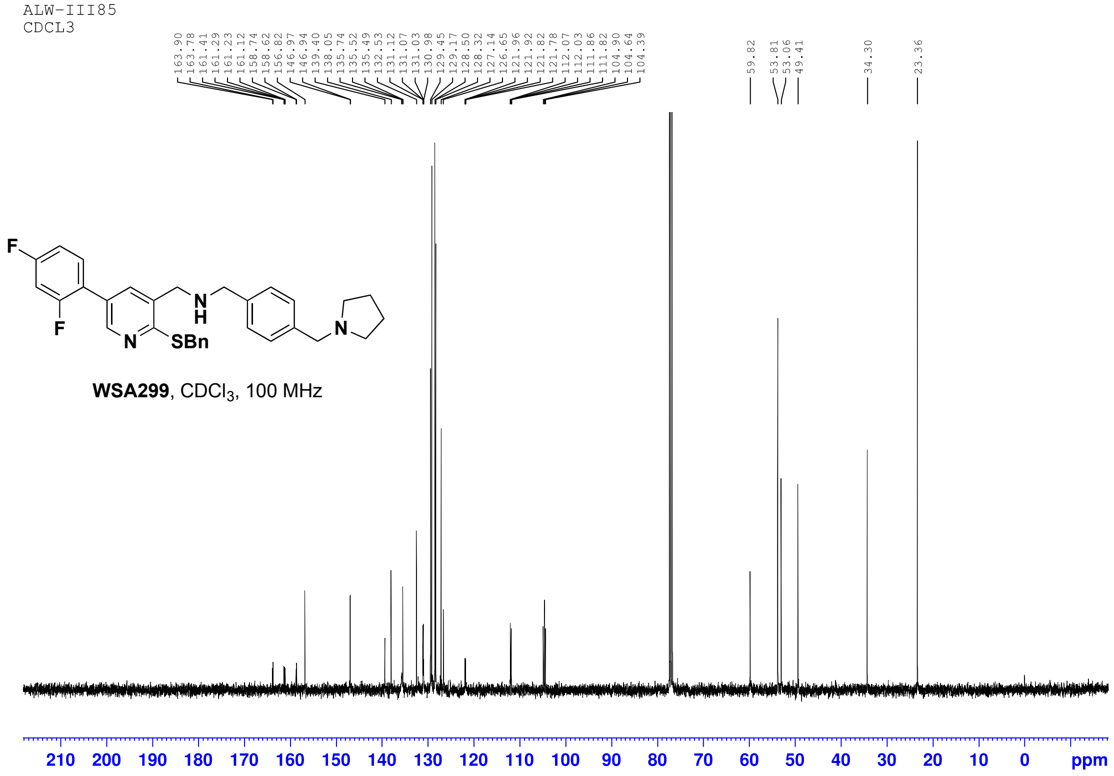

Figure S34. WSA 304 ¹H and ¹³C NMR spectra.

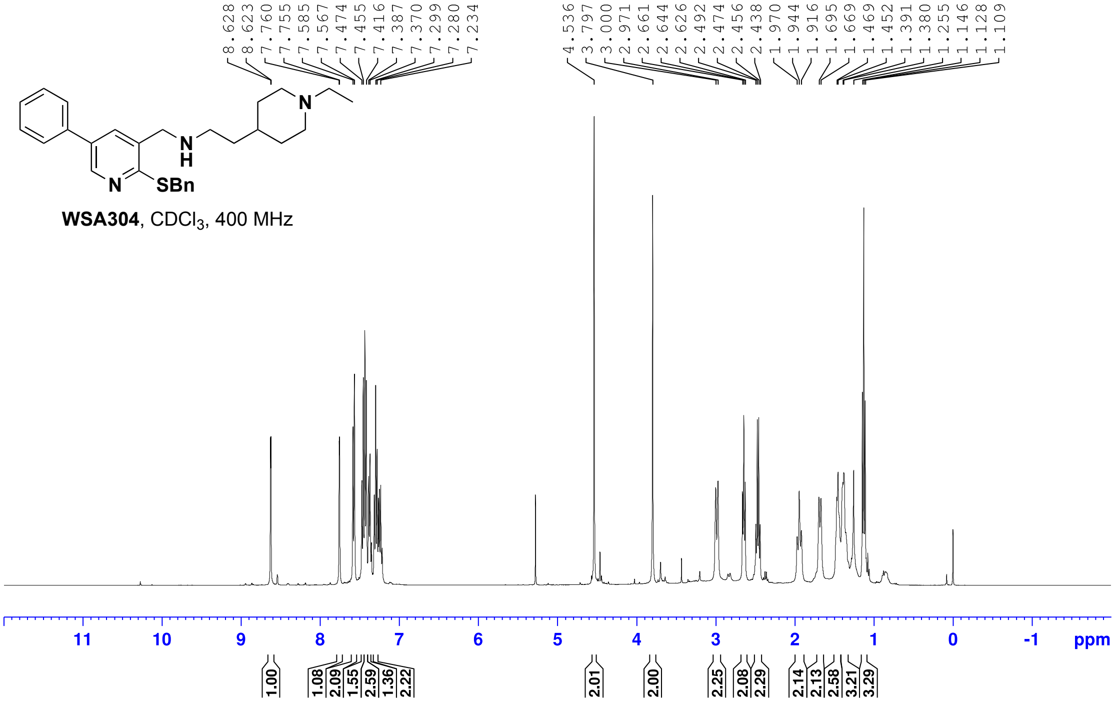

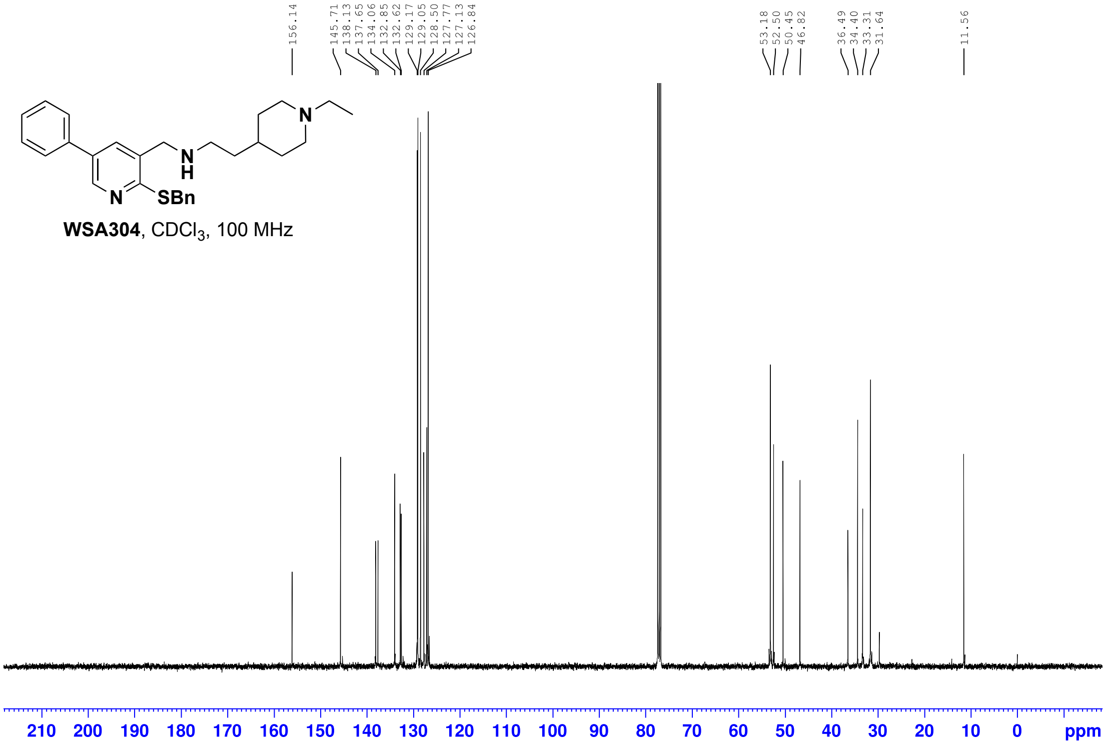

Figure S35. WSA 300 ^{1}H and ^{13}C NMR spectra.

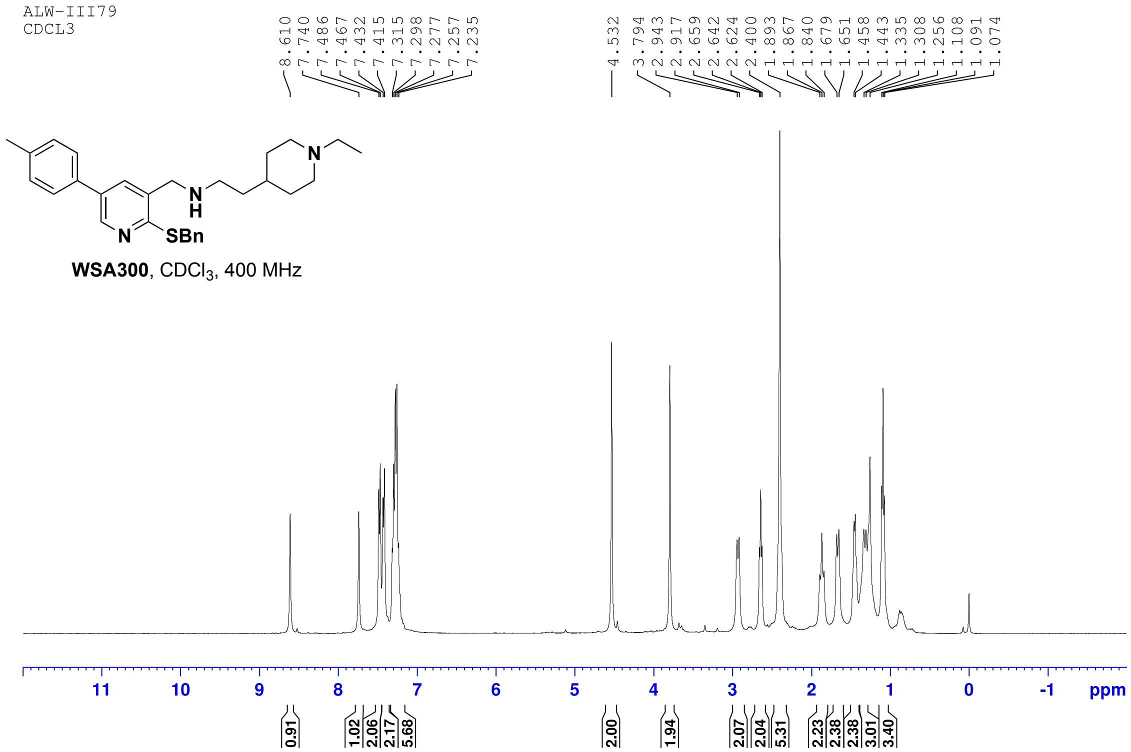

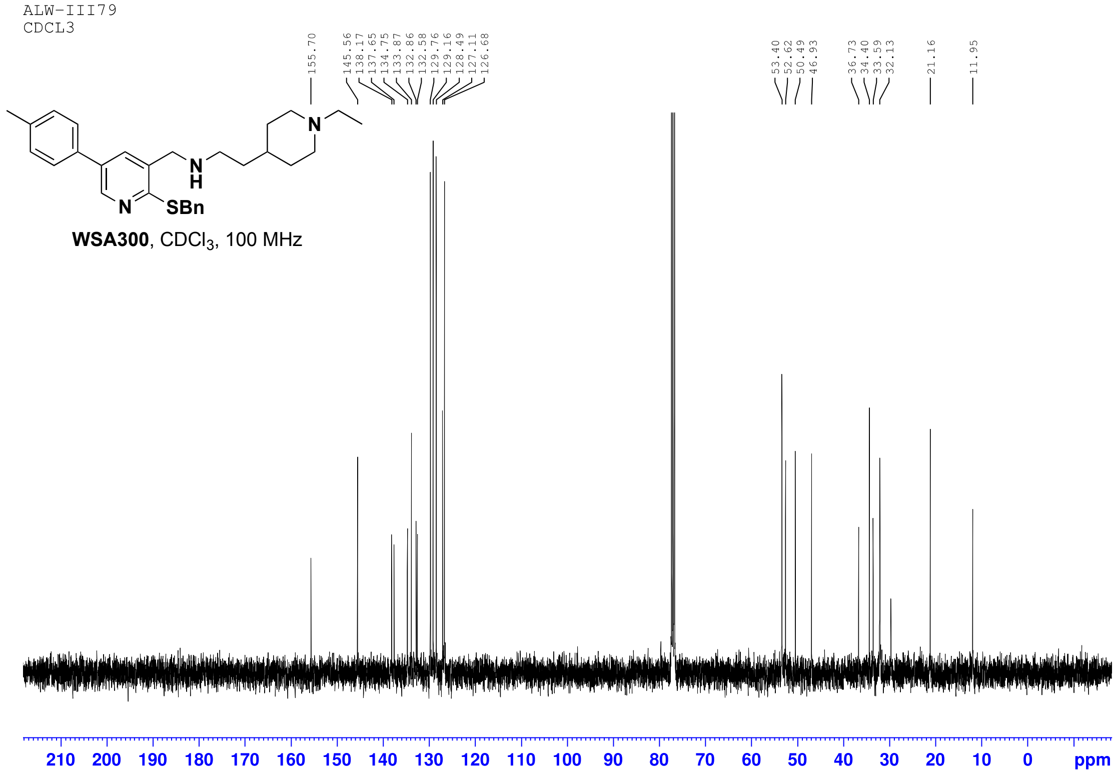

Figure S36. WSA 305 ^{1}H and ^{13}C NMR spectra.

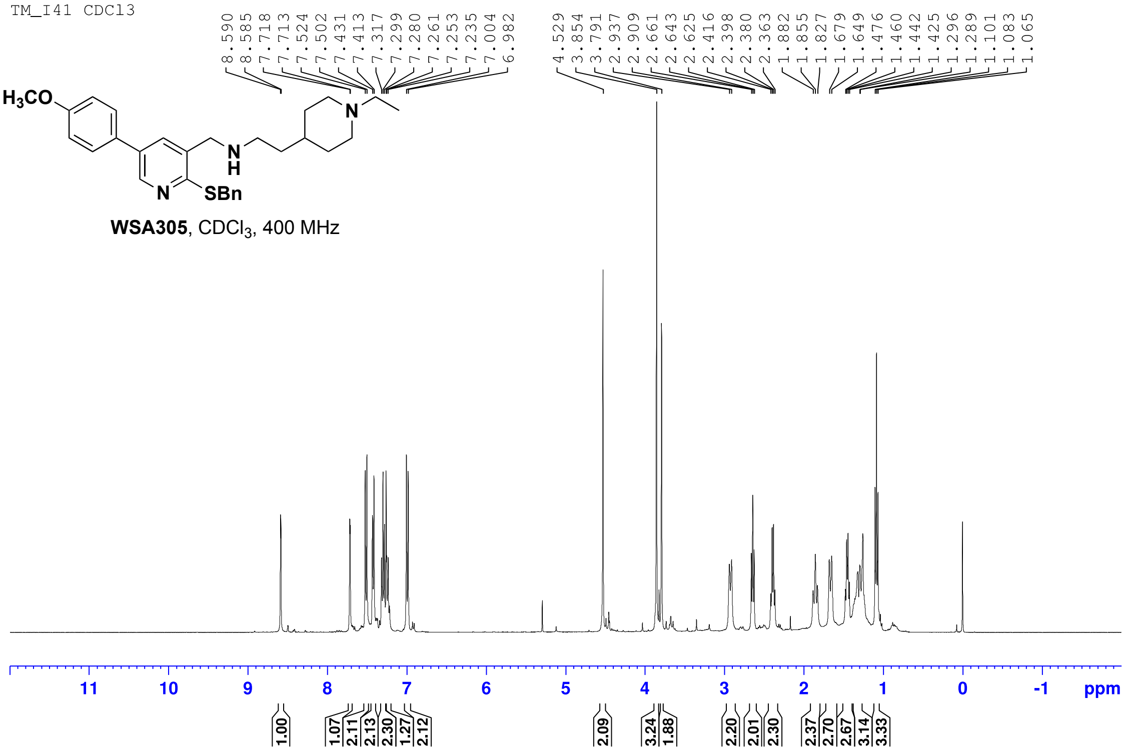

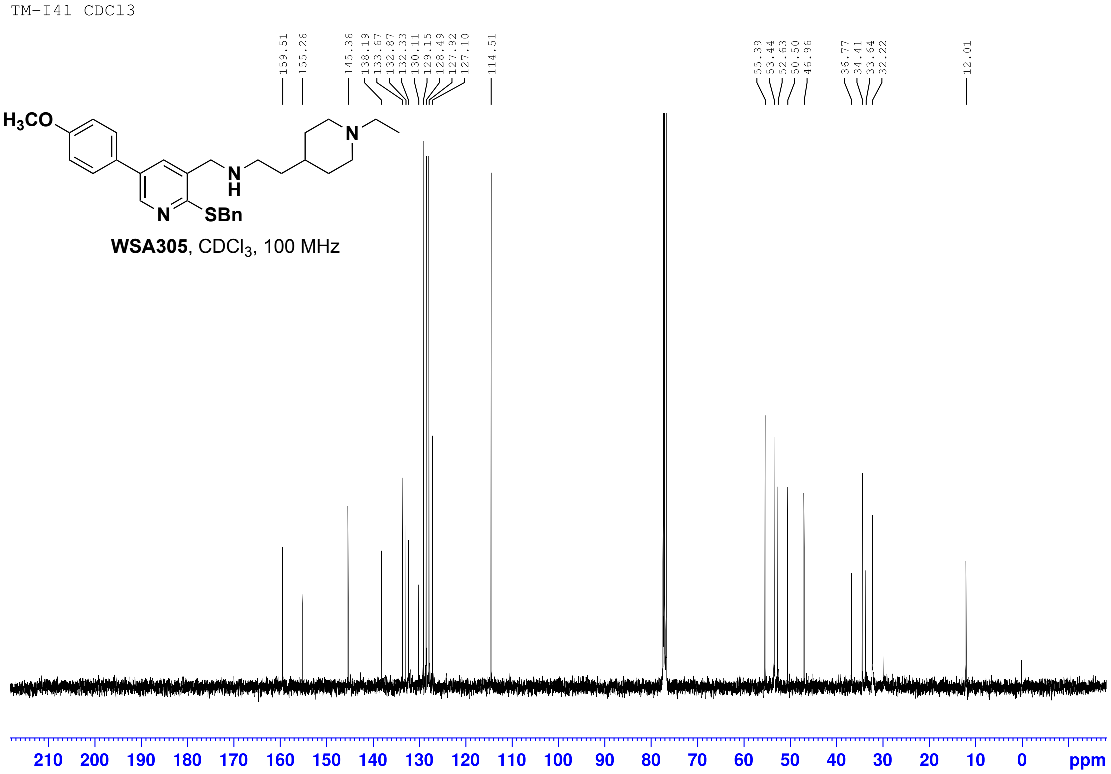

Figure S37. WSA 306 ^{1}H and ^{13}C NMR spectra.

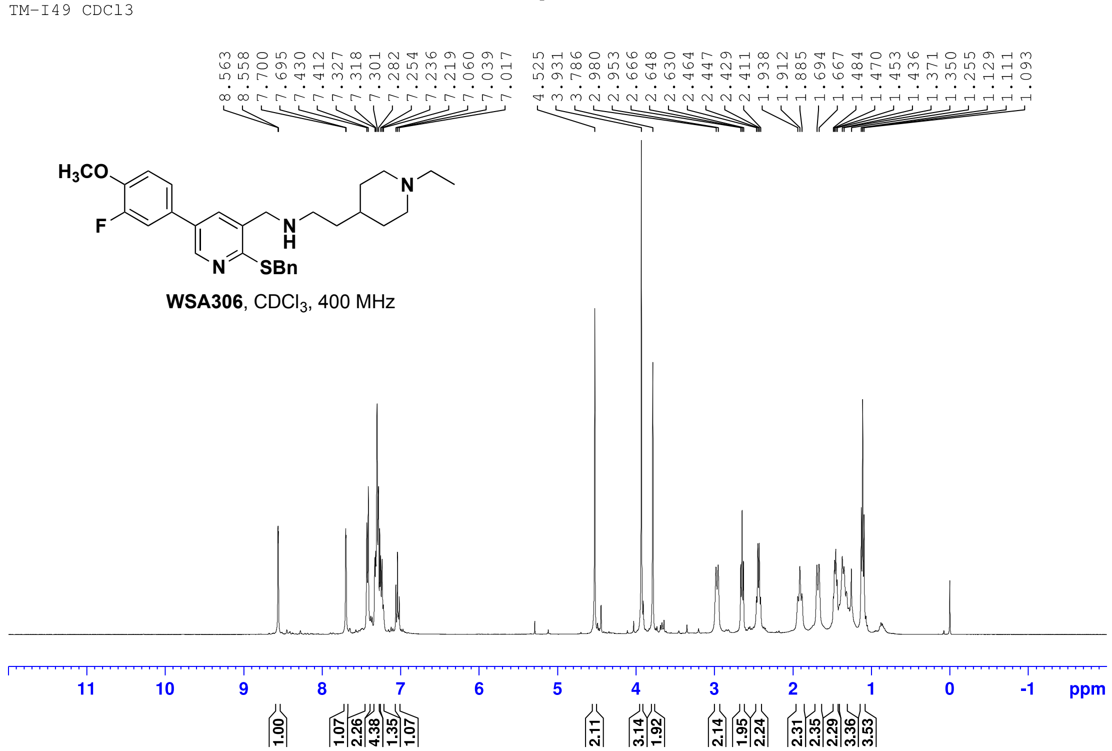

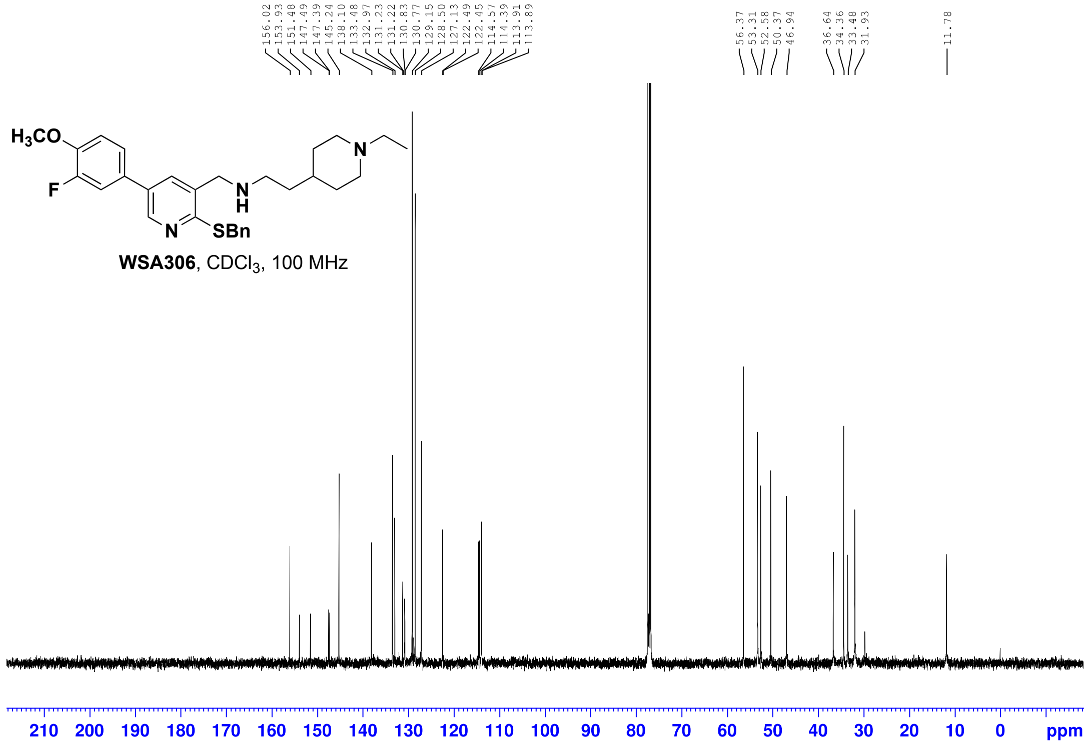

Figure S38. WSA 307 ^{1}H and ^{13}C NMR spectra.

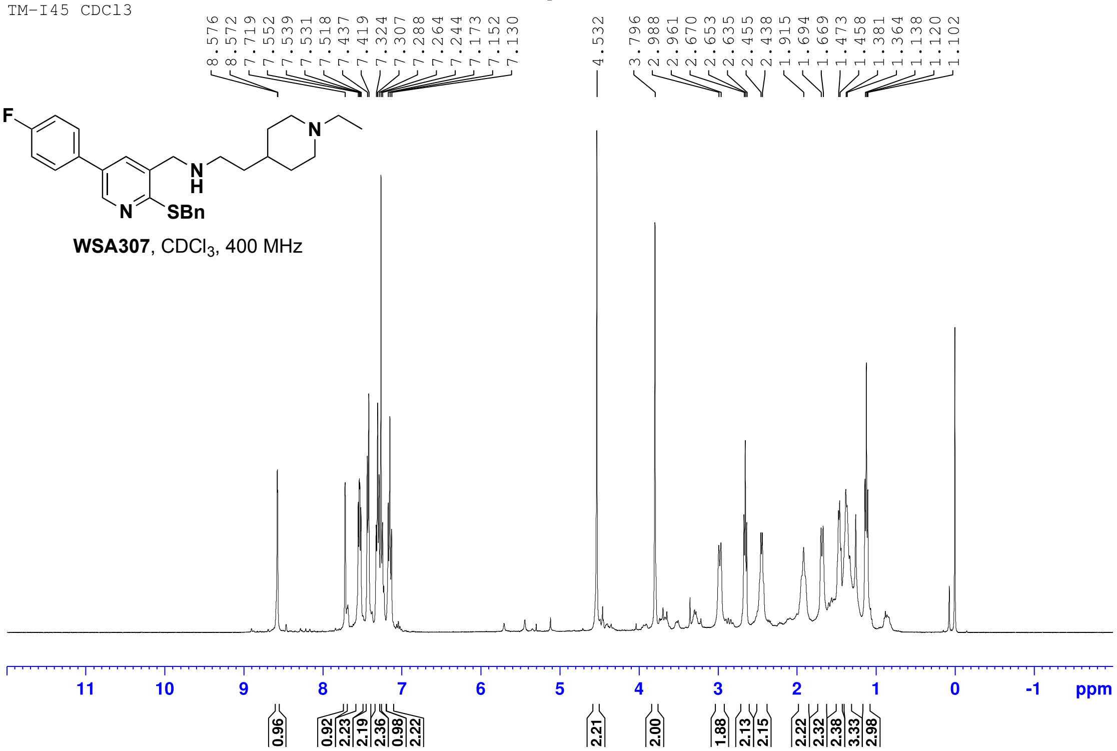

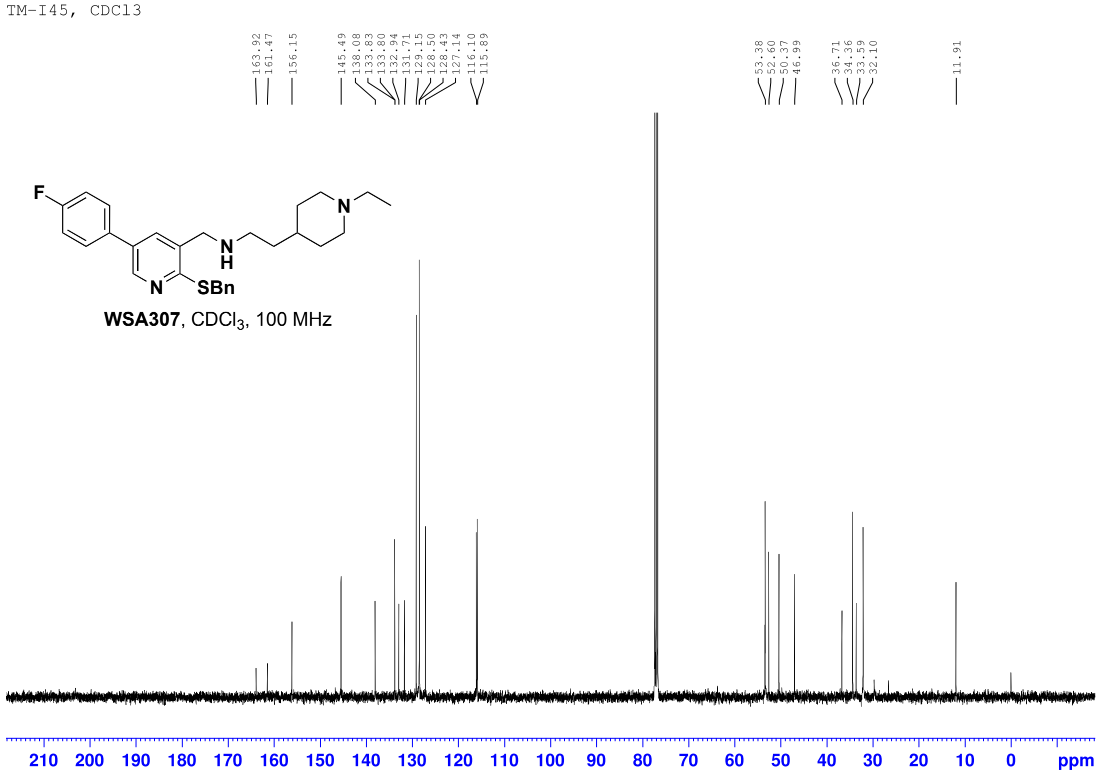

Figure S39. WSA 308 ^{1}H and ^{13}C NMR spectra.

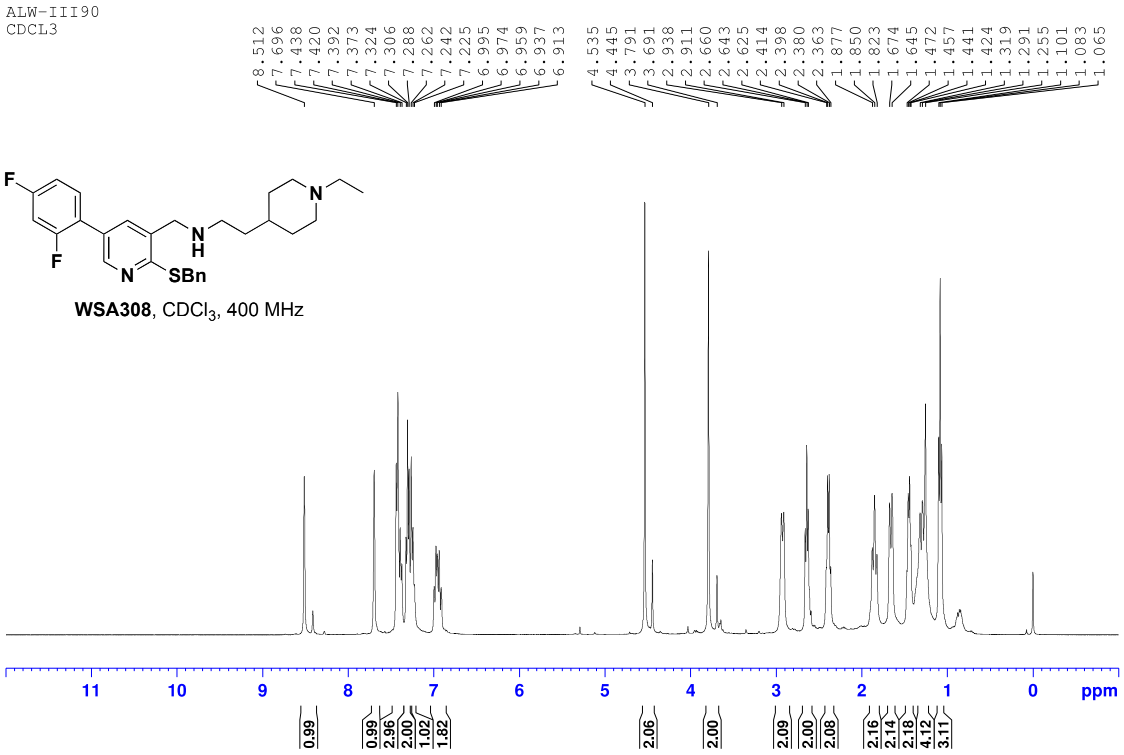

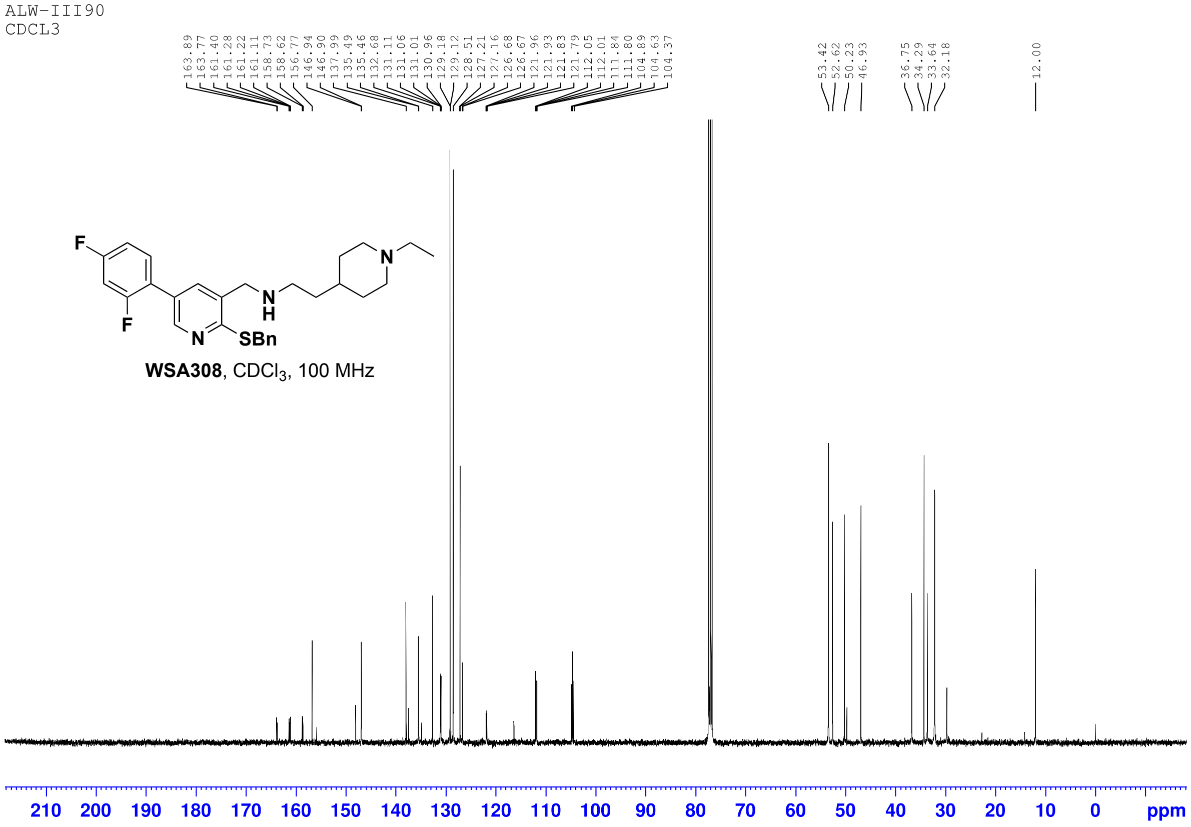

Supplement: Supplementary file 1 [file ao5c06380_si_001.pdf]
